# Delving in folate metabolism in the parasite *Leishmania major* through a chemogenomic screen and methotrexate selection

**DOI:** 10.1371/journal.pntd.0011458

**Published:** 2023-06-29

**Authors:** Sophia Bigot, Philippe Leprohon, Marc Ouellette

**Affiliations:** Centre de Recherche en Infectiologie du Centre de Recherche du CHU de Québec and Département de Microbiologie, Infectiologie et Immunologie, Faculté de Médecine, Université Laval, Quebec City, Québec, Canada; Universiteit Antwerpen, BELGIUM

## Abstract

Most of our understanding of folate metabolism in the parasite *Leishmania* is derived from studies of resistance to the antifolate methotrexate (MTX). A chemical mutagenesis screen of *L*. *major* Friedlin and selection for resistance to MTX led to twenty mutants with a 2- to 400-fold decrease in MTX susceptibility in comparison to wild-type cells. The genome sequence of the twenty mutants highlighted recurrent mutations (SNPs, gene deletion) in genes known to be involved in folate metabolism but also in novel genes. The most frequent events occurred at the level of the locus coding for the folate transporter FT1 and included gene deletion and gene conversion events, as well as single nucleotide changes. The role of some of these *FT1* point mutations in MTX resistance was validated by gene editing. The gene *DHFR-TS* coding for the dihydrofolate reductase-thymidylate synthase was the second locus with the most mutations and gene editing confirmed a role in resistance for some of these. The pteridine reductase gene *PTR1* was mutated in two mutants. The episomal overexpression of the mutated versions of this gene, but also of *DHFR-TS*, led to parasites several fold more resistant to MTX than those overexpressing the wild-type versions. Genes with no known link with folate metabolism and coding for a L-galactolactone oxidase or for a methyltransferase were mutated in specific mutants. Overexpression of the wild-type versions of these genes in the appropriate mutants reverted their resistance. Our Mut-seq approach provided a holistic view and a long list of candidate genes potentially involved in folate and antifolate metabolism in *Leishmania*.

## Author summary

Studies of the resistance to the antifolate methotrexate (MTX) in the protozoan parasite *Leishmania* have allowed to improve the understanding of one carbon metabolism. Here, through a chemogenomic screen with MTX selection, we generated 20 mutants with a decrease of MTX susceptibility. We reported some point mutations in genes known to be associated with MTX resistance (*FT1*, *DHFR-TS* and *PTR1*) but also in genes never associated with it, such as *ALO* and the methyltransferase LmjF.17.1130. Gene editing studies validated that point mutations in *FT1* and *DHTR-TS* can be phenotypic in the context of MTX resistance. Some *DHFR-TS* and *PTR1* mutations are further shown to have a dominant positive effect on resistance when overexpressed. This contrasts with the mutations detected in the gene *ALO* and in the methyltransferase LmjF.17.1130, for which overexpression of the wild-type versions reverted the MTX resistance they conferred. Overall, this screen has revealed several new mutations involved in antifolate resistance in *Leishmania*.

## Introduction

The protozoan parasite *Leishmania* is distributed globally and is the cause of various forms of leishmaniasis. Treatments are limited and no vaccine is available for humans [1]. The understanding of essential metabolic pathways is critical as this may allow the development of improved chemotherapies. Folic acid metabolism in *Leishmania* is sufficiently different than their hosts that specific inhibitors of this pathway may eventually be found [2,3]. *Leishmania* is a folate auxotroph and it encodes a number of membrane transporters, including the main folate transporter FT1, that belong to the Folate Biopterin Transporter family [4]. Folates are reduced by the bifunctional dihydrofolate reductase-thymidylate synthase (DHFR-TS) and are essential co-factors in thymidine synthesis [2,3]. Most of our understanding of folate metabolism in *Leishmania* are derived from studies on the mechanisms of resistance to the model antifolate drug methotrexate (MTX). Indeed, resistance to MTX led to the discovery of the amplification of *DHFR-TS* [5–7] and of the deletion of *FT1* [8]. Studies of MTX resistance also led to the discovery of the pteridine reductase 1 (PTR1), whose gene amplification is common in MTX-resistant *Leishmania* [9,10]. Several other enzymes of the folate pathway were also shown to modulate MTX susceptibility. This includes the folylpolyglutamate synthase [11], the methionine adenosyl transferase [12], the serine hydroxymethyltransferase [13] and the mitochondrial glycine cleavage complex (*GCC*) [14].

While several of the markers of resistance were historically isolated by gene amplification studies [5,15], more recently a number of genomic screens using MTX resistance as a selection strategy were used to further our understanding of folate metabolism in *Leishmania* [16–18]. In the current study we applied Mut-Seq [19], a chemogenomic screen that couples chemical mutagenesis to next generation sequencing and that we recently adapted to *Leishmania* [20], with MTX selection to further our understanding of one carbon metabolism in *Leishmania*. We highlighted several candidate resistance genes by sequencing the genome of 20 MTX-resistant clones derived from the Mut-seq screen. We also confirmed the role of several of these genes in resistance to MTX by focusing on recurrent mutations that were studied by gene editing and/or gene transformation.

## Methods

### Cell culture

*L*. *major* Friedlin parasites were maintained as promastigotes at 25°C in M199 medium supplemented with 10% (vol/vol) heat-inactivated fetal bovine serum, 5 μg/mL hemin and 5 μM biopterin. Drug activity in the promastigote stage was determined by measuring the OD_600_ after 72h of exposure to a range of drug concentrations in a multi-well scanning spectrophotometer (Thermo Labsystems Multiskan Spectrum UV/visible Microplate Reader) as described [21].

### Chemical mutagenesis

A clonal population of *L*. *major* Friedlin was inoculated into two T25 flasks and incubated at 25°C. For one flask, cells were mutagenized with 40 mM ethyl methanesulfonate (EMS) for 6 hours at 25°C as described [20]. Both cultures were washed in 1× Hepes-NaCl and transferred into fresh M199 medium for 24h to 72h until parasite recovery. For both cultures, 5×10^7^ parasites were spread on M199 agar plates supplemented with 10% (vol/vol) heat-inactivated FBS, 5 μg/mL hemin, 5 μM biopterin and containing 5× or 20× the MTX EC_50_ of *L*. *major* Friedlin wild-type. Plates were incubated at 25°C for 14 to 21 days. EMS-mutagenized *Leishmania* clones growing on MTX plates were grown individually in M199 medium without drug pressure at 25°C. Their level of resistance to MTX was evaluated and their MTX EC_50_ compared to the source clone of *L*. *major* Friedlin.

### Whole-genome sequencing and analysis

Genomic DNA was extracted from mid-log phase cultures of *L*. *major* Friedlin mutants and the wild-type parent. Next-generation sequencing (NGS) libraries were prepared using the Illumina DNA prep kit and sequenced on a NovaSeq6000 sequencer (Illumina). Sequence reads were trimmed according to their base quality using the trimmomatic software [22] and aligned to the *L*. *major* Friedlin reference genome (TritrypDB release 48) with the BWA software [23]. Reads duplicates were marked using Picard and GATK was used to identify single nucleotide polymorphism (SNPs) and small insertions or deletions (InDels) [24,25]. Mutations in common to the *L*. *major* Friedlin wild-type parent and synonymous mutations were excluded. Genes mutated in recurrent fashion among mutants were identified and plotted for representing the genome-wide distribution of SNPs among the *L*. *major* Friedlin mutants. Copy number variations analysis was performed by comparing reads coverage per non-overlapping 5 kb genomic bins (normalized for total read counts) between the wild-type and the mutants for each chromosome.

### DNA constructs and transfection

The wild-type and mutated versions of the genes of interest were amplified from *L*. *major* Friedlin genomic DNA using primers pairs described in S1 Table and cloned into the pSP72α-puro-α expression vector [9]. The integrity of each insert was confirmed by Sanger sequencing. For episomal expression, 10 μg of DNA from the empty vector or the vector carrying the gene of interest were transfected by electroporation into *L*. *major* Friedlin promastigotes grown to the logarithmic phase using a Gene Pulser Xcell Electroporation System (Biorad) at 450 V, 500 μF, 2 mm and time constant range between 4 and 6 ms. Transfected cells were selected with 100 μg/ml of puromycin. Oligomers containing 60 bp extensions homologous to the 5′ or 3′ UTRs of the target genes followed by 20 bp of sequence homologous to the marker gene (PURO or NEO) were used to generate knockout cassettes. Ten μg of knockout cassettes were electroporated into *L*. *major* Friedlin promastigotes as described above and transfected cells were selected with 100 μg/ml puromycin or 40 μg/ml G418.

### Southern blot

Genomic DNA was extracted by phenol/chloroform as recommended by the manufacturer (Invitrogen) and digested with restriction enzymes (NEB). Probes covering the 3’ UTR of the target genes were amplified from *L*. *major* Friedlin genomic DNA using primers described in S1 Table. Southern blot hybridizations were performed with [α-^32^ P]dCTP-labeled DNA probes according to standard protocols [26].

### CRISPR-Cas9 based genome editing

Our CRISPR-Cas9 based genome editing was carried out essentially as described previously [27]. A plasmid containing the CRISPR associated protein 9 (Cas9) nuclease, pLPhygCAS9 [28], was transfected into *L*. *major* Friedlin logarithmic phase promastigotes. Transfected cells were selected with 300 μg/ml of hygromycin. Four μl of 100 μM guide RNAs (gRNAs) targeting *FT1*, *DHFR-TS* or *PTR1* (all listed in S1 Table) annealed with 4 μl of a 100 μM tracrRNA were co-transfected along with 5 μg of a 200 bp repair cassette covering the mutation of interest (*FT1* ^G116R^, *FT1* ^G129D^, *FT1* ^A430V^, *FT1* ^P555S^, *DHFR-TS* ^T107I^, *DHFR-TS* ^E291K^, *PTR1* ^A28T^ or *PTR1* ^S253F^) in the *L*. *major* Friedlin line harboring the pLPhygCAS9 episome using the U-014 program of an Amaxa Nucleofector (Lonza). Repair cassettes were prepared by PCR amplification using primers listed in S1 Table using gDNAs extracted from the appropriate mutant as a template. PCR fragments were cloned into the pGEM-T Easy (Promega) subcloning vector. The integrity of each insert and the presence of the mutation of interest were confirmed by Sanger sequencing. Five μg of repair cassette for transfection were produced by amplification of the cloned fragment using the same PCR primers. The integrity of the fragment was confirmed again by Sanger sequencing prior to the transfection. Upon their insertion in the genome, the mutations of interest either destroyed the protospacer adjacent motif (PAM) of the co-transfected gRNA or inserted in its vicinity, hence preventing Cas9 to cut the recombined allele. After transfection, cells were incubated for 48h at 25°C with shaking. Transfected cells were then cloned on SDM agar plates and individual clones expanded in M199 complete medium. The presence of the mutations in the genes of interest was confirmed by PCR amplification followed by Sanger sequencing. To rule out off-targets in other known MTX resistance genes we sequenced the genes *FT1*, *DHFR-TS*, *PTR1*, LmjF.17.1130 and LmjF.17.1360 in each of our edited clones. Two clones were excluded as they underwent complex rearrangements at the level of *FT1* upon attempts to edit this gene. All other edited clones reported here had no unwanted mutations in any of the five genes tested.

### Structure modelisation

Structure files were downloaded from Protein Data Bank (*T*. *cruzi* DHFR-TS, *L*. *major* PTR1) or AlphaFold (*L*. *major* DHFR-TS) and structure modelisations were realized on PyMOL Molecular Graphics System version 2.5.2.

### Real-time RT-PCR

RNAs were isolated from cells in early-logarithmic phase using the RNeasy Mini Kit Plus (Qiagen), according to the manufacturer’s instructions. The purity and integrity of RNAs were measured on an Agilent 2100 bioanalyzer (Agilent Technologies). cDNAs were synthesized from 1μg of RNA using the Superscript II reverse transcriptase enzyme and Oligo(dT)12-18 primers (Invitrogen) according to the manufacturer’s instructions. Real-time RT-PCR was performed in biological triplicates, each with three technical replicates, for the *PTR1* and *DHFR* genes and the endogenous control gene β-tubulin using SYBR green (Invitrogen) and PCR primers listed in S1 Table. Reactions without reverse transcriptase were included to control for genomic DNA contamination. Real time RT-PCR was performed in a Rotor Gene-3000 (Corbett Research) as follows: 4 min at 95°C followed by 40 cycles of 20 s at 94°C, 20 s at 60°C, 64°C or 66°C for the *β-tubulin*, *DHFR-TS* and *PTR1* genes respectively, and then 20 s at 72°C. Gene expression levels of the target genes were normalized to the endogenous control *β-tubulin*.

### Statistical analysis

The statistical analyses were performed with the GraphPad Prism 5.1 software using two-tailed unpaired t-test.

## Results

### Generation of *Leishmania* mutants resistant to MTX by chemical mutagenesis

**Fig 1:**
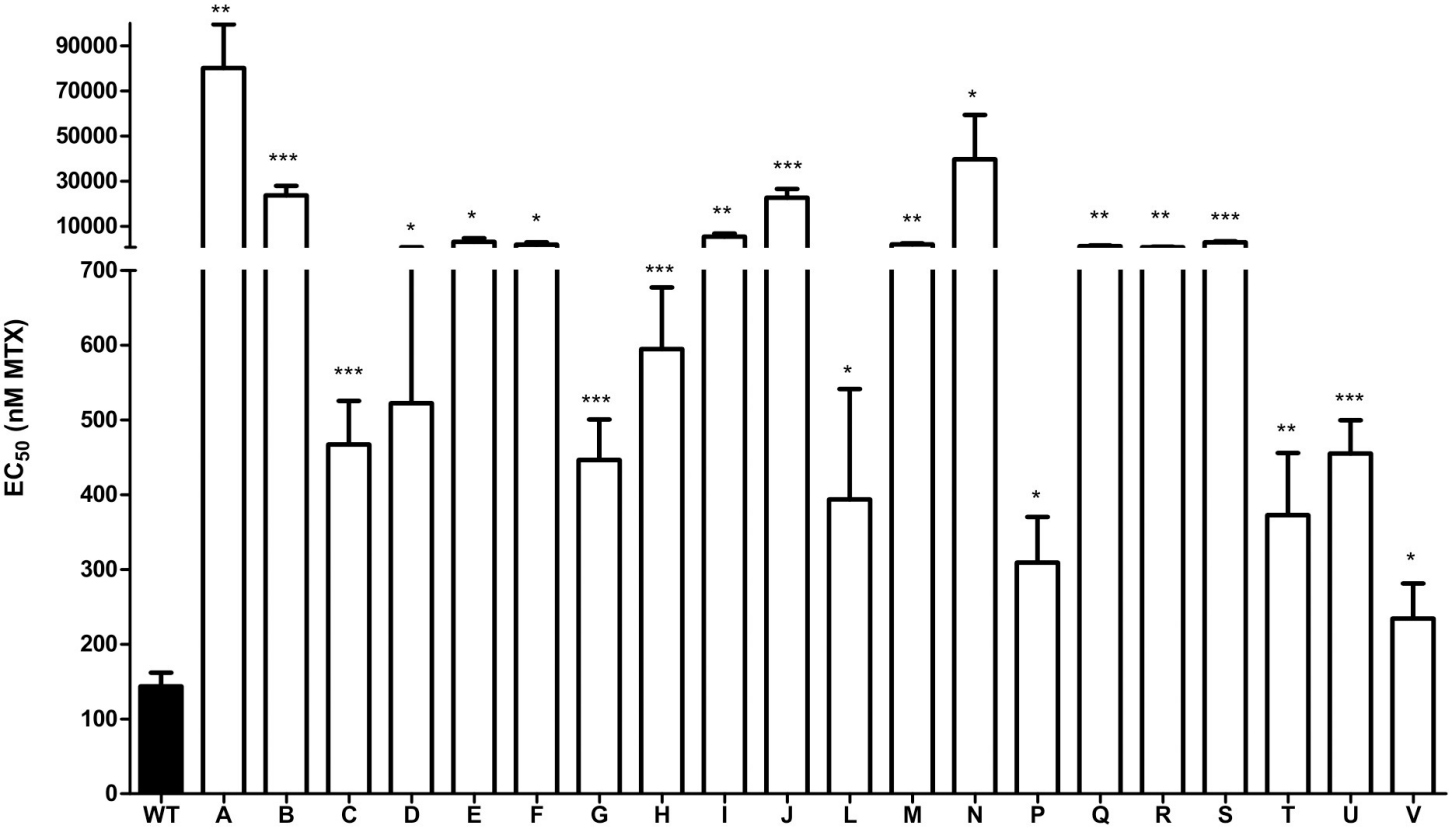
Methotrexate susceptibility of *L*. *major* Friedlin mutants derived from a Mut-seq screen. Susceptibility assays to MTX were performed on clonal *L*. *major* Friedlin mutants in M-199 medium. Mutants were 2- to 400-fold more resistant to MTX in comparison to wild-type (black). Data are shown as means ± SD of three independent biological replicates for each mutant. The EC_50_ of the mutants were compared to the wild-type using unpaired two-tailed t-test. *, P<0.05; **, P<0.01; ***, P<0.001.

**Fig 2:**
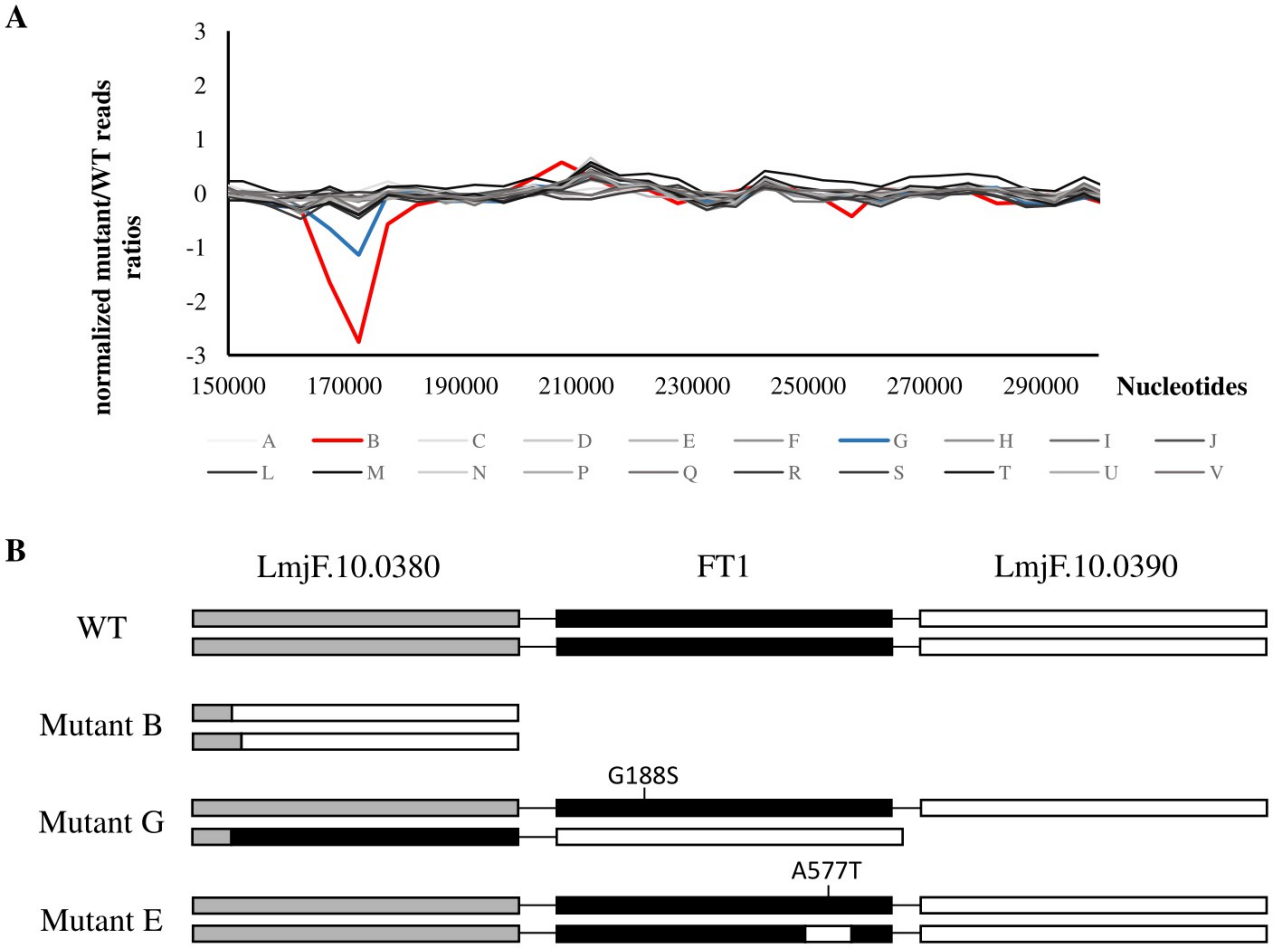
Gene deletion on chromosome 10. A) The normalized mutant/wild-type reads ratios per non-overlapping 5kb bins along chromosome 10 show a gene deletion signature for mutants B and G in the vicinity of position 172,500 characterized by a drop in reads ratios. B) The deleted region corresponds to a cluster of folate-biopterin transporter family genes containing LmjF.10.0380, LmjF.10.0385 (*FT1*) and LmjF.10.0390. Gene rearrangements detected by NGS were confirmed by PCR and Sanger sequencing and involved homologous recombination, gene conversion and point mutations.

A clonal population of *L*. *major* Friedlin was mutagenized with EMS and selected on plates containing MTX at either 5- or 20-fold its MTX EC_50_. Clones were picked and twenty selected clones were resistant to MTX, with a 2- to 400-fold decrease in MTX susceptibility in comparison to wild-type cells (Fig 1). The genome of these 20 mutants were sequenced at a coverage ranging from 30- to 70-fold. Reads depth coverage over the 36 chromosomes was used to predict ploidy and copy number variations (CNVs). As previously observed in an unrelated Mut-seq screen in *Leishmania* [20], some cases of supernumerary chromosomes correlating with the resistance phenotype were found (chromosomes 5 and 28) (S1 Fig), while chromosome 17 was haploid in most mutants (S1 Fig). The analysis for CNVs revealed a 5kb deletion on chromosome 10 in mutants B (diploid deletion) and G (haploid deletion) (Fig 2A). This region encodes the folate transporter FT1 [8,29]. *FT1* is within a locus of 7 Folate Biopterin Transporter (FBT) genes on chromosome 10 [4] and the diploid deletion in mutant B was created by homologous recombination between FBT paralogs flanking *FT1* (Fig 2B). The recombination events differed slightly for the two alleles in mutant B (S2 Fig). A different gene rearrangement occurred in mutant G in which the recombination took place between conserved regions shared by *FT1* and the upstream FBT gene LmjF.10.0380 (Fig 2B). The precise recombination points were confirmed by Sanger sequencing (S2 Fig).

As expected from the nature of the screen, mutations were observed throughout the genome of the mutants, with some regions showing a higher frequency of mutations (S3 Fig and S3 Table). By focusing on coding mutations, we searched for genes mutated in at least two independent mutants but excluding genes with the exact same change in all mutants, as previous experience has shown that these usually correspond to natural polymorphisms (after Sanger sequencing of that region in wild-type cells) rather than mutations induced by EMS and selected with MTX. From the genes with the highest mutation density (Table 1), the folate transporter (*FT1*) [8,29] and the dihydrofolate reductase thymidylate synthase (*DHFR-TS*) genes had the most mutations (Table 1). Interestingly, several other genes known to be involved in folate metabolism also showed a high diversity of mutations (Table 1).

**Table 1:** Mutations in genes of *L*. *major* Friedlin following a Mut-Seq screen with methotrexate.

Gene ID	Function	Mutant	Mutations^a^	
			DNA	Protein^b^
LmjF.06.0860	*DHFR-TS*	C	C706T	Q236S
		E	C346T	P116S
		H	G328A	E110K
		I	G860A	R287H
		L	C5T	S2F
		M	G871A	E291K
		P	C317T	A106V
		R	C587T	P196L
		T	C64T	P22S
		U	C320T	T107I
		V	G40A	E14K
LmjF.07.0090	methionine synthase	G	C3499T	P1167S
		J	C3490T	P1164S
		U	G1529A	R510H
LmjF.10.0380	folate biopterin transporter	A	G184A	G62S
		B	**Gene rearrangement**	
		G	C298T, A301C	P100S, I101L
		J	C131T	P44L
LmjF.10.0385	*FT1*	A	G1617A, C1813T	W539*, Q605*
		D	G531A, G1168A	W177*, A390T
		E	G1729A, C1740G, C1741A, C1743T, C1746T, G1751A, A1752G, G1755C, C1776G, C1780A, T1782C, C1783A	A577T, R581S, R584Q, L594I, L595M
		F	G413A, C1442T	R138H, T181I
		G	G562A	G188S
		J	G346A, C1663T	G116R, P555S
		N	G386A, C1289T	G129D, A430V
		P	C1360T, C1325T	Q459*, A542V
		Q	G607T, C610A, G1343A	A203S, L204M, G448D
		R	C593T	P198L
		S	C392T, G1699A, G2038A	A131V, G567S, E680K
		T	G633A, G1588A	W211*, G530S
LmjF.10.0400	*FT5*	C	G2059A	A687T
		L	C947T	A316V
LmjF.17.0630	hypothetical protein	D	**C671T**	**R224H**
		Q	**C124T**	**G42R**
LmjF.17.1130	NOL1/NOP2/sun family protein	A	**G487A**	**E163K**
		B	**G2654A**	**G885D**
		E	**G2983A**	**A995T**
		F	**G1666A**	**A556T**
LmjF.17.1360	L-galactonolactone oxidase (*ALO*)	D	**C875T**	**A292V**
		L	C1504T	P502S
		N	**C775T**	**P259S**
LmjF.19.0920	folate biopterin transporter	V	C173T	A58V
LmjF.21.1210	thymidine kinase (*TK*)	I	C761T	R254Q
		L	C793T	E265K
LmjF.23.0270	*PTR1*	B	G82A	A28T
		T	C758T	S253F
LmjF.28.2370	serine hydroxyl-methyltransferase (*SHMT-L*)	C	C376T	P126S
		D	G430A	G144S
		P	C304T	R103W
LmjF.31.0010	5-methyltetrahydro-pteroyltriglutamate-homocysteine S-methyltransferase	I	C877T	G293R
		M	G181A	L61F
		R	C542T, C713T	G181D, G238D
		S	C1159T	A387T
		V	C544T	D182N
LmjF.34.2500	protein phosphatase	E	C565T	V189M
		M	C1097T	G366D
LmjF.34.2510	protein phosphatase	B	G1046A	A349V
		F	C1315T	A439T
LmjF.36.0510	mitochondrial carrier protein	C	C557T	R186H
		F	G2234A	S745L
		H	C1042T	D348N
		J	C1799T	R600H
		L	C641T	R214H
		T	C973T	A325T
		U	C2110T	S707N
LmjF.36.2610	folylpolyglutamate synthetase (*FPGS*)	F	C1335T	M445I
		M	G1694A	P565L

^**a**^ Mutations in bold are homozygous, either because their chromosome is haploid in the mutant (LmjF.17) or because the mutation is found on both alleles of a diploid chromosome (gene rearrangement for LmjF.10.0380 in mutant B). Other mutations were found on a single allele of diploid or supernumerary chromosomes. See S1 Fig. for chromosomal ploidy of the mutants.

^b^ Asterisks (*) denote stop codons.

### Mutations in folate transporter genes

**Fig 3:**
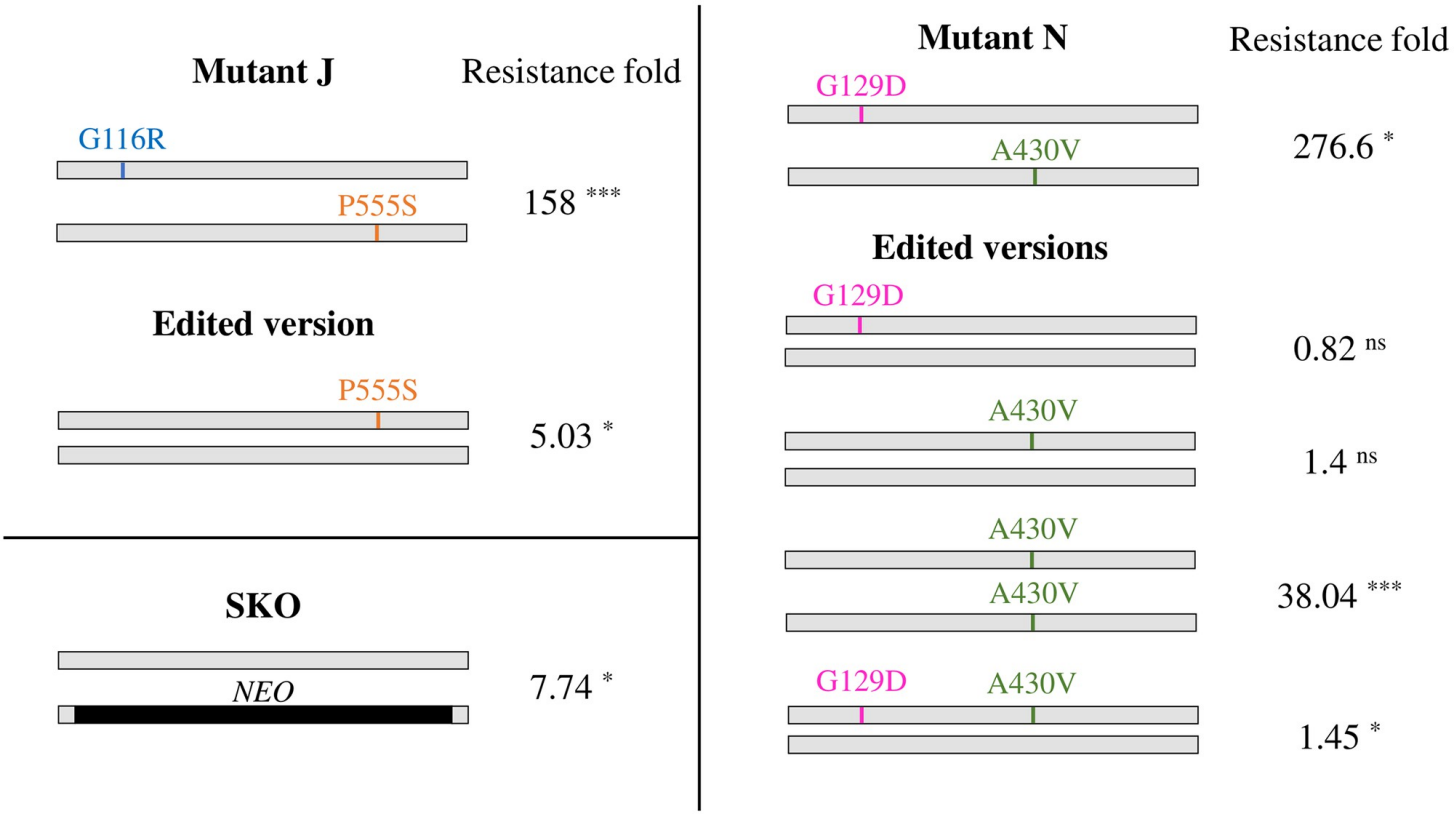
Selected mutations in the folate transporter *FT1* and their analysis by DNA editing. *FT1* mutations from mutants J and N were introduced in *L*. *major* Friedlin wild-type by genome editing. The EC_50_ to MTX of the transformants, of the parent mutants and of *L*. *major* Friedlin wild-type were determined from three independent biological replicates. A *L*. *major* strains with one allele of *FT1* replaced by the NEO selection marker (SKO) was also included for comparison. The significance of the fold increase in MTX resistance of the edited cells (or of the original mutants) compared to the wild-type, referred in the figure as resistance fold, was tested using unpaired two-tailed t-test. *, P<0.05; ***, P<0.001; ns, not significant.

Four members of the FBT family, LmjF.10.0380, LmjF.10.0385 (*FT1*), LmjF.10.0400 (*FT5*) and LmjF.19.0920 were mutated in the Mut-seq screen (Table 1). In addition to the rearrangements at the *FT1* locus described above for mutants B and G, a gene conversion event was detected in mutant E where a small part of the LmjF.10.0390 FBT gene was inserted into one allele of *FT1* (Fig 2B), leading to a stretch of SNPs at the 3’ end of the gene (S2 Fig.). Heterozygous mutations in *FT1* were observed in 11 additional mutants, including in the non-recombined alleles of mutants E and G (Fig 2B and Table 1). Interestingly, more than one *FT1* heterozygous mutations were observed for a majority of mutants (Table 1), making it possible that the two *FT1* alleles within individual mutants acquired different mutations. This was indeed experimentally validated for mutants J and N where each allele had a specific mutation (Fig 3). No mutations in *FT1* have been previously reported in MTX selected cells. We therefore tested, using CRISPR-Cas9 mediated gene editing, the function of key *FT1* mutations in MTX resistance. We focused on mutations observed in mutants J and N because we validated that each allele was independently mutated (Fig 3) and that both of these mutants were highly resistant to MTX (Fig 1) with no mutation in *DHFR-TS* (Table 1). The mutations G116R and P555S of mutant J and the mutations G129D and A430V of mutant N occurred at residues conserved among *Leishmania* FT1 orthologues (S4 Fig). The mutations were spread throughout the predicted secondary structure of FT1 (S5 Fig). The introduction in one allele of *L*. *major* wild-type of the *FT1* mutation leading to the P555S substitution from mutant J resulted in parasites 5-fold more resistant to MTX (Fig 3). This level of resistance is similar to a *L*. *major FT1* single knock out strain (S6A and S6B Fig) that is 7.7-fold more resistant to MTX than the wild-type (Fig 3 and S6C Fig). Our gene editing attempts with the G116R mutation (from mutant J) were less rewarding as bystander mutations were spuriously introduced in *FT1* along with the target mutation (S7 Fig). Similarly, it was challenging to introduce the G129D and A430V mutations found in mutant N into a wild-type strain. While we could not obtain a parasite with G129D and A430V on different alleles of the same cell, as found in mutant N, we could obtain parasites with the two mutations on a single allele, or parasites with either a single G129D or A430V mutation in independent transformants. None of the heterozygous transformants were resistant to MTX (Fig 3 for G129D and A430V from mutant N and S7 Fig for G116R from mutant J with bystander mutations). This further supports the difficulty in carrying DNA editing of highly related multigene families [30]. Of note however, parasites having both alleles carrying the A430V mutation (S4 Table) from mutant N were highly resistant to MTX (Fig 3). These results suggest that mutations on both *FT1* alleles are required to obtain high levels of MTX resistance, either by gene deletion/rearrangement or by SNPs.

Three other members of the FBT family were mutated (Table 1). We have no evidence for the role of LmjF.10.0380 and LmjF.19.0920 in folate transport [4] but LmjF.10.0400 (*FT5*) was shown to be a high affinity low capacity folate transporter [31]. Heterozygous mutations were found in the *FT5* of mutants L (A316V) and C (A687T) (Table 1), two mutants weakly resistant (3-fold) to MTX (Fig 1). *DHFR-TS* is also mutated in both of these mutants (Table 1) and could potentially be the main contributor to their resistance. Before embarking in a complex gene editing protocol of repeated genes, we generated a single knockout of *FT5* in *L*. *major* and shown that its susceptibility to MTX was unchanged (S8 Fig), rendering unlikely that the mutations observed in *FT5* contribute importantly to the MTX phenotype.

### *DHFR-TS* and *PTR1*

**Fig 4:**
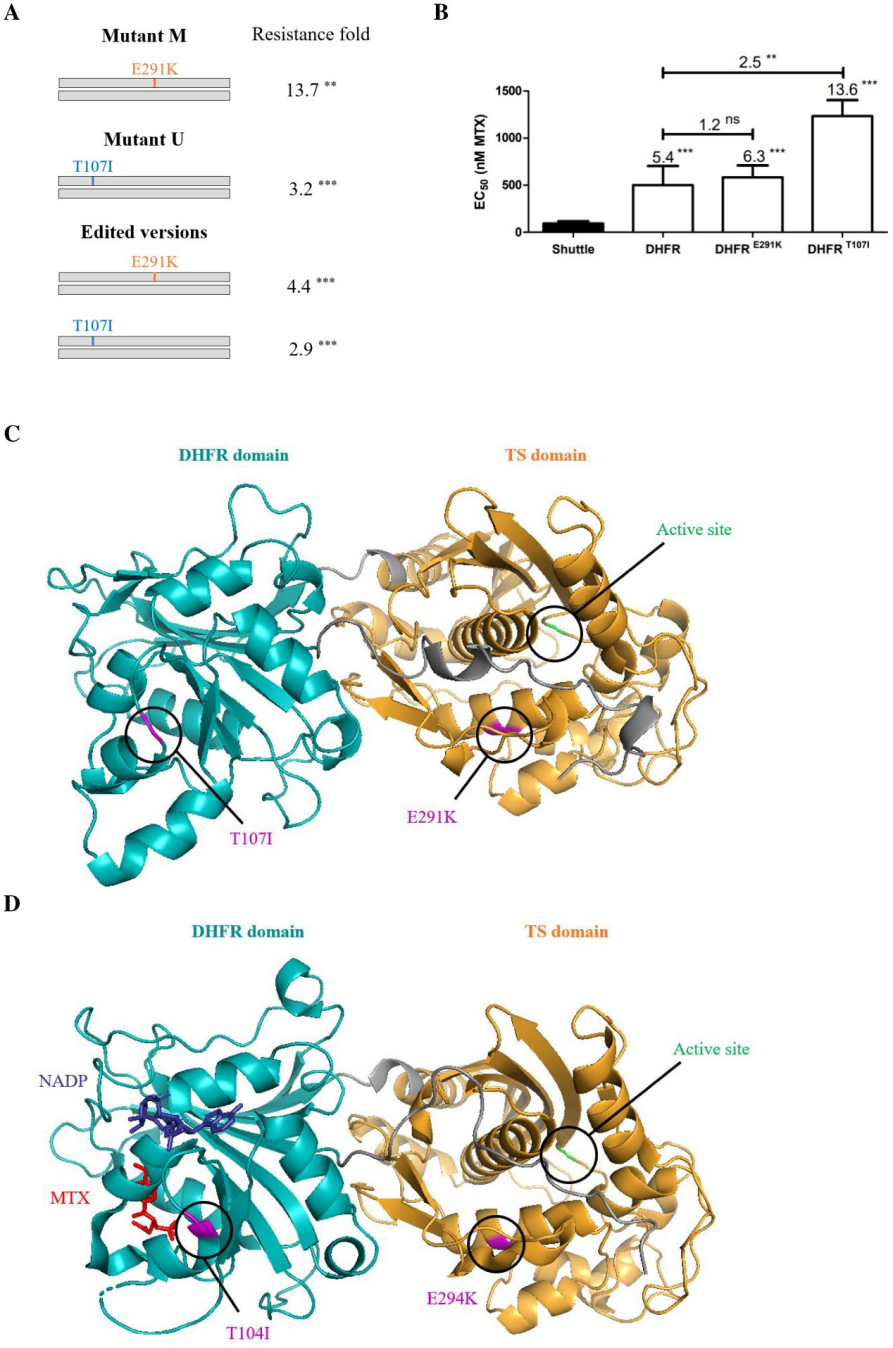
Mutations in *DHFR-TS* and their role in methotrexate resistance. A) *DHFR-TS* mutations from mutants M and U were introduced in *L*. *major* Friedlin wild-type by genome editing. The EC_50_ to MTX of the transformants, of the parent mutants and of *L*. *major* Friedlin wild-type were determined from three independent biological replicates. The significance of the fold increase in MTX resistance of the edited cells (or of the original mutants) compared to the wild-type, referred in the figure as resistance fold, was tested using unpaired two-tailed t-test. B) EC_50s_ to MTX conferred by episomal overexpression of the wild-type or mutated versions of *DHFR-TS* in *L*. *major* Friedlin wild-type. The fold increase in resistance to MTX between transfectants or compared to the wild-type is indicated above the boxes. The significance of the fold increase in resistance was evaluated using unpaired two-tailed t-test. **, P<0.01; ***, P<0.001; ns, not significant. The predicted structures of DHFR-TS of *L*. *major* (C) and *Trypanosoma cruzi* (D) contain the DHFR (blue) and TS domains (orange). Mutations investigated are highlighted in pink, the active site in light green, the cofactor in dark blue and MTX in red. Residues T107 and E291 in *L*. *major* DHFR-TS respectively correspond to T104 and E294 in the DHFR-TS of *T*. *cruzi*.

Next to *FT1*, the second gene with the most diverse set of mutations was *DHFR-TS*, the main target of antifolates [3,32]. Resistance to MTX mediated by *DHFR-TS* is usually due to gene amplification although in one report a point mutation was linked to MTX selection [33]. Heterozygous mutations in *DHFR-TS* were found in eleven independent mutants (Table 1) and all were confirmed by Sanger sequencing. All mutations, except E14K, were in amino acids conserved throughout various species (S9 Fig). We investigated by CRISPR-Cas9 mediated gene editing the role of DHFR-TS mutations found in mutants M (E291K) and U (T107I), respectively 13.7- and 3.2-fold resistant to MTX (Fig 1). We focused on these two mutants because they do not have mutations in any FBTs. Introduction in *L*. *major* wild-type of the *DHFR-TS* mutations leading to the E291K and T107I substitutions (S4 Table) led to parasites 4.4-fold and 2.9-fold more resistant to MTX than the control cells, respectively (Fig 4A). From this result, we tested whether the mutated *DHFR-TS* could be used as dominant positive selection marker. For this, we made episomal constructs for the overexpression of DHFR-TS^WT^, DHFR-TS^E291K^ or DHFR-TS^T107I^ and the latter was found to confer 2.5-fold more resistance to MTX than the overexpressed wild-type DHFR-TS (Fig 4B). To exclude that this phenotype was due to a differential expression of *DHFR-TS* in the three transfectants, we carried out RT-qPCR and found no significant difference in expression between the three transfectants (S10A Fig). The *L*. *major* DHFR-TS structure is available on Alphafold (Uniprot P07382) and using the Pymol software (version 2.5.2) [34] we modeled the impact of the two mutations on the DHFR-TS structure. The mutation T107I is in the DHFR domain (Fig 4C) while the E291K mutation is near the active site of the TS domain (Fig 4C). Since the *Trypanosoma cruzi* crystal structure of DHFR-TS complexed with MTX is known [35], we modeled the two mutations and found that the T107I substitution is near the MTX and co-factor binding sites (Fig 4D).

**Fig 5:**
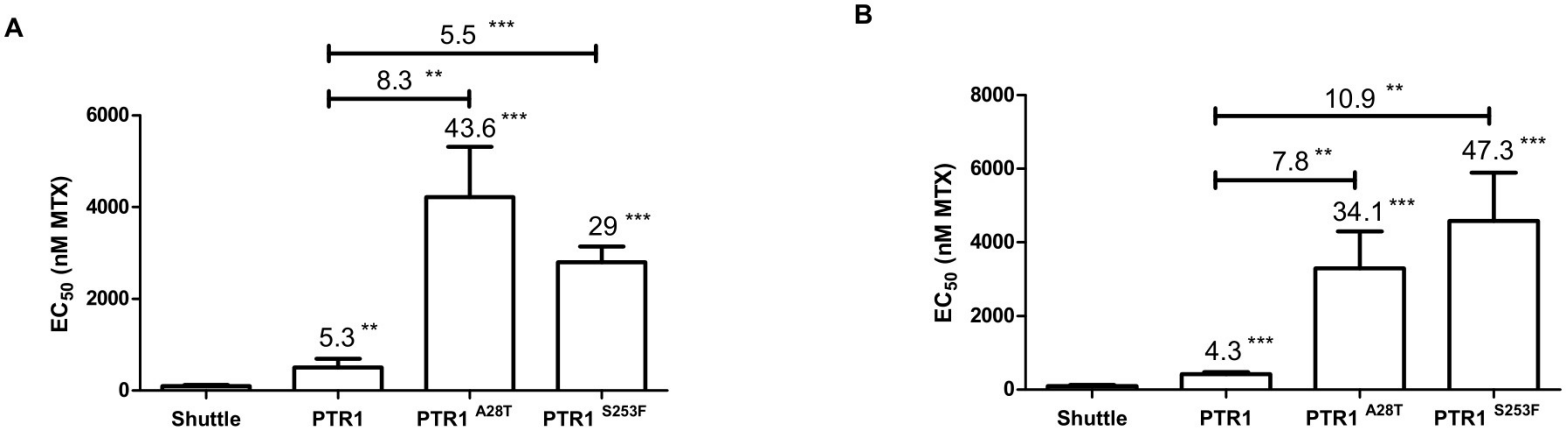
Dominant positive effect of mutated versions of *PTR1*. EC_50s_ to MTX conferred by the overexpression of wild-type or mutated versions of *PTR1* in *L*. *major* Friedlin wild-type compared together and to the mock transfectants. A and B shows the data from two independent transfections. Resistance fold is indicated on top of each comparison. Data are shown as means ± SD of at least three biological independent replicates for each mutant. The significance of the difference in susceptibility to MTX was evaluated using unpaired two-tailed t-test. **, P<0.01; ***, P<0.001.

The enzyme PTR1 was first isolated because its gene is frequently amplified in MTX resistant cells [9,10]. Heterozygous mutations in *PTR1* were found in only two independent mutants (Table 1). The mutations leading to amino acids substitutions A28T in mutant B and S253F in mutant T were confirmed by Sanger sequencing. We succeeded in introducing the two mutations in *L*. *major* wild-type by DNA editing but surprisingly these parasites were not more resistant to MTX (S11 Fig). Of note however, episomal overexpression in *L*. *major* Friedlin of the mutated versions produced more resistance to MTX than overexpression of wild-type *PTR1* (Fig 5). To exclude that this phenotype was due to a differential expression, we carried out RT-qPCR and found no significant difference in *PTR1* expression between the three transfectants (S10B Fig). Many crystal structures of PTR1 were elucidated [36–38]. Neither A28T nor S253F were close to the active site, although S253F was found close to the co-factor binding site (S11 Fig).

### Mutations in other genes

We have proven that point mutations in *FT1*, *DHFR-TS* and *PTR1* are contributing to MTX resistance. There were several other candidate genes mutated in at least two independent mutants, and it is noteworthy that several of these gene products are involved in folate metabolism (Table 1). In most cases the mutations were heterozygous with one allele mutated and one wild-type allele remaining (Table 1). This includes cobalamine-dependent (LmjF.07.0090) and -independent (LmjF.31.0010) methionine synthases, the folylpolyglutamate synthetase (LmjF.36.2610), a thymidine kinase (LmjF.21.1210) involved in MTX response [16], the mitochondrial serine hydroxymethyl-transferase (LmjF.28.2370) [13] and two protein phosphatase (LmjF.34.2500 and LmjF.34.2510) whose overexpression produced MTX resistance [17]. While folate related enzymes represent 0.14% of the open reading frames of *Leishmania*, 82.8% of those genes were found to be mutated, against 62.8% of all genes in the genome being mutated, an enrichment that is statistically significant (p< 0.05).

Overall, 2993 genes were found to be mutated in at least two mutants (S3 Table), a daunting number for prioritizing the functional characterization of mutations. In addition of being mutated in at least two independent mutants, we have established criteria for prioritizing targets [20]. We focused on homozygous mutations since these are usually more likely to be phenotypic, on genes with mutations that differ between the mutants, on genes smaller than 6kb and finally on the potential relevance of the gene in view of existing knowledge of drug activity. While applying these criteria, we selected three genes on chromosome 17. Chromosome 17 is haploid in most mutants (S1 Fig) and thus all of its mutations are homozygous. The three genes, LmjF.17.0630, LmjF.17.1130, LmjF.17.1360 encodes respectively a hypothetical protein, a S-adenosylmethionine (SAM)-dependent methyltransferase part of the NOL1/NOP2/sun family (NSUN) protein, and a L-galactonolactone oxidase (*ALO*). Instead of using gene editing we transfected the wild-type (and mutated) versions of each of these genes in the appropriate mutants (i.e. the mutant in which the studied genes were mutated). No phenotype was observed when the LmjF.17.0630 wild-type gene was transfected into mutant D (Table 2) but transfection of the wild-type versions of either LmjF.17.1130 or LmjF.17.1360 (but not their mutated counterparts) in mutants F and N, respectively, significantly re-sensitized cells to MTX (Table 2).

**Table 2:** Contribution to MTX resistance of the mutations detected in genes unrelated to folate metabolism and highlighted by the Mut-seq screen.

Mutants	Gene^a^	EC_50_ (nM)^b^
D	None	588.7 ± 290.1
	LmjF.17.0630	397.3 ± 94 (1.5)
F	None	5 835 ± 2 878
	LmjF.17.1130	1 976 ± 1 420 (3*)
	LmjF.17.1130^A556T^	7 000 ± 2 628.7 (0.8)
N	None	34 066.7 ± 5 320^c^
	LmjF.17.1360	19 700 ± 5 306.9 (1.7***)^c^
	LmjF.17.1360^P259S^	28 166.7 ± 4 354.7 (1.2)

^**a**^ Genes overexpressed in the mutants from the pSP72α-puro-α expression. For mutated versions, the amino acid substitution conferred by the mutation is indicated in superscript. None, empty vector; LmjF.17.0630, hypothetical protein; LmjF.17.1130, S-adenosylmethionine (SAM)-dependent methyltransferase part of the NOL1/NOP2/sun family; LmjF.17.1360, L-galactonolactone oxidase.

^**b**^ Results are shown as the mean ± SD of at least three biological replicates. The fold decrease in MTX resistance compared to the mock control is indicated within parentheses. *, P ≤ 0.05; ***, P<0.001 by unpaired two-tailed t-test.

^**c**^ Mean of 2 independent transfections.

## Discussion

Historically, MTX resistance studies in *Leishmania* were mostly associated with CNVs: amplification of *PTR1* or *DHFR-TS*, or deletion of *FT1* (reviewed in [2,3]). The screen presented here highlighted that point mutations in key genes can also be selected upon MTX selection and can contribute to the resistance phenotype. Indeed, our chemogenomic screen, in cells grown in M199, has revealed a plethora of new mutations involved in antifolate resistance and some of those are likely to play a role in folate metabolism. We elected to perform our screen in M199 medium with lower folate concentration than for example SDM-79 medium since MTX response in *Leishmania* is highly dependent on folate concentrations [39–41]. Carrying our current chemogenomic screen with a range of folate concentrations may have led to a different set of mutations. Indeed, a Cos-seq screen with MTX in *Leishmania* carried either in M199 or SDM-79 led to different candidate genes [18]. Under the conditions used here, our screen highlighted the central role of FT1 in response to MTX challenge. Chemical mutagenesis will usually select for point mutations but in at least three mutants (B, E, and G) we observed gene rearrangements deleting *FT1* or a combination of gene rearrangement (deletion or gene conversion) with point mutations (Fig 2). As no MTX resistant colonies were recovered from the non-mutagenized control, one possibility is that gene rearrangements were helped by the presence of bystander mutations induced by the chemical mutagenesis, such as mutations in a DNA repair gene.

Several MTX resistant mutants had point mutations in *FT1* (Table 1). It is salient to mention that mutations in *FT1* upon MTX selection have never been described, although site directed mutagenesis of conserved residues in FT1 showed they contribute to MTX resistance [42]. Interestingly, the residue equivalent to R138 (mutated in mutant F) in *L*. *infantum* (R134) was proven by site directed mutagenesis to contribute to MTX resistance and reduced folate/MTX uptake [42]. By gene editing we have proven the role of the FT1 P555S substitution in MTX resistance (Fig 3). This mutation seems to inactivate one copy of *FT1* since the resistance level in these edited cells is similar to a cell where one copy of the gene was inactivated (S6 Fig). It also appears from our data that both alleles of *FT1* need to be mutated to achieve high level of resistance to MTX. Because of the repeated nature of the FBT gene family it has been challenging, however, to replicate by gene editing the exact mutations found in the mutants. Challenges in editing repeated DNAs have been reported by others [30].

A frequent mechanism of MTX resistance in *Leishmania* is amplification of the *DHFR-TS* or *PTR1* genes [5–7]. While mutations in DHFR-TS are common in malaria parasites selected for antifolate resistance [43], this is not the case with *Leishmania*. There was a single example and this corresponded to a gene that was first amplified before being mutated (M53R) upon additional MTX selection [33]. Now we describe point mutations in the *DHFR-TS* chromosomal copy, and show that editing *DHFR-TS* to code for T107I or E291K produces resistance (Fig 4A). This is likely to be a gain of function, as inactivation of one copy of *DHFR-TS* was shown to produce MTX susceptibility [18]. Resistance levels in these edited cells are similar but not identical to what found in mutants U or M (Fig 4) suggesting that other mutations may also contribute to resistance in these mutants. Other mutations related to folate metabolism were noted in these mutants (Table 1), some of which like the protein phosphatase LmjF.34.2500 and the folylpolyglutamate synthase LmjF.36.2610 have already been proven to modulate MTX susceptibility in *Leishmania* [11,17]. Interestingly, overexpression of DHFR-TS^T107I^, a version of the protein altered at an amino acid close to the MTX binding site (Fig 4C), produced more resistance than the overexpressed wild-type protein (Fig 4B), a situation also reported for the DHFR-TS^M53R^ version [33]. One can speculate that the T107I mutation may either modify the affinity of the protein for either folate or MTX or modulate its stability. Site directed mutagenesis of PTR1 revealed key amino acids involved in its activity [44] but no mutation in PTR1 has ever been observed in MTX resistant parasites. Our Mut-seq screen revealed two such mutations and while their introduction in one of the chromosomal copy of *PTR1* was not sufficient for producing resistance (S11 Fig), the episomal overexpression of either the A28T or S263F variants of PTR1 produced 6–10 times more resistance than overexpression of the wild-type version (Fig 5). Chromosome 23 is triploid in our strains (S1 Fig) and it is possible that one mutated allele out of three is not sufficient by itself (e.g. presence of other phenotypic mutations in the mutant but not in the edited cells) while the multiple copies from the mutated episomal construct leads to increased MTX resistance. A paucity of selectable markers exists for reverse genetics in *Leishmania* and these mutated versions of *PTR1* could possibly be helpful as an additional selectable marker.

Several genes directly involved in folate metabolism were mutated in more than one mutant (Table 1), providing a long list of potential MTX resistance contributors. We also observed a plethora of genes with heterozygous mutations in at least two independent mutants (S3 Table), many of which have been associated with drug response such as trypanothione reductase (LmjF.05.0350), lanosterol 14-alpha demethylase (LmjF.11.1100), ornithine decarboxylase (LmjF.12.0280) and many others (S3 Table), and further work is needed to assess whether any of these have a role to play in MTX response. Based on previous experience with Mut-seq [20] we focused on homozygous mutations, several of which were on genes located on the haploid chromosome 17 (S1 Fig). An episomal transfection of the wild-type version of two of those genes, the SAM-dependent methyltransferase LmjF.17.1130 part of the NSUN protein family and the L-galactonolactone oxidase LmjF.17.1360, resensitized mutants to MTX. NSUN methylates cytosines in RNA to produce 5-methylcytosine which have multiple functions (reviewed in [45]) but additional work is needed to understand how this contributes to MTX resistance. S-adenosyl methionine has already been associated with modulation of antifolate activity in *Leishmania* [12] and possibly this may have a link with this NSUN SAM-dependent methyltransferase. L-galactonolactone oxidase catalyzes a reaction leading to the synthesis of ascorbic acid, a molecule proposed to serve in mitigating oxidative stress in *Leishmania* [46] and indeed an *ALO* knockout strain of *Leishmania* was found to be more susceptible to oxidative stress [47]. Additional work may suggest how ALO protects against MTX challenge. Our transfection data support the hypothesis that the mutation in the latter two genes are loss of function mutations as adding back a wild-type allele (but not a mutated one) restores part of the sensitivity phenotype.

In summary, our Mut-seq screen has highlighted many of the genes involved in folate metabolism in *Leishmania*. Our gene editing work has shown that chromosomal point mutations in *DHFR-TS* or *FT1* produce MTX resistance. Mutated PTR1, when overexpressed, as the potential to serve as a dominant selectable marker much needed for reverse genetics work in *Leishmania*. A long list of other potential candidates involved in folate metabolism (Table 1) or in other functions (S3 Table) have been highlighted and may play a role in response to a MTX challenge. By gene overexpression we have highlighted the role of two new genes associated with MTX resistance. While Mut-seq leads to many mutations, focusing on the most recurrent mutations in independent mutants helps in prioritizing the most likely candidates. Mut-seq is a useful screen that can complement *in vitro* resistance evolution and Cos-seq screens to reveal the mode of action and resistance mechanisms against novel drugs.

## Supporting information

S1 Fig Chromosome ploidy inferred from NGS coverage for *L*. *major* Friedlin wild-type and mutants resistant to MTX. (TIF)

S2 Fig DNA alignment of genes in the FBT transporters cluster. LmjF.10.0380 sequence is highlighted in blue, FT1 in green and LmjF.10.0390 in pink. SNPs are highlighted in grey.
(PDF)

S3 Fig Genome-wide distribution of SNPs in *L*. *major* Friedlin mutants selected for MTX resistance. Genes for the 36 chromosomes are shown as bars colored according to their mutation frequency among the MTX-resistant mutants. Gray bars denote non-mutated genes. White segments correspond to intergenic regions.
(TIF)

S4 Fig Protein alignment of FT1 from several *Leishmania* species. FT1 proteins of *L*. *major* Friedlin (LmjF.10.0385), *L*. *infantum* (LINF_100009300), *L*. *donovani* (LDHU3_10.0570), *L*. *mexicana* (LmxM.10.0370), *L*. *tropica* (LTRL590_000024200), *L*. *braziliensis* (LbrM.10.0400) and *L*. *panamensis* (LPMP_100340) were aligned. Homologous regions were highlighted in grey and amino acids found mutated in in the Mut-seq screen are shown in bold.
(TIF)

S5 Fig *L*. *major* Friedlin FT1 structure. FT1 has fourteen transmembrane domains. Amino acids are represented by lettered circles. Amino acids mutated in FT1 are colored according to the following scheme: yellow for mutations found in mutant J, pink for mutations found in mutant N and blue for mutations found in mutants from our Mut-Seq screen that were not further studied.
(TIF)

S6 Fig *L*. *major* Friedlin *FT1* single knockout. A) Schematic representation of the FT1 gene (top) and inactivation cassette with the neomycin selection marker (NEO, bottom). The position of the primers (a and b) used for the amplification of the probe for Southern Blot hybridization is depicted by arrowheads. B) Southern blot of genomic DNA derived from *L*. *major* Friedlin wild-type and FT1 single KO digested with HindIII and hybridized with a FT1 3′UTR probe. Lane 1, *L*. *major* Friedlin wild-type, Lane 2, NEO insertion in one FT1 allele (referred as a FT1^+/NEO^). C) Susceptibility to MTX of the FT1^+/NEO^ line. The ratio of drug EC_50_ values for the FT1^+/NEO^ parasites compared to wild-type parasites is indicated at the top of the histogram. The significance of the fold-increase in resistance to MTX for the single knockout line compared to wild-type parasites were was evaluated using unpaired two-tailed t-test. *, P<0.05.
(TIF)

S7 Fig Selected mutations in the folate transporter FT1 and their analysis by DNA editing. MTX EC_50_ was determined as the mean ± SD of three biological independent replicates for each mutant. The difference in EC_50_ of the mutants compared to the wild-type, referred as the resistance fold, were tested for significance using unpaired two-tailed t-test. *, P<0.05; **, P<0.01; ***, P<0.001.
(TIF)

S8 Fig Inactivation of one allele of FT5. A) Schematic representation of FT5 (top) and the puromycin inactivation cassette (PURO, bottom). The position of the primers (a and b) used for generating the 3’UTR probe for Southern Blot hybridization is depicted by arrowheads. B) Southern blot of wild-type and inactivated strains digested with DraI and BamHI after hybridization with a 3′UTR probe. Lane 1, *L*. *major* Friedlin wild-type; Lane 2, *L*. *major* Friedlin with one FT5 allele replaced by PURO, referred as the single knockout (SKO). C) Susceptibility of *L*. *major* Friedlin FT5^+/PURO^ line (i.e. SKO) to MTX. The ratio of MTX EC_50_ for SKO parasites compared to wild-type parasites is indicated at the top of histogram. Data are shown as means ± SD of three biological replicates. The significance of resistance ratios was tested using unpaired two-tailed t-test. ns, not significant.
(TIF)

S9 Fig Protein alignment of DHFR-TS from several *Leishmania* species. DHFR-TS of *L*. *major* Friedlin (LmjF.06.0860), *L*. *infantum* (LINF_060014300), *L*. *donovani* (LdBPK_060890.1), *L*. *mexicana* (LmxM.06.0860), *L*. *tropica* (LTRL590_060014000), *L*. *braziliensis* (LBRM2903_060015100) and *L*. *panamensis* (LPAL13_060014300) were aligned. Homologous regions are highlighted in grey and amino acids found as mutated in our Mut-Seq screen are indicated in bold.
(TIF)

S10 Fig Gene expression of wild-type and variant *DHFR-TS* and *PTR1* by real-time RT-PCR. A) *DHFR-TS* expression in *L*. *major* Friedlin overexpressing wild-type (DHFR) or mutated versions (DHFR^T107I^ or DHFR^E291K^) of DHFR-TS. The expression of *DHFR-TS* in a *L*. *major* Friedlin overexpressing *PTR1* (black) was used as the baseline chromosomal expression for the gene. B) *PTR1* expression in *L*. *major* Friedlin overexpressing wild-type (PTR1) or mutated versions (PTR1^A28T^ or PTR1^S253F^) of PTR1. The expression of *PTR1* in a *L*. *major* Friedlin overexpressing *DHFR-TS* (black) was used as the baseline chromosomal expression for the gene. The expression data was normalized using the housekeeping gene β-tubulin. *, p<0.05; **, p<0.01; ***, p<0.001 for the expression of *PTR1* or *DHFR-TS* compared to the chromosomal control (black). ns, not significant.
(TIF)

S11 Fig PTR1 DNA editing and absence of phenotype. PTR1 mutations detected in mutants B (triploid for chr23, see S1 Fig.) and T (tetraploid for chr23, see S1 Fig.) were integrated in *L*. *major* Friedlin wild-type (triploid for chr23, see S1 Fig.) by DNA editing but the edited cells were not more resistant to MTX than the control. B) Structure of PTR1 of *L*. *major*. PTR1 is a tetramer. Mutations found by NGS are highlighted in pink, the active site in light green, the co-factor NDP in light blue and the substrate folic acid in red. The difference in EC_50_ of the mutants compared to the wild-type, referred as the resistance fold, were tested for significance using unpaired two-tailed t-test. **, P<0.01; ***, P<0.001. ns, not significant.
(TIF)

S1 Table List of primers used. (DOCX)

S2 Table Sequence Reads Archive biosample accessions for samples sequenced in this study. (DOCX)

S3 Table List of genes mutated in at least two *L*. *major* MTX-resistant mutants generated by Mut-Seq. (XLSX)

S4 Table Sanger sequencing of CRISPR-edited cells resistant to MTX. (DOCX)
