# Supplementary figures and images for: Delving in folate metabolism in the parasite Leishmania major through a chemogenomic screen and methotrexate selection
Source: PLoS Negl Trop Dis. 2023 Jun 29;17(6):e0011458. doi: 10.1371/journal.pntd.0011458 (PMC10337921; doi:10.1371/journal.pntd.0011458)

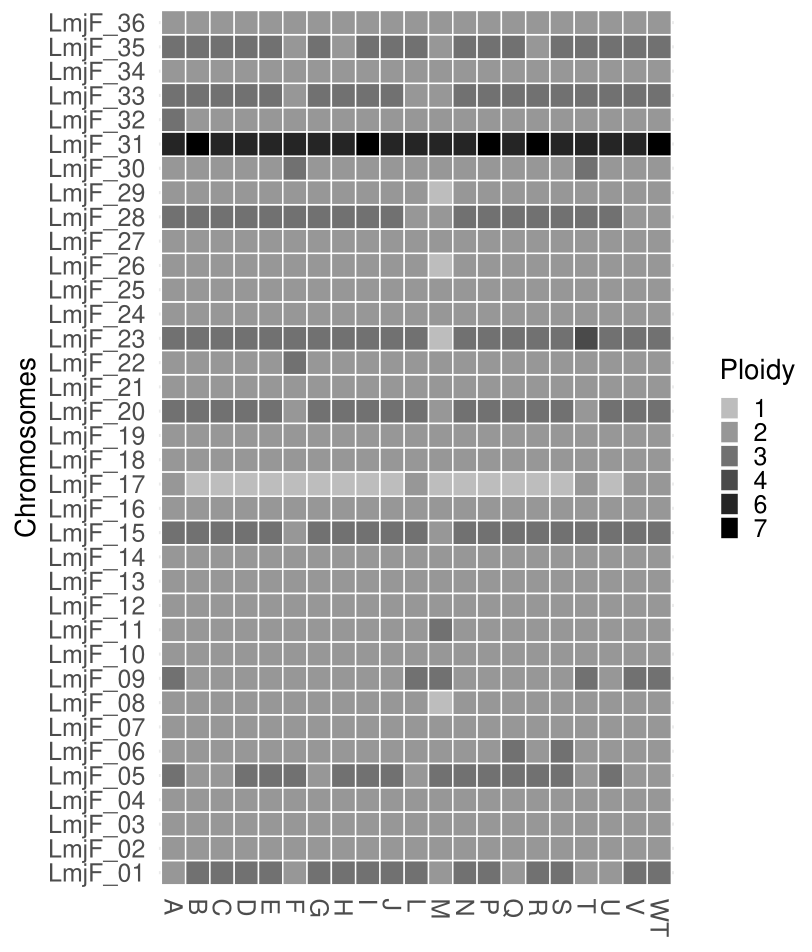

Supplement: S1 Fig — (TIF) [file pntd.0011458.s001.tif]

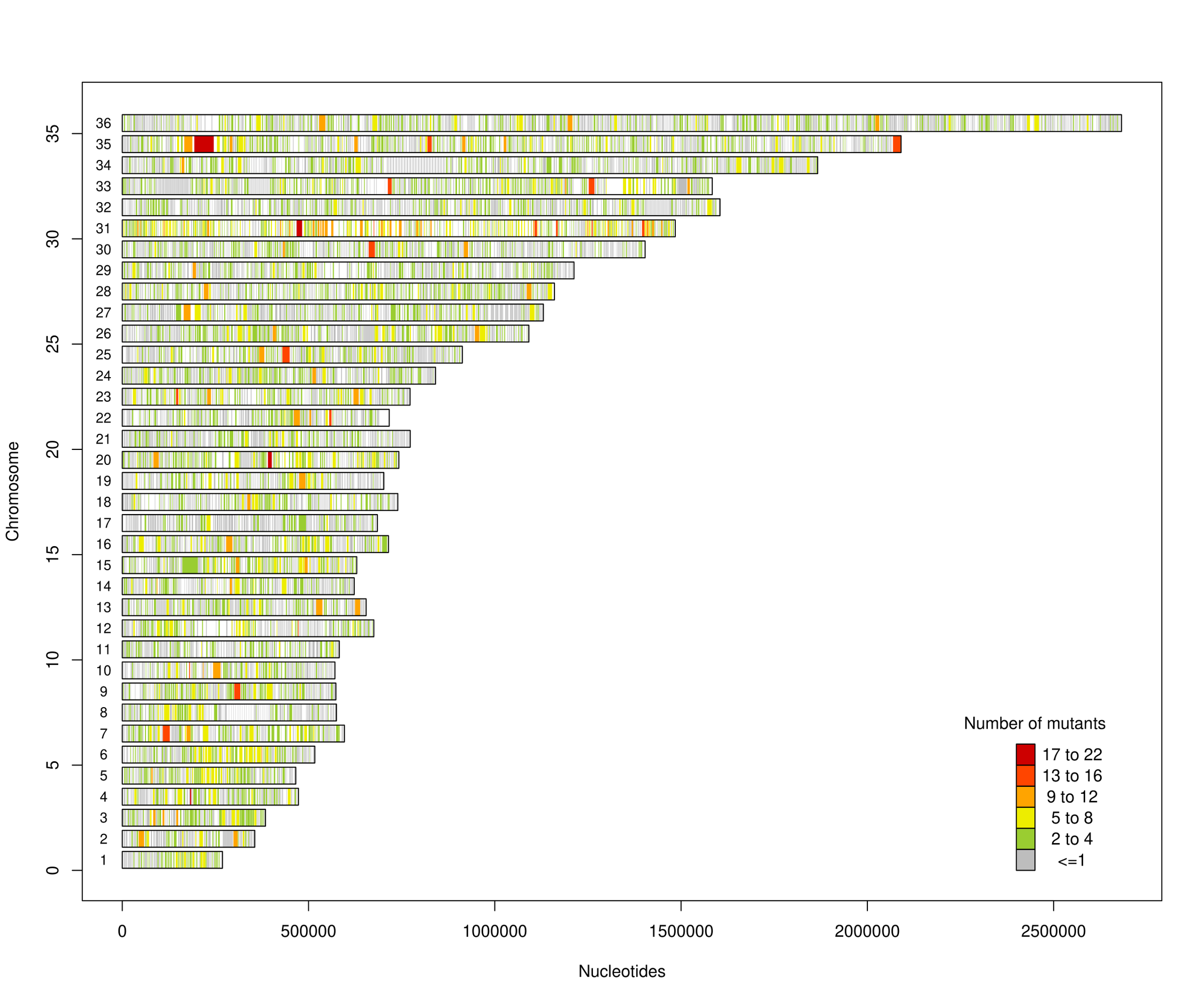

Supplement: S3 Fig — Genes for the 36 chromosomes are shown as bars colored according to their mutation frequency among the MTX-resistant mutants. Gray bars denote non-mutated genes. White segments correspond to intergenic regions. (TIF) [file pntd.0011458.s003.tif]

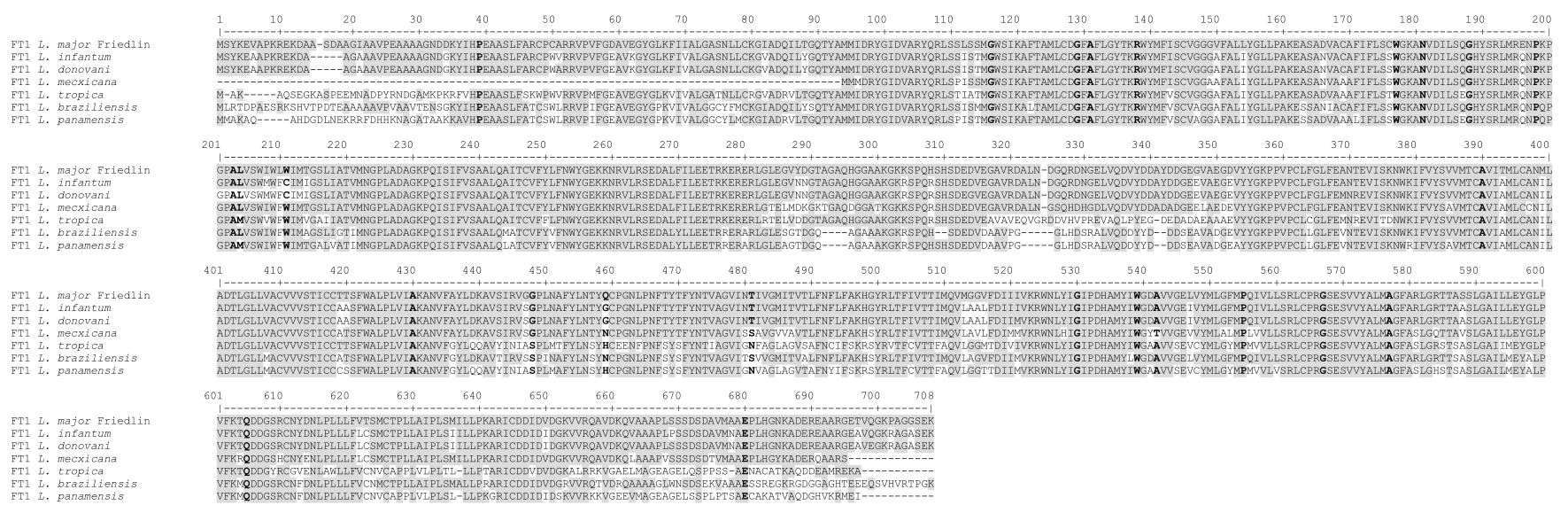

Supplement: S4 Fig — FT1 proteins of L. major Friedlin (LmjF.10.0385), L. infantum (LINF_100009300), L. donovani (LDHU3_10.0570), L. mexicana (LmxM.10.0370), L. tropica (LTRL590_000024200), L. braziliensis (LbrM.10.0400) and L. panamensis (LPMP_100340) were aligned. Homologous regions were highlighted in grey and amino acids found mutated in in the Mut-seq screen are shown in bold. (TIF) [file pntd.0011458.s004.tif]

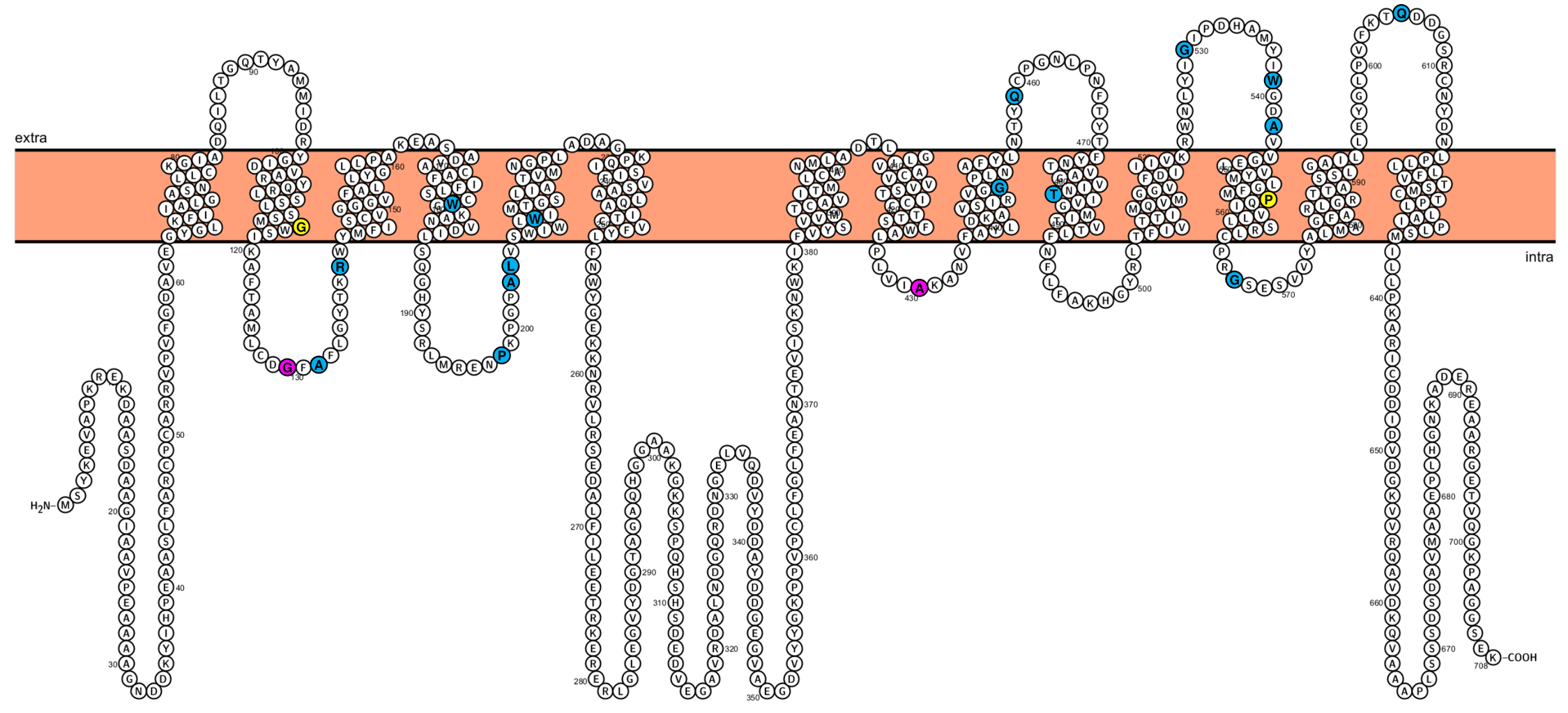

Supplement: S5 Fig — FT1 has fourteen transmembrane domains. Amino acids are represented by lettered circles. Amino acids mutated in FT1 are colored according to the following scheme: yellow for mutations found in mutant J, pink for mutations found in mutant N and blue for mutations found in mutants from our Mut-Seq screen that were not further studied. (TIF) [file pntd.0011458.s005.tif]

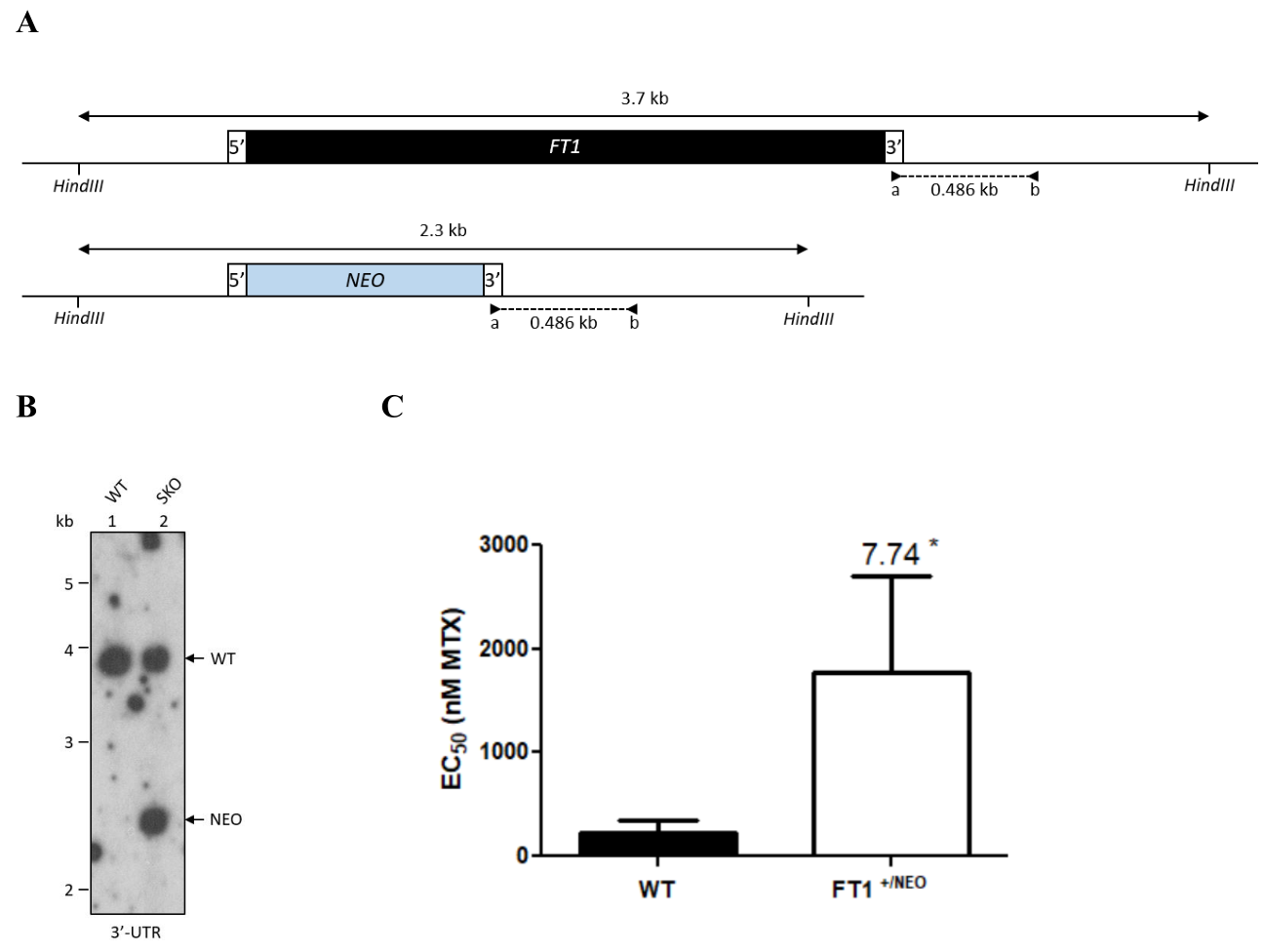

Supplement: S6 Fig — A) Schematic representation of the FT1 gene (top) and inactivation cassette with the neomycin selection marker (NEO, bottom). The position of the primers (a and b) used for the amplification of the probe for Southern Blot hybridization is depicted by arrowheads. B) Southern blot of genomic DNA derived from L. major Friedlin wild-type and FT1 single KO digested with HindIII and hybridized with a FT1 3′UTR probe. Lane 1, L. major Friedlin wild-type, Lane 2, NEO insertion in one FT1 allele (referred as a FT1+/NEO). C) Susceptibility to MTX of the FT1+/NEO line. The ratio of drug EC50 values for the FT1+/NEO parasites compared to wild-type parasites is indicated at the top of the histogram. The significance of the fold-increase in resistance to MTX for the single knockout line compared to wild-type parasites were was evaluated using unpaired two-tailed t-test. *, P<0.05. (TIF) [file pntd.0011458.s006.tif]

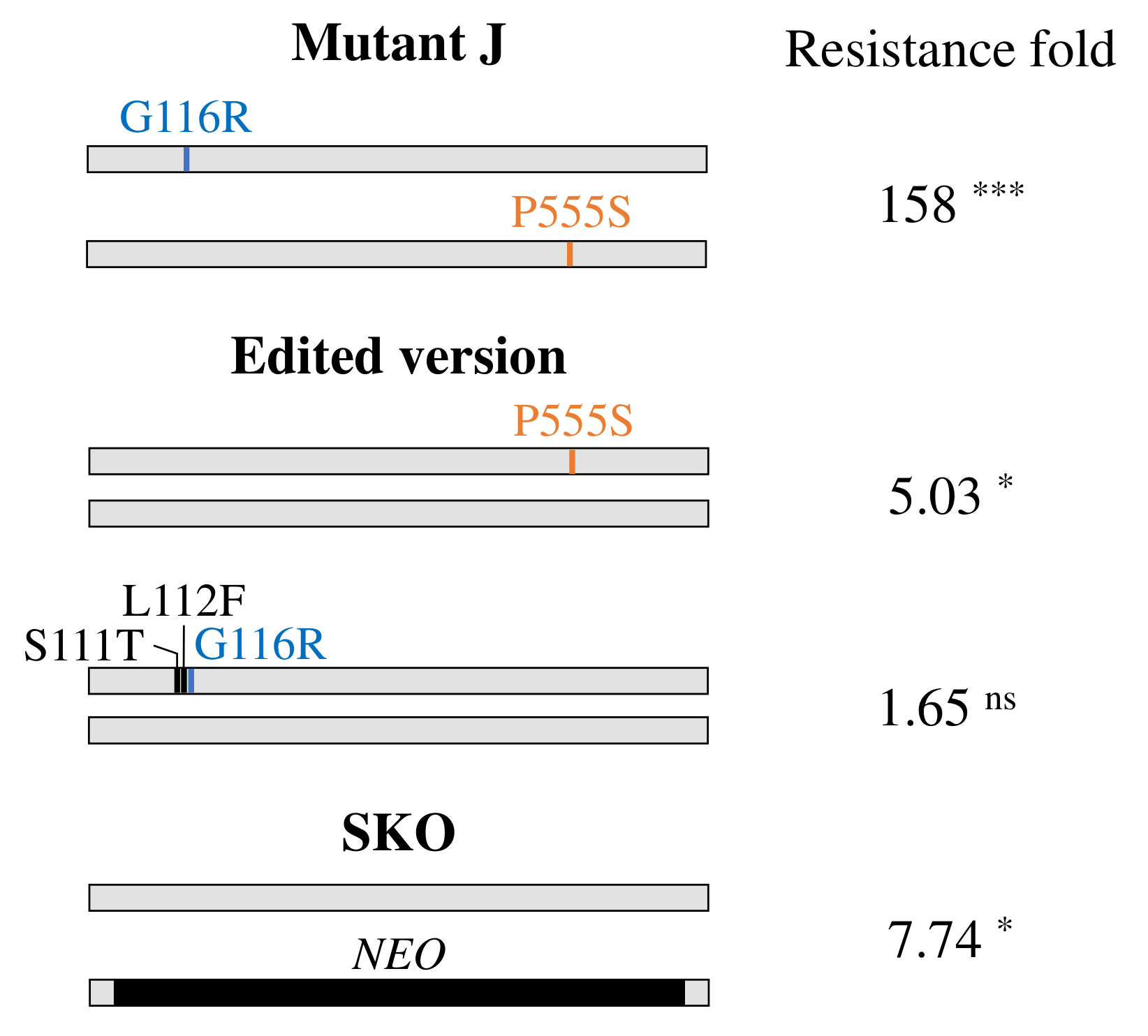

Supplement: S7 Fig — MTX EC50 was determined as the mean ± SD of three biological independent replicates for each mutant. The difference in EC50 of the mutants compared to the wild-type, referred as the resistance fold, were tested for significance using unpaired two-tailed t-test. *, P<0.05; **, P<0.01; ***, P<0.001. (TIF) [file pntd.0011458.s007.tif]

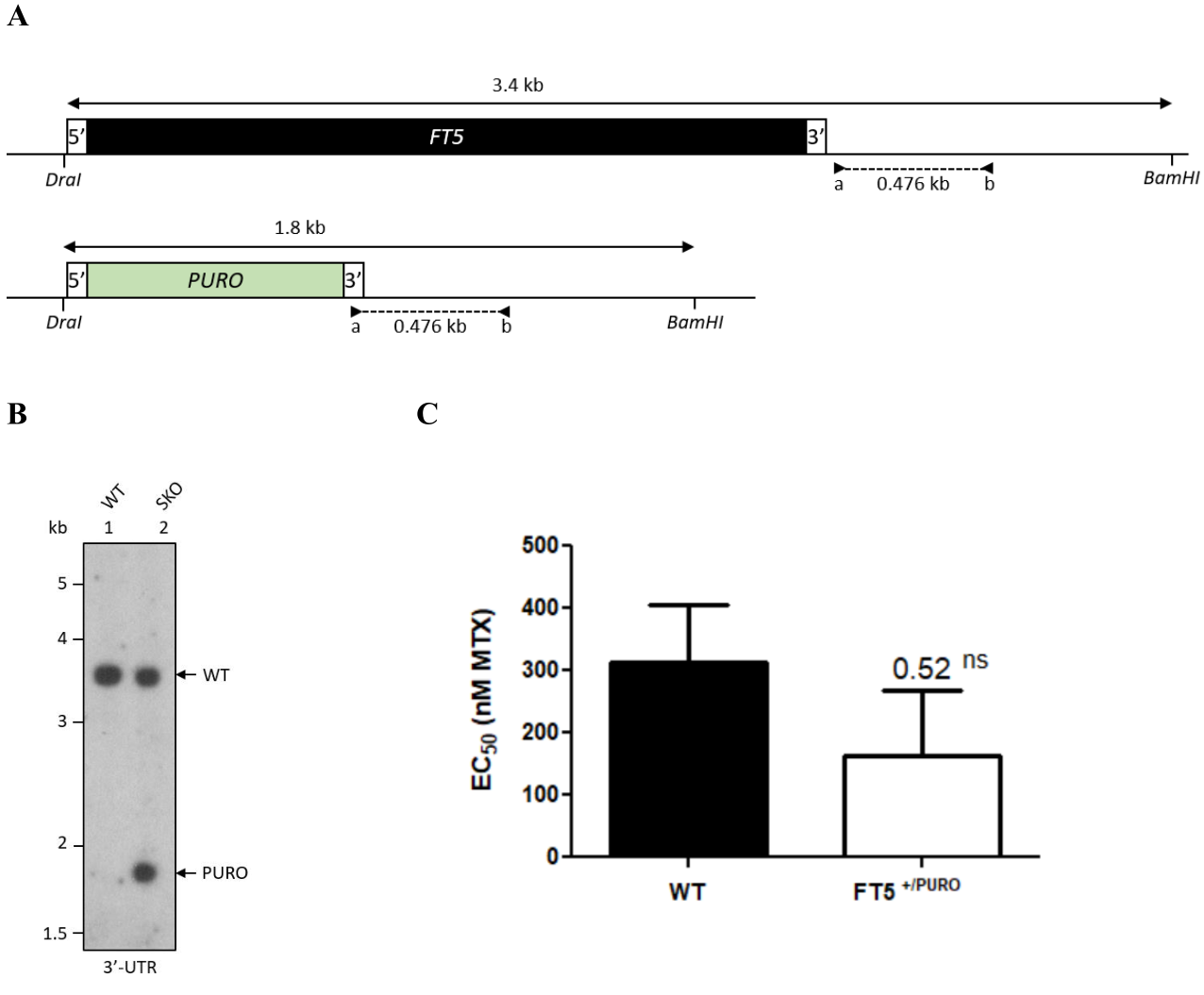

Supplement: S8 Fig — A) Schematic representation of FT5 (top) and the puromycin inactivation cassette (PURO, bottom). The position of the primers (a and b) used for generating the 3’UTR probe for Southern Blot hybridization is depicted by arrowheads. B) Southern blot of wild-type and inactivated strains digested with DraI and BamHI after hybridization with a 3′UTR probe. Lane 1, L. major Friedlin wild-type; Lane 2, L. major Friedlin with one FT5 allele replaced by PURO, referred as the single knockout (SKO). C) Susceptibility of L. major Friedlin FT5+/PURO line (i.e. SKO) to MTX. The ratio of MTX EC50 for SKO parasites compared to wild-type parasites is indicated at the top of histogram. Data are shown as means ± SD of three biological replicates. The significance of resistance ratios was tested using unpaired two-tailed t-test. ns, not significant. (TIF) [file pntd.0011458.s008.tif]

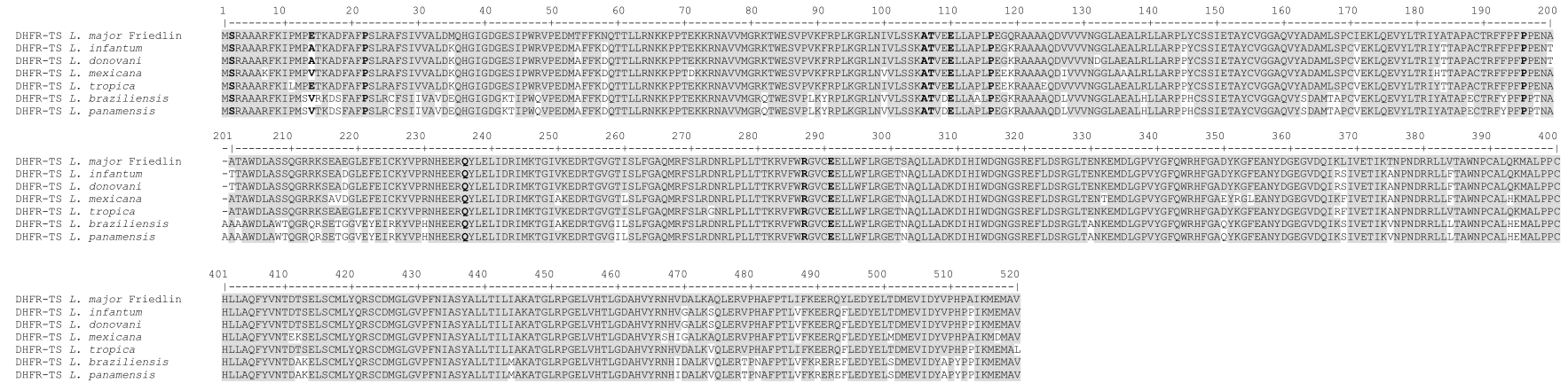

Supplement: S9 Fig — DHFR-TS of L. major Friedlin (LmjF.06.0860), L. infantum (LINF_060014300), L. donovani (LdBPK_060890.1), L. mexicana (LmxM.06.0860), L. tropica (LTRL590_060014000), L. braziliensis (LBRM2903_060015100) and L. panamensis (LPAL13_060014300) were aligned. Homologous regions are highlighted in grey and amino acids found as mutated in our Mut-Seq screen are indicated in bold. (TIF) [file pntd.0011458.s009.tif]

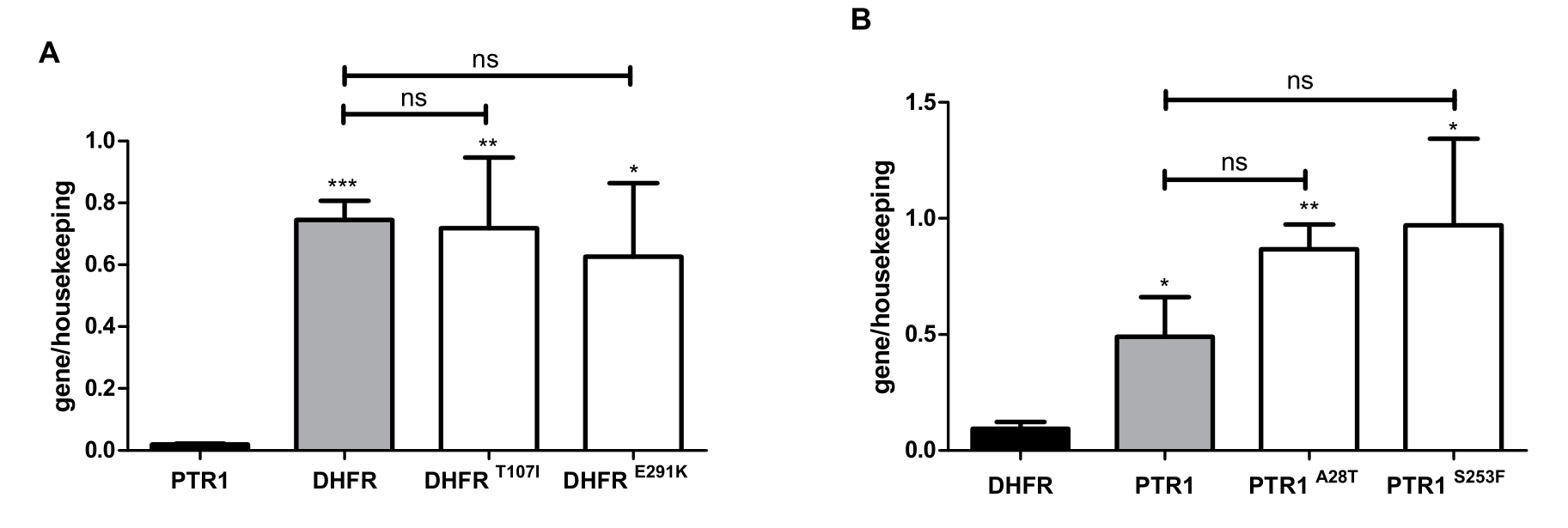

Supplement: S10 Fig — A) DHFR-TS expression in L. major Friedlin overexpressing wild-type (DHFR) or mutated versions (DHFRT107I or DHFRE291K) of DHFR-TS. The expression of DHFR-TS in a L. major Friedlin overexpressing PTR1 (black) was used as the baseline chromosomal expression for the gene. B) PTR1 expression in L. major Friedlin overexpressing wild-type (PTR1) or mutated versions (PTR1A28T or PTR1S253F) of PTR1. The expression of PTR1 in a L. major Friedlin overexpressing DHFR-TS (black) was used as the baseline chromosomal expression for the gene. The expression data was normalized using the housekeeping gene β-tubulin. *, p<0.05; **, p<0.01; ***, p<0.001 for the expression of PTR1 or DHFR-TS compared to the chromosomal control (black). ns, not significant. (TIF) [file pntd.0011458.s010.tif]

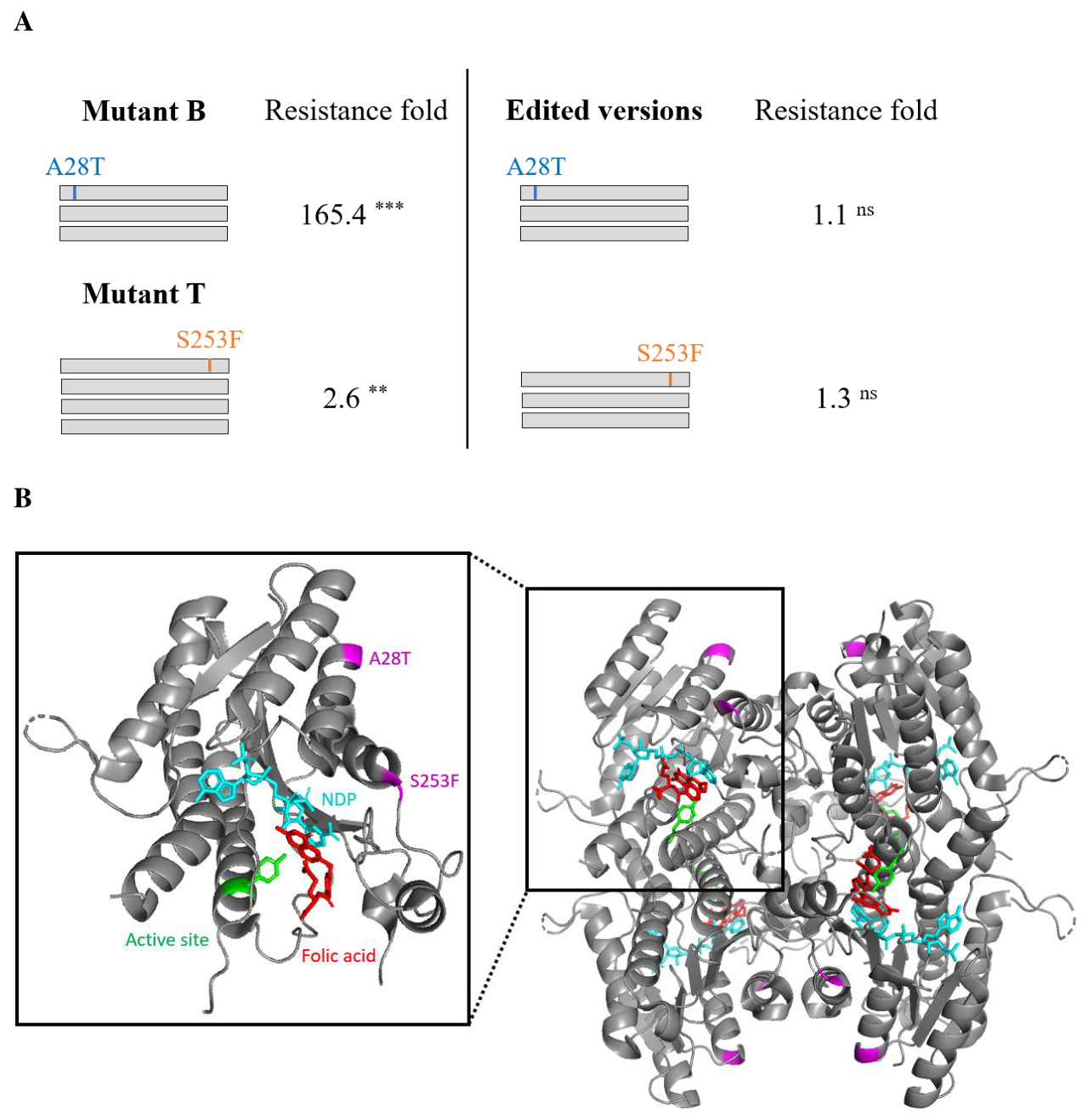

Supplement: S11 Fig — PTR1 mutations detected in mutants B (triploid for chr23, see S1 Fig.) and T (tetraploid for chr23, see S1 Fig.) were integrated in L. major Friedlin wild-type (triploid for chr23, see S1 Fig.) by DNA editing but the edited cells were not more resistant to MTX than the control. B) Structure of PTR1 of L. major. PTR1 is a tetramer. Mutations found by NGS are highlighted in pink, the active site in light green, the co-factor NDP in light blue and the substrate folic acid in red. The difference in EC50 of the mutants compared to the wild-type, referred as the resistance fold, were tested for significance using unpaired two-tailed t-test. **, P<0.01; ***, P<0.001. ns, not significant. (TIF) [file pntd.0011458.s011.tif]
