# Supplementary material for: Delving in folate metabolism in the parasite Leishmania major through a chemogenomic screen and methotrexate selection
Source: PLoS Negl Trop Dis. 2023 Jun 29;17(6):e0011458. doi: 10.1371/journal.pntd.0011458 (PMC10337921; doi:10.1371/journal.pntd.0011458)
Supplement: S2 Fig — LmjF.10.0380 sequence is highlighted in blue, FT1 in green and LmjF.10.0390 in pink. SNPs are highlighted in grey. (PDF) [file pntd.0011458.s002.pdf]

```
                             1       10        20        30        40        50        60        70        80        90       100       110       120       130       140       150       160       170       180       190       200
                             |--------+---------+---------+---------+---------+---------+---------+---------+---------+---------+---------+---------+---------+---------+---------+---------+---------+---------+---------+---------|
WT                           ATGTCCCACAAGGAGGCCGCTCCCAAGCGCGAGAAGGATGCCGCCTCAGACGCCGCGGGAAATGATGACAAGTACATCCACCCCGAGGCAGCATCCCTGTTCGCCAGGTGCCCGTGGGCTCGCCGCATCCCCGTGTTTGGTGAAGCTGTCGAGGGCTACGGGCCCAAGGTGATCGTCGCCCTCGGTGGGTGCTACCTGCT
Mutant B recombinant type 1  ATGTCCCACAAGGAGGCCGCTCCCAAGCGCGAGAAGGATGCCGCCTCAGACGCCGCGGGAAATGATGACAAGTACATCCACCCCGAGGCAGCATCCCTGTTCGCCAGGTGCCCGTGGGCTCGCCGCATCCCCGTGTTTGGTGAAGCTGTCGAGGGCTACGGGCCCAAGGTGATCGTCGCCCTCGGTGGGTGCTACCTGCT
Mutant B recombinant type 2  ATGTCCCACAAGGAGGCCGCTCCCAAGCGCGAGAAGGATGCCGCCTCAGACGCCGCGGGAAATGATGACAAGTACATCCACCCCGAGGCAGCATCCCTGTTCGCCAGGTGCCCGTGGGCTCGCCGCATCCCCGTGTTTGGTGAAGCTGTCGAGGGCTACGGGCCCAAGGTGATCGTCGCCCTCGGTGGGTGCTACCTGCT
Mutant G mutation            ATGTCCCACAAGGAGGCCGCTCCCAAGCGCGAGAAGGATGCCGCCTCAGACGCCGCGGGAAATGATGACAAGTACATCCACCCCGAGGCAGCATCCCTGTTCGCCAGGTGCCCGTGGGCTCGCCGCATCCCCGTGTTTGGTGAAGCTGTCGAGGGCTACGGGCCCAAGGTGATCGTCGCCCTCGGTGGGTGCTACCTGCT
Mutant G recombinant         ATGTCCCACAAGGAGGCCGCTCCCAAGCGCGAGAAGGATGCCGCCTCAGACGCCGCGGGAAATGATGACAAGTACATCCACCCCGAGGCAGCATCCCTGTTCGCCAGGTGCCCGTGGGCTCGCCGCATCCCCGTGTTTGGTGAAGCTGTCGAGGGCTACGGGCCCAAGGTGATCGTCGCCCTCGGTGGGTGCTACCTGCT
Mutant E mutation            ATGTCCCACAAGGAGGCCGCTCCCAAGCGCGAGAAGGATGCCGCCTCAGACGCCGCGGGAAATGATGACAAGTACATCCACCCCGAGGCAGCATCCCTGTTCGCCAGGTGCCCGTGGGCTCGCCGCATCCCCGTGTTTGGTGAAGCTGTCGAGGGCTACGGGCCCAAGGTGATCGTCGCCCTCGGTGGGTGCTACCTGCT
Mutant E gene conversion     ATGTCCCACAAGGAGGCCGCTCCCAAGCGCGAGAAGGATGCCGCCTCAGACGCCGCGGGAAATGATGACAAGTACATCCACCCCGAGGCAGCATCCCTGTTCGCCAGGTGCCCGTGGGCTCGCCGCATCCCCGTGTTTGGTGAAGCTGTCGAGGGCTACGGGCCCAAGGTGATCGTCGCCCTCGGTGGGTGCTACCTGCT

                           201       210       220       230       240       250       260       270       280       290       300       310       320       330       340       350       360       370       380       390       400
                             |--------+---------+---------+---------+---------+---------+---------+---------+---------+---------+---------+---------+---------+---------+---------+---------+---------+---------+---------+---------|
WT                           CTGCAAGGGCGTCGCAGATCAGATTCTGCGCGGTCAGACGTACGCCATGATGATGGATCGCTACGGCATCGACGTGGCCCGCTACCAGCGCCTGTCTCCGATTTCATCCATGGGGTGGTCCATCAAGGCCTTCACCGCGATGCTCTGCGACGGCTTCGCCTTCCTCGGCTACACGAAGCGCTGGTACATGTTCATCTCCT
Mutant B recombinant type 1  CTGCAAGGGCGTCGCAGATCAGATTCTGCGCGGTCAGACGTACGCCATGATGATGGATCGCTACGGCATCGACGTGGCCCGCTACCAGCGCCTGTCTCCGATTT------------------------------------------------------------------------------------------------
Mutant B recombinant type 2  CTGCAAGGGCGTCGCAGATCAGATTCTGCGCGGTCAGACGTACGCCATGATGATGGATCGCTACGGCATCGACGTGGCCCGCTACCAGCGCCTGTCTCCGATTTCATC--------------------------------------------------------------------------------------------
Mutant G mutation            CTGCAAGGGCGTCGCAGATCAGATTCTGCGCGGTCAGACGTACGCCATGATGATGGATCGCTACGGCATCGACGTGGCCCGCTACCAGCGCCTGTCTCCGATTTCATCCATGGGGTGGTCCATCAAGGCCTTCACCGCGATGCTCTGCGACGGCTTCGCCTTCCTCGGCTACACGAAGCGCTGGTACATGTTCATCTCCT
Mutant G recombinant         CTGCAAGGGCGTCGCAGATCAGATTCTGCGCGGTCAGACGTACGCCATGATGATGGATCGCTACGGCATCGACGTGGCCCGCTACCAGCGCCTGTCT-------------------------------------------------------------------------------------------------------
Mutant E mutation            CTGCAAGGGCGTCGCAGATCAGATTCTGCGCGGTCAGACGTACGCCATGATGATGGATCGCTACGGCATCGACGTGGCCCGCTACCAGCGCCTGTCTCCGATTTCATCCATGGGGTGGTCCATCAAGGCCTTCACCGCGATGCTCTGCGACGGCTTCGCCTTCCTCGGCTACACGAAGCGCTGGTACATGTTCATCTCCT
Mutant E gene conversion     CTGCAAGGGCGTCGCAGATCAGATTCTGCGCGGTCAGACGTACGCCATGATGATGGATCGCTACGGCATCGACGTGGCCCGCTACCAGCGCCTGTCTCCGATTTCATCCATGGGGTGGTCCATCAAGGCCTTCACCGCGATGCTCTGCGACGGCTTCGCCTTCCTCGGCTACACGAAGCGCTGGTACATGTTCATCTCCT

                           401       410       420       430       440       450       460       470       480       490       500       510       520       530       540       550       560       570       580       590       600
                             |--------+---------+---------+---------+---------+---------+---------+---------+---------+---------+---------+---------+---------+---------+---------+---------+---------+---------+---------+---------|
WT                           GTGTCGGCGGTGGTGCGTTCGCGCTGATCTACGGCCTGCTGCCCGCGAAGGAGGCGTCGGCTGATGTGGCGTGTGCCTTCATCTTCCTGTCGTGCTGGGGCAAGGCCAACGTGGATATCCTGTCCCAGGGCCATTACAGTCGACTGATGCGCCAGAACCCGAAGCCTGGCCCGTCCATGGTGAGCTGGATCTGGTTCTGG
Mutant B recombinant type 1  --------------------------------------------------------------------------------------------------------------------------------------------------------------------------------------------------------
Mutant B recombinant type 2  --------------------------------------------------------------------------------------------------------------------------------------------------------------------------------------------------------
Mutant G mutation            GTGTCGGCGGTGGTGCGTTCGCGCTGATCTACGGCCTGCTGCCCGCGAAGGAGGCGTCGGCTGATGTGGCGTGTGCCTTCATCTTCCTGTCGTGCTGGGGCAAGGCCAACGTGGATATCCTGTCCCAGGGCCATTACAGTCGACTGATGCGCCAGAACCCGAAGCCTGGCCCGTCCATGGTGAGCTGGATCTGGTTCTGG
Mutant G recombinant         --------------------------------------------------------------------------------------------------------------------------------------------------------------------------------------------------------
Mutant E mutation            GTGTCGGCGGTGGTGCGTTCGCGCTGATCTACGGCCTGCTGCCCGCGAAGGAGGCGTCGGCTGATGTGGCGTGTGCCTTCATCTTCCTGTCGTGCTGGGGCAAGGCCAACGTGGATATCCTGTCCCAGGGCCATTACAGTCGACTGATGCGCCAGAACCCGAAGCCTGGCCCGTCCATGGTGAGCTGGATCTGGTTCTGG
Mutant E gene conversion     GTGTCGGCGGTGGTGCGTTCGCGCTGATCTACGGCCTGCTGCCCGCGAAGGAGGCGTCGGCTGATGTGGCGTGTGCCTTCATCTTCCTGTCGTGCTGGGGCAAGGCCAACGTGGATATCCTGTCCCAGGGCCATTACAGTCGACTGATGCGCCAGAACCCGAAGCCTGGCCCGTCCATGGTGAGCTGGATCTGGTTCTGG

                           601       610       620       630       640       650       660       670       680       690       700       710       720       730       740       750       760       770       780       790       800
                             |--------+---------+---------+---------+---------+---------+---------+---------+---------+---------+---------+---------+---------+---------+---------+---------+---------+---------+---------+---------|
WT                           ATCATGACCGGCTCGCTCATCGCGACTGTGATGAACGGCCCGCTCGCGGATGCCGGGAAGCCGCAGATCAGCATCTTCGTGTCTGCCGCGCTGCAGCTCATCACCTGCGTCTTCTACCTGTTCAACTGGTACGGGGAGAAGAAGAACCGCGTGCTGCGCTCCGAGGACGCGCTGTTTATTCTGGAGGAGACCCGCAAGGA
Mutant B recombinant type 1  --------------------------------------------------------------------------------------------------------------------------------------------------------------------------------------------------------
Mutant B recombinant type 2  --------------------------------------------------------------------------------------------------------------------------------------------------------------------------------------------------------
Mutant G mutation            ATCATGACCGGCTCGCTCATCGCGACTGTGATGAACGGCCCGCTCGCGGATGCCGGGAAGCCGCAGATCAGCATCTTCGTGTCTGCCGCGCTGCAGCTCATCACCTGCGTCTTCTACCTGTTCAACTGGTACGGGGAGAAGAAGAACCGCGTGCTGCGCTCCGAGGACGCGCTGTTTATTCTGGAGGAGACCCGCAAGGA
Mutant G recombinant         --------------------------------------------------------------------------------------------------------------------------------------------------------------------------------------------------------
Mutant E mutation            ATCATGACCGGCTCGCTCATCGCGACTGTGATGAACGGCCCGCTCGCGGATGCCGGGAAGCCGCAGATCAGCATCTTCGTGTCTGCCGCGCTGCAGCTCATCACCTGCGTCTTCTACCTGTTCAACTGGTACGGGGAGAAGAAGAACCGCGTGCTGCGCTCCGAGGACGCGCTGTTTATTCTGGAGGAGACCCGCAAGGA
Mutant E gene conversion     ATCATGACCGGCTCGCTCATCGCGACTGTGATGAACGGCCCGCTCGCGGATGCCGGGAAGCCGCAGATCAGCATCTTCGTGTCTGCCGCGCTGCAGCTCATCACCTGCGTCTTCTACCTGTTCAACTGGTACGGGGAGAAGAAGAACCGCGTGCTGCGCTCCGAGGACGCGCTGTTTATTCTGGAGGAGACCCGCAAGGA

                           801       810       820       830       840       850       860       870       880       890       900       910       920       930       940       950       960       970       980       990      1000
                             |--------+---------+---------+---------+---------+---------+---------+---------+---------+---------+---------+---------+---------+---------+---------+---------+---------+---------+---------+---------|
WT                           GCGTGAGCGCCTGGGCACTGAACTGATGGACGACGGCACGGCGGGTGCGCAGCATGGTGGTGCGGCGAAGGGGAAGAAGAGCCCGCAGCACTCGCACTCGGATGAGGACGTGGAGGGTGCCGTACGGGACGCCCTCAACGATGGTCAGCGCGACAACGGTGAACTTGTGCAGGACGTCTACGACGACGCGTATGACGACG
Mutant B recombinant type 1  --------------------------------------------------------------------------------------------------------------------------------------------------------------------------------------------------------
Mutant B recombinant type 2  --------------------------------------------------------------------------------------------------------------------------------------------------------------------------------------------------------
Mutant G mutation            GCGTGAGCGCCTGGGCACTGAACTGATGGACGACGGCACGGCGGGTGCGCAGCATGGTGGTGCGGCGAAGGGGAAGAAGAGCCCGCAGCACTCGCACTCGGATGAGGACGTGGAGGGTGCCGTACGGGACGCCCTCAACGATGGTCAGCGCGACAACGGTGAACTTGTGCAGGACGTCTACGACGACGCGTATGACGACG
Mutant G recombinant         --------------------------------------------------------------------------------------------------------------------------------------------------------------------------------------------------------
Mutant E mutation            GCGTGAGCGCCTGGGCACTGAACTGATGGACGACGGCACGGCGGGTGCGCAGCATGGTGGTGCGGCGAAGGGGAAGAAGAGCCCGCAGCACTCGCACTCGGATGAGGACGTGGAGGGTGCCGTACGGGACGCCCTCAACGATGGTCAGCGCGACAACGGTGAACTTGTGCAGGACGTCTACGACGACGCGTATGACGACG
Mutant E gene conversion     GCGTGAGCGCCTGGGCACTGAACTGATGGACGACGGCACGGCGGGTGCGCAGCATGGTGGTGCGGCGAAGGGGAAGAAGAGCCCGCAGCACTCGCACTCGGATGAGGACGTGGAGGGTGCCGTACGGGACGCCCTCAACGATGGTCAGCGCGACAACGGTGAACTTGTGCAGGACGTCTACGACGACGCGTATGACGACG

                          1001      1010      1020      1030      1040      1050      1060      1070      1080      1090      1100      1110      1120      1130      1140      1150      1160      1170      1180      1190      1200
                             |--------+---------+---------+---------+---------+---------+---------+---------+---------+---------+---------+---------+---------+---------+---------+---------+---------+---------+---------+---------|
WT                           GCGAGGGGGTGGCCGAGGGCGATGTGTACTACGGCAAGCCGCCGGTGCCGTGCCTGTTCGGGCTGTTCGAGGCAAACACGGAGGTGATTTCGAAGAACTGGAAGATCTTCGTGTACAGCGTCGTCATGACCTGTGCTGTGATCACGATGCTGTGTGCCAACATGCTGGCCGACACGCTGGGCCTCCTGATTGCGTGCGTC
Mutant B recombinant type 1  --------------------------------------------------------------------------------------------------------------------------------------------------------------------------------------------------------
Mutant B recombinant type 2  --------------------------------------------------------------------------------------------------------------------------------------------------------------------------------------------------------
Mutant G mutation            GCGAGGGGGTGGCCGAGGGCGATGTGTACTACGGCAAGCCGCCGGTGCCGTGCCTGTTCGGGCTGTTCGAGGCAAACACGGAGGTGATTTCGAAGAACTGGAAGATCTTCGTGTACAGCGTCGTCATGACCTGTGCTGTGATCACGATGCTGTGTGCCAACATGCTGGCCGACACGCTGGGCCTCCTGATTGCGTGCGTC
Mutant G recombinant         --------------------------------------------------------------------------------------------------------------------------------------------------------------------------------------------------------
Mutant E mutation            GCGAGGGGGTGGCCGAGGGCGATGTGTACTACGGCAAGCCGCCGGTGCCGTGCCTGTTCGGGCTGTTCGAGGCAAACACGGAGGTGATTTCGAAGAACTGGAAGATCTTCGTGTACAGCGTCGTCATGACCTGTGCTGTGATCACGATGCTGTGTGCCAACATGCTGGCCGACACGCTGGGCCTCCTGATTGCGTGCGTC
Mutant E gene conversion     GCGAGGGGGTGGCCGAGGGCGATGTGTACTACGGCAAGCCGCCGGTGCCGTGCCTGTTCGGGCTGTTCGAGGCAAACACGGAGGTGATTTCGAAGAACTGGAAGATCTTCGTGTACAGCGTCGTCATGACCTGTGCTGTGATCACGATGCTGTGTGCCAACATGCTGGCCGACACGCTGGGCCTCCTGATTGCGTGCGTC

                          1201      1210      1220      1230      1240      1250      1260      1270      1280      1290      1300      1310      1320      1330      1340      1350      1360      1370      1380      1390      1400
                             |--------+---------+---------+---------+---------+---------+---------+---------+---------+---------+---------+---------+---------+---------+---------+---------+---------+---------+---------+---------|
WT                           GTTGTGTCGACCATCTGCTGCGCCACGTCCTTCTGGGCCCTGCCGCTGGTGATTGCGAAAGCCAACGTCTTCGGATACCTGCAGATGGCTGTTTCCATCCGTGTCAGCAGTCCCGTGAACGCGTTCTTTCTGAACAACTATCAGTGCCCTGGCAACCTGCCGAACTTCACCTACACCTTCTACGGCACGGTGGCGGGCGT
Mutant B recombinant type 1  --------------------------------------------------------------------------------------------------------------------------------------------------------------------------------------------------------
Mutant B recombinant type 2  --------------------------------------------------------------------------------------------------------------------------------------------------------------------------------------------------------
Mutant G mutation            GTTGTGTCGACCATCTGCTGCGCCACGTCCTTCTGGGCCCTGCCGCTGGTGATTGCGAAAGCCAACGTCTTCGGATACCTGCAGATGGCTGTTTCCATCCGTGTCAGCAGTCCCGTGAACGCGTTCTTTCTGAACAACTATCAGTGCCCTGGCAACCTGCCGAACTTCACCTACACCTTCTACGGCACGGTGGCGGGCGT
Mutant G recombinant         --------------------------------------------------------------------------------------------------------------------------------------------------------------------------------------------------------
Mutant E mutation            GTTGTGTCGACCATCTGCTGCGCCACGTCCTTCTGGGCCCTGCCGCTGGTGATTGCGAAAGCCAACGTCTTCGGATACCTGCAGATGGCTGTTTCCATCCGTGTCAGCAGTCCCGTGAACGCGTTCTTTCTGAACAACTATCAGTGCCCTGGCAACCTGCCGAACTTCACCTACACCTTCTACGGCACGGTGGCGGGCGT
Mutant E gene conversion     GTTGTGTCGACCATCTGCTGCGCCACGTCCTTCTGGGCCCTGCCGCTGGTGATTGCGAAAGCCAACGTCTTCGGATACCTGCAGATGGCTGTTTCCATCCGTGTCAGCAGTCCCGTGAACGCGTTCTTTCTGAACAACTATCAGTGCCCTGGCAACCTGCCGAACTTCACCTACACCTTCTACGGCACGGTGGCGGGCGT

                          1401      1410      1420      1430      1440      1450      1460      1470      1480      1490      1500      1510      1520      1530      1540      1550      1560      1570      1580      1590      1600
                             |--------+---------+---------+---------+---------+---------+---------+---------+---------+---------+---------+---------+---------+---------+---------+---------+---------+---------+---------+---------|
WT                           GATTAGCAGTGTTGTTGGTATGATTACCGTGACGCTGTTCAACTTCCTGTTCGCGAAGCATGGCTACCGCCTCACCTTCATTGTGACGACAATCATGCAGGTTATGGGTGGTGTGTTCGACATCATCATCGTGAAGCGGTGGAACCTGCACATTGGCATCCCTGACCACGCCATGTACATCTGGGGTGATGCTGTTGTGG
Mutant B recombinant type 1  --------------------------------------------------------------------------------------------------------------------------------------------------------------------------------------------------------
Mutant B recombinant type 2  --------------------------------------------------------------------------------------------------------------------------------------------------------------------------------------------------------
Mutant G mutation            GATTAGCAGTGTTGTTGGTATGATTACCGTGACGCTGTTCAACTTCCTGTTCGCGAAGCATGGCTACCGCCTCACCTTCATTGTGACGACAATCATGCAGGTTATGGGTGGTGTGTTCGACATCATCATCGTGAAGCGGTGGAACCTGCACATTGGCATCCCTGACCACGCCATGTACATCTGGGGTGATGCTGTTGTGG
Mutant G recombinant         --------------------------------------------------------------------------------------------------------------------------------------------------------------------------------------------------------
Mutant E mutation            GATTAGCAGTGTTGTTGGTATGATTACCGTGACGCTGTTCAACTTCCTGTTCGCGAAGCATGGCTACCGCCTCACCTTCATTGTGACGACAATCATGCAGGTTATGGGTGGTGTGTTCGACATCATCATCGTGAAGCGGTGGAACCTGCACATTGGCATCCCTGACCACGCCATGTACATCTGGGGTGATGCTGTTGTGG
Mutant E gene conversion     GATTAGCAGTGTTGTTGGTATGATTACCGTGACGCTGTTCAACTTCCTGTTCGCGAAGCATGGCTACCGCCTCACCTTCATTGTGACGACAATCATGCAGGTTATGGGTGGTGTGTTCGACATCATCATCGTGAAGCGGTGGAACCTGCACATTGGCATCCCTGACCACGCCATGTACATCTGGGGTGATGCTGTTGTGG

                          1601      1610      1620      1630      1640      1650      1660      1670      1680      1690      1700      1710      1720      1730      1740      1750      1760      1770      1780      1790      1800
                             |--------+---------+---------+---------+---------+---------+---------+---------+---------+---------+---------+---------+---------+---------+---------+---------+---------+---------+---------+---------|
WT                           GTGAGCTCGTGTACATGCTTGGCTGGATGCCGATGATCGTGCTGCTGTCTCGCCTGTGCCCTCGTGGCTCGGAGAGTGTCGTGTATGCGCTGATGGCGGGCTTCTCCAACCTTGGCCAGACCACCGCGGCGTCCCTCGGTGCGATCATCATGGAGTACGGCCTGCCTGTGTTCAAGACCCAGGATGACGGGTCTCGCTGC
Mutant B recombinant type 1  --------------------------------------------------------------------------------------------------------------------------------------------------------------------------------------------------------
Mutant B recombinant type 2  --------------------------------------------------------------------------------------------------------------------------------------------------------------------------------------------------------
Mutant G mutation            GTGAGCTCGTGTACATGCTTGGCTGGATGCCGATGATCGTGCTGCTGTCTCGCCTGTGCCCTCGTGGCTCGGAGAGTGTCGTGTATGCGCTGATGGCGGGCTTCTCCAACCTTGGCCAGACCACCGCGGCGTCCCTCGGTGCGATCATCATGGAGTACGGCCTGCCTGTGTTCAAGACCCAGGATGACGGGTCTCGCTGC
Mutant G recombinant         --------------------------------------------------------------------------------------------------------------------------------------------------------------------------------------------------------
Mutant E mutation            GTGAGCTCGTGTACATGCTTGGCTGGATGCCGATGATCGTGCTGCTGTCTCGCCTGTGCCCTCGTGGCTCGGAGAGTGTCGTGTATGCGCTGATGGCGGGCTTCTCCAACCTTGGCCAGACCACCGCGGCGTCCCTCGGTGCGATCATCATGGAGTACGGCCTGCCTGTGTTCAAGACCCAGGATGACGGGTCTCGCTGC
Mutant E gene conversion     GTGAGCTCGTGTACATGCTTGGCTGGATGCCGATGATCGTGCTGCTGTCTCGCCTGTGCCCTCGTGGCTCGGAGAGTGTCGTGTATGCGCTGATGGCGGGCTTCTCCAACCTTGGCCAGACCACCGCGGCGTCCCTCGGTGCGATCATCATGGAGTACGGCCTGCCTGTGTTCAAGACCCAGGATGACGGGTCTCGCTGC
```

```
                            1801      1810      1820      1830      1840      1850      1860      1870      1880      1890      1900      1910      1920      1930      1940      1950      1960      1970      1980      1990      2000
                              |--------+---------+---------+---------+---------+---------+---------+---------+---------+---------+---------+---------+---------+---------+---------+---------+---------+---------+---------+---------|
WT                            AACTACGACAACCTGCCGCTGCTGCTGTTCCTGTGCAGCATGTGCACGCCGCTGCTGGTGATTCCGCTGAGCATGATACTGCTTCCGAAGGCGCGCATCTGCGACGATATCGACGTTGACGGCAAGGTGGTGCGCCAGGCCGTGGATAAGCAGGTCGCAGCTGCTCCGCTGTCGAGCTCCGACTCGGACGCGGTGATGGC
Mutant B recombinant type 1   --------------------------------------------------------------------------------------------------------------------------------------------------------------------------------------------------------
Mutant B recombinant type 2   --------------------------------------------------------------------------------------------------------------------------------------------------------------------------------------------------------
Mutant G mutation             AACTACGACAACCTGCCGCTGCTGCTGTTCCTGTGCAGCATGTGCACGCCGCTGCTGGTGATTCCGCTGAGCATGATACTGCTTCCGAAGGCGCGCATCTGCGACGATATCGACGTTGACGGCAAGGTGGTGCGCCAGGCCGTGGATAAGCAGGTCGCAGCTGCTCCGCTGTCGAGCTCCGACTCGGACGCGGTGATGGC
Mutant G recombinant          --------------------------------------------------------------------------------------------------------------------------------------------------------------------------------------------------------
Mutant E mutation             AACTACGACAACCTGCCGCTGCTGCTGTTCCTGTGCAGCATGTGCACGCCGCTGCTGGTGATTCCGCTGAGCATGATACTGCTTCCGAAGGCGCGCATCTGCGACGATATCGACGTTGACGGCAAGGTGGTGCGCCAGGCCGTGGATAAGCAGGTCGCAGCTGCTCCGCTGTCGAGCTCCGACTCGGACGCGGTGATGGC
Mutant E gene conversion      AACTACGACAACCTGCCGCTGCTGCTGTTCCTGTGCAGCATGTGCACGCCGCTGCTGGTGATTCCGCTGAGCATGATACTGCTTCCGAAGGCGCGCATCTGCGACGATATCGACGTTGACGGCAAGGTGGTGCGCCAGGCCGTGGATAAGCAGGTCGCAGCTGCTCCGCTGTCGAGCTCCGACTCGGACGCGGTGATGGC

                            2001      2010      2020      2030      2040      2050      2060      2070  2076      (UTR)     1         10        20        30        40        50        60        70        80        90        100       110
                              |--------+---------+---------+---------+---------+---------+---------+-----|      (UTR)     |--------+---------+---------+---------+---------+---------+---------+---------+---------+---------+---------|
WT                            TGCCGAGCCGCTTCATGGGAACAAGGCGGATGAGCGCGCGCCGGTGCAGAGCGGGCGCGGGCAGACGCAGAAATAG      (UTR)     ATGTCCTACAAGGAGGTCGCTCCCAAGCGCGAGAAGGATGCCGCCTCAGACGCCGCGGGTATAGCGGCGGTGCCTGAGGCGGCTGCCGCGGGAAATGATGACAAGTACAT
Mutant B recombinant type 1   --------------------------------------------------------------------------------------------------------------------------------------------------------------------------------------------------------
Mutant B recombinant type 2   --------------------------------------------------------------------------------------------------------------------------------------------------------------------------------------------------------
Mutant G mutation             TGCCGAGCCGCTTCATGGGAACAAGGCGGATGAGCGCGCGCCGGTGCAGAGCGGGCGCGGGCAGACGCAGAAATAG      (UTR)     ATGTCCTACAAGGAGGTCGCTCCCAAGCGCGAGAAGGATGCCGCCTCAGACGCCGCGGGTATAGCGGCGGTGCCTGAGGCGGCTGCCGCGGGAAATGATGACAAGTACAT
Mutant G recombinant          --------------------------------------------------------------------------------------------------------------------------------------------------------------------------------------------------------
Mutant E mutation             TGCCGAGCCGCTTCATGGGAACAAGGCGGATGAGCGCGCGCCGGTGCAGAGCGGGCGCGGGCAGACGCAGAAATAG      (UTR)     ATGTCCTACAAGGAGGTCGCTCCCAAGCGCGAGAAGGATGCCGCCTCAGACGCCGCGGGTATAGCGGCGGTGCCTGAGGCGGCTGCCGCGGGAAATGATGACAAGTACAT
Mutant E gene conversion      TGCCGAGCCGCTTCATGGGAACAAGGCGGATGAGCGCGCGCCGGTGCAGAGCGGGCGCGGGCAGACGCAGAAATAG      (UTR)     ATGTCCTACAAGGAGGTCGCTCCCAAGCGCGAGAAGGATGCCGCCTCAGACGCCGCGGGTATAGCGGCGGTGCCTGAGGCGGCTGCCGCGGGAAATGATGACAAGTACAT

                            111       120       130       140       150       160       170       180       190       200       210       220       230       240       250       260       270       280       290       300       310
                              |--------+---------+---------+---------+---------+---------+---------+---------+---------+---------+---------+---------+---------+---------+---------+---------+---------+---------+---------+---------|
WT                            CCACCCCGAGGCAGCATCCCTGTTCGCCAGGTGCCCGTGTGCTCGCCGCGTCCCCGTGTTTGGTGACGCTGTCGAGGGCTACGGGCTCAAGTTCATCATCGCCCTAGGCGCTAGCAACCTGCTCTGCAAGGGTATCGCGGATCAGATTCTTACCGGTCAGACGTACGCCATGATGATTGATCGCTACGGCATCGACGTGG
Mutant B recombinant type 1   --------------------------------------------------------------------------------------------------------------------------------------------------------------------------------------------------------
Mutant B recombinant type 2   --------------------------------------------------------------------------------------------------------------------------------------------------------------------------------------------------------
Mutant G mutation             CCACCCCGAGGCAGCATCCCTGTTCGCCAGGTGCCCGTGTGCTCGCCGCGTCCCCGTGTTTGGTGACGCTGTCGAGGGCTACGGGCTCAAGTTCATCATCGCCCTAGGCGCTAGCAACCTGCTCTGCAAGGGTATCGCGGATCAGATTCTTACCGGTCAGACGTACGCCATGATGATTGATCGCTACGGCATCGACGTGG
Mutant G recombinant          --------------------------------------------------------------------------------------------------------------------------------------------------------------------------------------------------------
Mutant E mutation             CCACCCCGAGGCAGCATCCCTGTTCGCCAGGTGCCCGTGTGCTCGCCGCGTCCCCGTGTTTGGTGACGCTGTCGAGGGCTACGGGCTCAAGTTCATCATCGCCCTAGGCGCTAGCAACCTGCTCTGCAAGGGTATCGCGGATCAGATTCTTACCGGTCAGACGTACGCCATGATGATTGATCGCTACGGCATCGACGTGG
Mutant E gene conversion      CCACCCCGAGGCAGCATCCCTGTTCGCCAGGTGCCCGTGTGCTCGCCGCGTCCCCGTGTTTGGTGACGCTGTCGAGGGCTACGGGCTCAAGTTCATCATCGCCCTAGGCGCTAGCAACCTGCTCTGCAAGGGTATCGCGGATCAGATTCTTACCGGTCAGACGTACGCCATGATGATTGATCGCTACGGCATCGACGTGG

                            311       320       330       340       350       360       370       380       390       400       410       420       430       440       450       460       470       480       490       500       510
                              |--------+---------+---------+---------+---------+---------+---------+---------+---------+---------+---------+---------+---------+---------+---------+---------+---------+---------+---------+---------|
WT                            CCCGCTACCAGCGCCTGTCTTCGCTTTCGTCCATGGGGTGGTCCATCAAGGCCTTCACCGCGATGCTCTGCGACGGCTTCGCCTTCCTCGGCTACACGAAGCGCTGGTACATGTTCATCTCCTGCGTCGGCGGTGGTGTTTTCGCCCTCCTGTACGGCCTGCTGCCCGCGAAGGAGGCGTCGGCTGATGTGGCGTGTGCC
Mutant B recombinant type 1   --------------------------------------------------------------------------------------------------------------------------------------------------------------------------------------------------------
Mutant B recombinant type 2   --------------------------------------------------------------------------------------------------------------------------------------------------------------------------------------------------------
Mutant G mutation             CCCGCTACCAGCGCCTGTCTTCGCTTTCGTCCATGGGGTGGTCCATCAAGGCCTTCACCGCGATGCTCTGCGACGGCTTCGCCTTCCTCGGCTACACGAAGCGCTGGTACATGTTCATCTCCTGCGTCGGCGGTGGTGTTTTCGCCCTCCTGTACGGCCTGCTGCCCGCGAAGGAGGCGTCGGCTGATGTGGCGTGTGCC
Mutant G recombinant          --------------------TCGCTTTCGTCCATGGGGTGGTCCATCAAGGCCTTCACCGCGATGCTCTGCGACGGCTTCGCCTTCCTCGGCTACACGAAGCGCTGGTACATGTTCATCTCCTGCGTCGGCGGTGGTGTTTTCGCCCTCCTGTACGGCCTGCTGCCCGCGAAGGAGGCGTCGGCTGATGTGGCGTGTGCC
Mutant E mutation             CCCGCTACCAGCGCCTGTCTTCGCTTTCGTCCATGGGGTGGTCCATCAAGGCCTTCACCGCGATGCTCTGCGACGGCTTCGCCTTCCTCGGCTACACGAAGCGCTGGTACATGTTCATCTCCTGCGTCGGCGGTGGTGTTTTCGCCCTCCTGTACGGCCTGCTGCCCGCGAAGGAGGCGTCGGCTGATGTGGCGTGTGCC
Mutant E gene conversion      CCCGCTACCAGCGCCTGTCTTCGCTTTCGTCCATGGGGTGGTCCATCAAGGCCTTCACCGCGATGCTCTGCGACGGCTTCGCCTTCCTCGGCTACACGAAGCGCTGGTACATGTTCATCTCCTGCGTCGGCGGTGGTGTTTTCGCCCTCCTGTACGGCCTGCTGCCCGCGAAGGAGGCGTCGGCTGATGTGGCGTGTGCC

                            511       520       530       540       550       560       570       580       590       600       610       620       630       640       650       660       670       680       690       700       710
                              |--------+---------+---------+---------+---------+---------+---------+---------+---------+---------+---------+---------+---------+---------+---------+---------+---------+---------+---------+---------|
WT                            TTCATCTTCCTGTCGTGCTGGGGCAAGGCCAACGTGGATATCCTGTCCCAGGGCCATTACAGTCGACTGATGCGCGAGAACCCGAAGCCTGGCCCGGCGCTGGTGAGCTGGATCTGGCTCTGGATCATGACCGGCTCGCTCATCGCGACTGTGATGAACGGCCCGCTCGCGGATGCCGGGAAGCCGCAGATCAGCATCTT
Mutant B recombinant type 1   --------------------------------------------------------------------------------------------------------------------------------------------------------------------------------------------------------
Mutant B recombinant type 2   --------------------------------------------------------------------------------------------------------------------------------------------------------------------------------------------------------
Mutant G mutation             TTCATCTTCCTGTCGTGCTGGGGCAAGGCCAACGTGGATATCCTGTCCCAGAGCCATTACAGTCGACTGATGCGCGAGAACCCGAAGCCTGGCCCGGCGCTGGTGAGCTGGATCTGGCTCTGGATCATGACCGGCTCGCTCATCGCGACTGTGATGAACGGCCCGCTCGCGGATGCCGGGAAGCCGCAGATCAGCATCTT
Mutant G recombinant          TTCATCTTCCTGTCGTGCTGGGGCAAGGCCAACGTGGATATCCTGTCCCAGGGCCATTACAGTCGACTGATGCGCGAGAACCCGAAGCCTGGCCCGGCGCTGGTGAGCTGGATCTGGCTCTGGATCATGACCGGCTCGCTCATCGCGACTGTGATGAACGGCCCGCTCGCGGATGCCGGGAAGCCGCAGATCAGCATCTT
Mutant E mutation             TTCATCTTCCTGTCGTGCTGGGGCAAGGCCAACGTGGATATCCTGTCCCAGGGCCATTACAGTCGACTGATGCGCGAGAACCCGAAGCCTGGCCCGGCGCTGGTGAGCTGGATCTGGCTCTGGATCATGACCGGCTCGCTCATCGCGACTGTGATGAACGGCCCGCTCGCGGATGCCGGGAAGCCGCAGATCAGCATCTT
Mutant E gene conversion      TTCATCTTCCTGTCGTGCTGGGGCAAGGCCAACGTGGATATCCTGTCCCAGGGCCATTACAGTCGACTGATGCGCGAGAACCCGAAGCCTGGCCCGGCGCTGGTGAGCTGGATCTGGCTCTGGATCATGACCGGCTCGCTCATCGCGACTGTGATGAACGGCCCGCTCGCGGATGCCGGGAAGCCGCAGATCAGCATCTT

                            711       720       730       740       750       760       770       780       790       800       810       820       830       840       850       860       870       880       890       900       910
                              |--------+---------+---------+---------+---------+---------+---------+---------+---------+---------+---------+---------+---------+---------+---------+---------+---------+---------+---------+---------|
WT                            CGTGTCTGCCGCGCTGCAGGCCATCACCTGCGTCTTCTACCTGTTCAACTGGTACGGGGAGAAGAAGAACCGCGTGCTGCGCTCCGAGGACGCGCTGTTTATTCTGGAGGAGACCCGCAAGGAGCGTGAGCGCCTGGGTCTCGAGGGGGTGTACGACGGCACGGCGGGTGCGCAGCATGGTGGTGCGGCGAAGGGGAAGA
Mutant B recombinant type 1   --------------------------------------------------------------------------------------------------------------------------------------------------------------------------------------------------------
Mutant B recombinant type 2   --------------------------------------------------------------------------------------------------------------------------------------------------------------------------------------------------------
Mutant G mutation             CGTGTCTGCCGCGCTGCAGGCCATCACCTGCGTCTTCTACCTGTTCAACTGGTACGGGGAGAAGAAGAACCGCGTGCTGCGCTCCGAGGACGCGCTGTTTATTCTGGAGGAGACCCGCAAGGAGCGTGAGCGCCTGGGTCTCGAGGGGGTGTACGACGGCACGGCGGGTGCGCAGCATGGTGGTGCGGCGAAGGGGAAGA
Mutant G recombinant          CGTGTCTGCCGCGCTGCAGGCCATCACCTGCGTCTTCTACCTGTTCAACTGGTACGGGGAGAAGAAGAACCGCGTGCTGCGCTCCGAGGACGCGCTGTTTATTCTGGAGGAGACCCGCAAGGAGCGTGAGCGCCTGGGTCTCGAGGGGGTGTACGACGGCACGGCGGGTGCGCAGCATGGTGGTGCGGCGAAGGGGAAGA
Mutant E mutation             CGTGTCTGCCGCGCTGCAGGCCATCACCTGCGTCTTCTACCTGTTCAACTGGTACGGGGAGAAGAAGAACCGCGTGCTGCGCTCCGAGGACGCGCTGTTTATTCTGGAGGAGACCCGCAAGGAGCGTGAGCGCCTGGGTCTCGAGGGGGTGTACGACGGCACGGCGGGTGCGCAGCATGGTGGTGCGGCGAAGGGGAAGA
Mutant E gene conversion      CGTGTCTGCCGCGCTGCAGGCCATCACCTGCGTCTTCTACCTGTTCAACTGGTACGGGGAGAAGAAGAACCGCGTGCTGCGCTCCGAGGACGCGCTGTTTATTCTGGAGGAGACCCGCAAGGAGCGTGAGCGCCTGGGTCTCGAGGGGGTGTACGACGGCACGGCGGGTGCGCAGCATGGTGGTGCGGCGAAGGGGAAGA

                            911       920       930       940       950       960       970       980       990       1000      1010      1020      1030      1040      1050      1060      1070      1080      1090      1100      1110
                              |--------+---------+---------+---------+---------+---------+---------+---------+---------+---------+---------+---------+---------+---------+---------+---------+---------+---------+---------+---------|
WT                            AGAGCCCGCAGCACTCGCACTCGGATGAGGACGTGGAGGGTGCCGTACGGGACGCCCTCAACGATGGTCAGCGCGACAACGGTGAACTTGTGCAGGACGTCTACGACGACGCGTATGACGACGGCGAGGGGGTGGCCGAGGGCGATGTGTACTACGGCAAGCCGCCGGTGCCGTGCCTGTTCGGGCTGTTCGAGGCAAAC
Mutant B recombinant type 1   --------------------------------------------------------------------------------------------------------------------------------------------------------------------------------------------------------
Mutant B recombinant type 2   --------------------------------------------------------------------------------------------------------------------------------------------------------------------------------------------------------
Mutant G mutation             AGAGCCCGCAGCACTCGCACTCGGATGAGGACGTGGAGGGTGCCGTACGGGACGCCCTCAACGATGGTCAGCGCGACAACGGTGAACTTGTGCAGGACGTCTACGACGACGCGTATGACGACGGCGAGGGGGTGGCCGAGGGCGATGTGTACTACGGCAAGCCGCCGGTGCCGTGCCTGTTCGGGCTGTTCGAGGCAAAC
Mutant G recombinant          AGAGCCCGCAGCACTCGCACTCGGATGAGGACGTGGAGGGTGCCGTACGGGACGCCCTCAACGATGGTCAGCGCGACAACGGTGAACTTGTGCAGGACGTCTACGACGACGCGTATGACGACGGCGAGGGGGTGGCCGAGGGCGATGTGTACTACGGCAAGCCGCCGGTGCCGTGCCTGTTCGGGCTGTTCGAGGCAAAC
Mutant E mutation             AGAGCCCGCAGCACTCGCACTCGGATGAGGACGTGGAGGGTGCCGTACGGGACGCCCTCAACGATGGTCAGCGCGACAACGGTGAACTTGTGCAGGACGTCTACGACGACGCGTATGACGACGGCGAGGGGGTGGCCGAGGGCGATGTGTACTACGGCAAGCCGCCGGTGCCGTGCCTGTTCGGGCTGTTCGAGGCAAAC
Mutant E gene conversion      AGAGCCCGCAGCACTCGCACTCGGATGAGGACGTGGAGGGTGCCGTACGGGACGCCCTCAACGATGGTCAGCGCGACAACGGTGAACTTGTGCAGGACGTCTACGACGACGCGTATGACGACGGCGAGGGGGTGGCCGAGGGCGATGTGTACTACGGCAAGCCGCCGGTGCCGTGCCTGTTCGGGCTGTTCGAGGCAAAC

                            1111      1120      1130      1140      1150      1160      1170      1180      1190      1200      1210      1220      1230      1240      1250      1260      1270      1280      1290      1300      1310
                              |--------+---------+---------+---------+---------+---------+---------+---------+---------+---------+---------+---------+---------+---------+---------+---------+---------+---------+---------+---------|
WT                            ACGGAGGTGATTTCGAAGAACTGGAAGATCTTCGTGTACAGCGTCGTCATGACCTGTGCTGTGATCACGATGCTGTGTGCCAACATGCTGGCCGACACGCTGGGCCTCCTGGTTGCGTGCGTCGTTGTGTCGACCATCTGCTGTACCACGTCCTTCTGGGCCCTGCCGCTGGTGATTGCGAAGGCCAACGTGTTTGCATA
Mutant B recombinant type 1   --------------------------------------------------------------------------------------------------------------------------------------------------------------------------------------------------------
Mutant B recombinant type 2   --------------------------------------------------------------------------------------------------------------------------------------------------------------------------------------------------------
Mutant G mutation             ACGGAGGTGATTTCGAAGAACTGGAAGATCTTCGTGTACAGCGTCGTCATGACCTGTGCTGTGATCACGATGCTGTGTGCCAACATGCTGGCCGACACGCTGGGCCTCCTGGTTGCGTGCGTCGTTGTGTCGACCATCTGCTGTACCACGTCCTTCTGGGCCCTGCCGCTGGTGATTGCGAAGGCCAACGTGTTTGCATA
Mutant G recombinant          ACGGAGGTGATTTCGAAGAACTGGAAGATCTTCGTGTACAGCGTCGTCATGACCTGTGCTGTGATCACGATGCTGTGTGCCAACATGCTGGCCGACACGCTGGGCCTCCTGGTTGCGTGCGTCGTTGTGTCGACCATCTGCTGTACCACGTCCTTCTGGGCCCTGCCGCTGGTGATTGCGAAGGCCAACGTGTTTGCATA
Mutant E mutation             ACGGAGGTGATTTCGAAGAACTGGAAGATCTTCGTGTACAGCGTCGTCATGACCTGTGCTGTGATCACGATGCTGTGTGCCAACATGCTGGCCGACACGCTGGGCCTCCTGGTTGCGTGCGTCGTTGTGTCGACCATCTGCTGTACCACGTCCTTCTGGGCCCTGCCGCTGGTGATTGCGAAGGCCAACGTGTTTGCATA
Mutant E gene conversion      ACGGAGGTGATTTCGAAGAACTGGAAGATCTTCGTGTACAGCGTCGTCATGACCTGTGCTGTGATCACGATGCTGTGTGCCAACATGCTGGCCGACACGCTGGGCCTCCTGGTTGCGTGCGTCGTTGTGTCGACCATCTGCTGTACCACGTCCTTCTGGGCCCTGCCGCTGGTGATTGCGAAGGCCAACGTGTTTGCATA

                            1311      1320      1330      1340      1350      1360      1370      1380      1390      1400      1410      1420      1430      1440      1450      1460      1470      1480      1490      1500      1510
                              |--------+---------+---------+---------+---------+---------+---------+---------+---------+---------+---------+---------+---------+---------+---------+---------+---------+---------+---------+---------|
WT                            CCTGGATAAGGCTGTTTCCATCCGTGTGGGCGGTCCCCTAAATGCGTTCTACTTGAACACCTACCAGTGCCCTGGCAACCTGCCGAACTTCACCTACACCTTCTACAACACGGTGGCGGGCGTCATCAACACCATCGTTGGTATGATTACCGTGACGCTGTTCAACTTCCTGTTCGCGAAGCATGGCTACCGCCTCACCT
Mutant B recombinant type 1   --------------------------------------------------------------------------------------------------------------------------------------------------------------------------------------------------------
Mutant B recombinant type 2   --------------------------------------------------------------------------------------------------------------------------------------------------------------------------------------------------------
Mutant G mutation             CCTGGATAAGGCTGTTTCCATCCGTGTGGGCGGTCCCCTAAATGCGTTCTACTTGAACACCTACCAGTGCCCTGGCAACCTGCCGAACTTCACCTACACCTTCTACAACACGGTGGCGGGCGTCATCAACACCATCGTTGGTATGATTACCGTGACGCTGTTCAACTTCCTGTTCGCGAAGCATGGCTACCGCCTCACCT
Mutant G recombinant          CCTGGATAAGGCTGTTTCCATCCGTGTGGGCGGTCCCCTAAATGCGTTCTACTTGAACACCTACCAGTGCCCTGGCAACCTGCCGAACTTCACCTACACCTTCTACAACACGGTGGCGGGCGTCATCAACACCATCGTTGGTATGATTACCGTGACGCTGTTCAACTTCCTGTTCGCGAAGCATGGCTACCGCCTCACCT
Mutant E mutation             CCTGGATAAGGCTGTTTCCATCCGTGTGGGCGGTCCCCTAAATGCGTTCTACTTGAACACCTACCAGTGCCCTGGCAACCTGCCGAACTTCACCTACACCTTCTACAACACGGTGGCGGGCGTCATCAACACCATCGTTGGTATGATTACCGTGACGCTGTTCAACTTCCTGTTCGCGAAGCATGGCTACCGCCTCACCT
Mutant E gene conversion      CCTGGATAAGGCTGTTTCCATCCGTGTGGGCGGTCCCCTAAATGCGTTCTACTTGAACACCTACCAGTGCCCTGGCAACCTGCCGAACTTCACCTACACCTTCTACAACACGGTGGCGGGCGTCATCAACACCATCGTTGGTATGATTACCGTGACGCTGTTCAACTTCCTGTTCGCGAAGCATGGCTACCGCCTCACCT
```

```
                            1511    1520      1530      1540      1550      1560      1570      1580      1590      1600      1610      1620      1630      1640      1650      1660      1670      1680      1690      1700      1710
WT                          TCATTGTGACGACAATCATGCAGGTTATGGGTGGTGTGTTCGACATCATCATTGTGAAGCGGTGGAACCTGTACATTGGCATCCCTGACCACGCCATGTACATCTGGGGTGATGCTGTTGTGGGTGAGCTCGTGTACATGCTTGGCTTTATGCCGCAGATCGTGCTGCTGTCTCGCCTGTGCCCTCGTGGCTCGGAGAGT
Mutant B recombinant type 1 --------------------------------------------------------------------------------------------------------------------------------------------------------------------------------------------------------
Mutant B recombinant type 2 --------------------------------------------------------------------------------------------------------------------------------------------------------------------------------------------------------
Mutant G mutation           TCATTGTGACGACAATCATGCAGGTTATGGGTGGTGTGTTCGACATCATCATTGTGAAGCGGTGGAACCTGTACATTGGCATCCCTGACCACGCCATGTACATCTGGGGTGATGCTGTTGTGGGTGAGCTCGTGTACATGCTTGGCTTTATGCCGCAGATCGTGCTGCTGTCTCGCCTGTGCCCTCGTGGCTCGGAGAGT
Mutant G recombinant        TCATTGTGACGACAATCATGCAGGTTATGGGTGGTGTGTTCGACATCATCATTGTGAAGCGGTGGAACCTGTACATTGGCATCCCTGACCACGCCATGTACATCTGGGGTGATGCTGTTGTGGGTGAGCTCGTGTACATGCTTGGCTTTATGCCGCAGATCGTGCTGCTGTCTCGCCTGTGCCCTCGTGGCTCGGAGAGT
Mutant E mutation           TCATTGTGACGACAATCATGCAGGTTATGGGTGGTGTGTTCGACATCATCATTGTGAAGCGGTGGAACCTGTACATTGGCATCCCTGACCACGCCATGTACATCTGGGGTGATGCTGTTGTGGGTGAGCTCGTGTACATGCTTGGCTTTATGCCGCAGATCGTGCTGCTGTCTCGCCTGTGCCCTCGTGGCTCGGAGAGT
Mutant E gene conversion    TCATTGTGACGACAATCATGCAGGTTATGGGTGGTGTGTTCGACATCATCATTGTGAAGCGGTGGAACCTGTACATTGGCATCCCTGACCACGCCATGTACATCTGGGGTGATGCTGTTGTGGGTGAGCTCGTGTACATGCTTGGCTTTATGCCGCAGATCGTGCTGCTGTCTCGCCTGTGCCCTCGTGGCTCGGAGAGT

                            1711    1720      1730      1740      1750      1760      1770      1780      1790      1800      1810      1820      1830      1840      1850      1860      1870      1880      1890      1900      1910
WT                          GTCGTGTATGCGCTGATGGCGGGCTTCGCCCGCCTCGGCCGAACGACCGCGTCGTCCCTCGGTGCCATCCTTCTGGAGTACGGCCTGCCTGTGTTCAAGACCCAGGATGACGGGTCTCGCTGCAACTACGACAACCTGCCGCTGCTGTTGTTCGTGACCAGCATGTGCACGCCGCTGCTGGCGATTCCGCTGAGCATGAT
Mutant B recombinant type 1 --------------------------------------------------------------------------------------------------------------------------------------------------------------------------------------------------------
Mutant B recombinant type 2 --------------------------------------------------------------------------------------------------------------------------------------------------------------------------------------------------------
Mutant G mutation           GTCGTGTATGCGCTGATGGCGGGCTTCGCCCGCCTCGGCCGAACGACCGCGTCGTCCCTCGGTGCCATCCTTCTGGAGTACGGCCTGCCTGTGTTCAAGACCCAGGATGACGGGTCTCGCTGCAACTACGACAACCTGCCGCTGCTGTTGTTCGTGACCAGCATGTGCACGCCGCTGCTGGCGATTCCGCTGAGCATGAT
Mutant G recombinant        GTCGTGTATGCGCTGATGGCGGGCTTCGCCCGCCTCGGCCGAACGACCGCGTCGTCCCTCGGTGCCATCCTTCTGGAGTACGGCCTGCCTGTGTTCAAGACCCAGGATGACGGGTCTCGCTGCAACTACGACAACCTGCCGCTGCTGTTGTTCGTGACCAGCATGTGCACGCCGCTGCTGGCGATTCCGCTGAGCATGAT
Mutant E mutation           GTCGTGTATGCGCTGATGACGGGCTTCGCCCGCCTCGGCCGAACGACCGCGTCGTCCCTCGGTGCCATCCTTCTGGAGTACGGCCTGCCTGTGTTCAAGACCCAGGATGACGGGTCTCGCTGCAACTACGACAACCTGCCGCTGCTGTTGTTCGTGACCAGCATGTGCACGCCGCTGCTGGCGATTCCGCTGAGCATGAT
Mutant E gene conversion    GTCGTGTATGCGCTGATGGCGGGCTTCGCGAGTCTTGGCCAGACCACCGCGTCGTCCCTCGGTGCGATCATCATGGAGTACGGCCTGCCTGTGTTCAAGACCCAGGATGACGGGTCTCGCTGCAACTACGACAACCTGCCGCTGCTGTTGTTCGTGACCAGCATGTGCACGCCGCTGCTGGCGATTCCGCTGAGCATGAT

                            1911    1920      1930      1940      1950      1960      1970      1980      1990      2000      2010      2020      2030      2040      2050      2060      2070      2080      2090      2100      2110
WT                          ACTGCTTCCGAAGGCGCGCATCTGCGACGATATCGACGTTGACGGCAAGGTGGTGCGCCAGGCCGTGGATAAGCAGGTCGCAGCTGCTCCGCTGTCGAGCTCCGACTCGGACGCGGTGATGGCTGCCGAGCCGCTTCATGGGAACAAGGCGGATGAGCGCGAGGCAGCGCGCGGGGAGACGGTGCAGGTAAACCCGCAG
Mutant B recombinant type 1 --------------------------------------------------------------------------------------------------------------------------------------------------------------------------------------------------------
Mutant B recombinant type 2 --------------------------------------------------------------------------------------------------------------------------------------------------------------------------------------------------------
Mutant G mutation           ACTGCTTCCGAAGGCGCGCATCTGCGACGATATCGACGTTGACGGCAAGGTGGTGCGCCAGGCCGTGGATAAGCAGGTCGCAGCTGCTCCGCTGTCGAGCTCCGACTCGGACGCGGTGATGGCTGCCGAGCCGCTTCATGGGAACAAGGCGGATGAGCGCGAGGCAGCGCGCGGGGAGACGGTGCAGGTAAACCCGCAG
Mutant G recombinant        ACTGCTTCCGAAGGCGCGCATCTGCGACGATATCGACGTTGACGGCAAGGTGGTGCGCCAGGCCGTGGATAAGCAGGTCGCAGCTGCTCCGCTGTCGAGCTCCGACTCGGACGCGGTGATGGCTGCCGAGCCGCTTCATGGGAACAAGGCGGATGAGCGCGAGGCAGCGCGCGGGGAGACGGTGCAGGTAAACCCGCAG
Mutant E mutation           ACTGCTTCCGAAGGCGCGCATCTGCGACGATATCGACGTTGACGGCAAGGTGGTGCGCCAGGCCGTGGATAAGCAGGTCGCAGCTGCTCCGCTGTCGAGCTCCGACTCGGACGCGGTGATGGCTGCCGAGCCGCTTCATGGGAACAAGGCGGATGAGCGCGAGGCAGCGCGCGGGGAGACGGTGCAGGTAAACCCGCAG
Mutant E gene conversion    ACTGCTTCCGAAGGCGCGCATCTGCGACGATATCGACGTTGACGGCAAGGTGGTGCGCCAGGCCGTGGATAAGCAGGTCGCAGCTGCTCCGCTGTCGAGCTCCGACTCGGACGCGGTGATGGCTGCCGAGCCGCTTCATGGGAACAAGGCGGATGAGCGCGAGGCAGCGCGCGGGGAGACGGTGCAGGTAAACCCGCAG

                            2111    2120  2127  (UTR)   1       10        20        30        40        50        60        70        80        90        100       110       120       130       140       150       160       170
WT                          GCGGTTCAGAGAAATAG   (UTR)   ATGCTGGAGGCTCCGACGCATCCCCACACCGCTAAAGCATCCCACACACAACCACGGCATCCGGAACAATCTGCCACACTCCTTGCGCAACAAATTACCACGATGTCCCGCAAGGAGGTCGCTCCCAAGCGCGAGAAGGATGCCGCCTCAGACGCCGCGGGTACAGCGGC
Mutant B recombinant type 1 -----------------   (UTR)   ----------------------------------------------------------------------------------------------------------------------------------------------------------------------
Mutant B recombinant type 2 -----------------   (UTR)   ----------------------------------------------------------------------------------------------------------------------------------------------------------------------
Mutant G mutation           GCGGTTCAGAGAAATAG   (UTR)   TACATCCACCCCGAGGCAGCATCCCTGTTCGCCAGGTGCCCGTGGGCTCGCCGCGTCCCCGTGTTTGGTGACGCTGTCGAGGGCTACGGGCTCAAGGTCATCGTCACTCTTGGTACGAGCTACCTGCTCTGCAAGGGTATCGCGGATCAGATTCTATATGATCAGACGTA
Mutant G recombinant        GCGGTTCAGAGAAATAG   (UTR)   TACATCCACCCCGAGGCAGCATCCCTGTTCGCCAGGTGCCCGTGGGCTCGCCGCGTCCCCGTGTTTGGTGACGCTGTCGAGGGCTACGGGCTCAAGGTCATCGTCACTCTTGGTACGAGCTACCTGCTCTGCAAGGGTATCGCGGATCAGATTCTATATGATCAGACGTA
Mutant E mutation           GCGGTTCAGAGAAATAG   (UTR)   TACATCCACCCCGAGGCAGCATCCCTGTTCGCCAGGTGCCCGTGGGCTCGCCGCGTCCCCGTGTTTGGTGACGCTGTCGAGGGCTACGGGCTCAAGGTCATCGTCACTCTTGGTACGAGCTACCTGCTCTGCAAGGGTATCGCGGATCAGATTCTATATGATCAGACGTA
Mutant E gene conversion    GCGGTTCAGAGAAATAG   (UTR)   TACATCCACCCCGAGGCAGCATCCCTGTTCGCCAGGTGCCCGTGGGCTCGCCGCGTCCCCGTGTTTGGTGACGCTGTCGAGGGCTACGGGCTCAAGGTCATCGTCACTCTTGGTACGAGCTACCTGCTCTGCAAGGGTATCGCGGATCAGATTCTATATGATCAGACGTA

                            171     180       190       200       210       220       230       240       250       260       270       280       290       300       310       320       330       340       350       360       370
WT                          GGTGCCTGAGGCGGCTGCCGCGGGAAATGATGACAAGTACATCCACCCCGAGGCAGCATCCCTGTTCGCCAGGTGCCCGTGGGCTCGCCGCGTCCCCGTGTTTGGTGACGCTGTCGAGGGCTACGGGCTCAAGGTCATCGTCACTCTTGGTACGAGCTACCTGCTCTGCAAGGGTATCGCGGATCAGATTCTATATGATC
Mutant B recombinant type 1 --------------------------------------------------------------------------------------------------------------------------------------------------------------------------------------------------------
Mutant B recombinant type 2 --------------------------------------------------------------------------------------------------------------------------------------------------------------------------------------------------------
Mutant G mutation           GGTGCCTGAGGCGGCTGCCGCGGGAAATGATGACAAGTACATCCACCCCGAGGCAGCATCCCTGTTCGCCAGGTGCCCGTGGGCTCGCCGCGTCCCCGTGTTTGGTGACGCTGTCGAGGGCTACGGGCTCAAGGTCATCGTCACTCTTGGTACGAGCTACCTGCTCTGCAAGGGTATCGCGGATCAGATTCTATATGATC
Mutant G recombinant        GGTGCCTGAGGCGGCTGCCGCGGGAAATGATGACAAGTACATCCACCCCGAGGCAGCATCCCTGTTCGCCAGGTGCCCGTGGGCTCGCCGCGTCCCCGTGTTTGGTGACGCTGTCGAGGGCTACGGGCTCAAGGTCATCGTCACTCTTGGTACGAGCTACCTGCTCTGCAAGGGTATCGCGGATCAGATTCTATATGATC
Mutant E mutation           GGTGCCTGAGGCGGCTGCCGCGGGAAATGATGACAAGTACATCCACCCCGAGGCAGCATCCCTGTTCGCCAGGTGCCCGTGGGCTCGCCGCGTCCCCGTGTTTGGTGACGCTGTCGAGGGCTACGGGCTCAAGGTCATCGTCACTCTTGGTACGAGCTACCTGCTCTGCAAGGGTATCGCGGATCAGATTCTATATGATC
Mutant E gene conversion    GGTGCCTGAGGCGGCTGCCGCGGGAAATGATGACAAGTACATCCACCCCGAGGCAGCATCCCTGTTCGCCAGGTGCCCGTGGGCTCGCCGCGTCCCCGTGTTTGGTGACGCTGTCGAGGGCTACGGGCTCAAGGTCATCGTCACTCTTGGTACGAGCTACCTGCTCTGCAAGGGTATCGCGGATCAGATTCTATATGATC

                            371     380       390       400       410       420       430       440       450       460       470       480       490       500       510       520       530       540       550       560       570
WT                          AGACGTACGCCATGATGATTGATCGCTACGGCATCGACGTGGCCCGCTACCAGCGCCTGTCTCCGATTTTGTTCATGGGGTGGTCCATCAAGGCCTTCACCGCGATGCTCTGCGACGGCTTCGCCTTCCTCGGCTACACGAAGCGCTGGTACATGTTCATCTCCTGCGTCGGCGGTGGTGCGTTCGCGCTGATCTACGGC
Mutant B recombinant type 1 ---------------------------------------------------------------------TGTTCATGGGGTGGTCCATCAAGGCCTTCACCGCGATGCTCTGCGACGGCTTCGCCTTCCTCGGCTACACGAAGCGCTGGTACATGTTCATCTCCTGCGTCGGCGGTGGTGCGTTCGCGCTGATCTACGGC
Mutant B recombinant type 2 -------------------------------------------------------------------------CATGGGGTGGTCCATCAAGGCCTTCACCGCGATGCTCTGCGACGGCTTCGCCTTCCTCGGCTACACGAAGCGCTGGTACATGTTCATCTCCTGCGTCGGCGGTGGTGCGTTCGCGCTGATCTACGGC
Mutant G mutation           AGACGTACGCCATGATGATTGATCGCTACGGCATCGACGTGGCCCGCTACCAGCGCCTGTCTCCGATTTTGTTCATGGGGTGGTCCATCAAGGCCTTCACCGCGATGCTCTGCGACGGCTTCGCCTTCCTCGGCTACACGAAGCGCTGGTACATGTTCATCTCCTGCGTCGGCGGTGGTGCGTTCGCGCTGATCTACGGC
Mutant G recombinant        AGACGTACGCCATGATGATTGATCGCTACGGCATCGACGTGGCCCGCTACCAGCGCCTGTCTCCGATTTTGTTCATGGGGTGGTCCATCAAGGCCTTCACCGCGATGCTCTGCGACGGCTTCGCCTTCCTCGGCTACACGAAGCGCTGGTACATGTTCATCTCCTGCGTCGGCGGTGGTGCGTTCGCGCTGATCTACGGC
Mutant E mutation           AGACGTACGCCATGATGATTGATCGCTACGGCATCGACGTGGCCCGCTACCAGCGCCTGTCTCCGATTTTGTTCATGGGGTGGTCCATCAAGGCCTTCACCGCGATGCTCTGCGACGGCTTCGCCTTCCTCGGCTACACGAAGCGCTGGTACATGTTCATCTCCTGCGTCGGCGGTGGTGCGTTCGCGCTGATCTACGGC
Mutant E gene conversion    AGACGTACGCCATGATGATTGATCGCTACGGCATCGACGTGGCCCGCTACCAGCGCCTGTCTCCGATTTTGTTCATGGGGTGGTCCATCAAGGCCTTCACCGCGATGCTCTGCGACGGCTTCGCCTTCCTCGGCTACACGAAGCGCTGGTACATGTTCATCTCCTGCGTCGGCGGTGGTGCGTTCGCGCTGATCTACGGC

                            571     580       590       600       610       620       630       640       650       660       670       680       690       700       710       720       730       740       750       760       770
WT                          CTGCTGCCCGCGAAGGAGGCGTCGGCTGATGTGGCGTGTGCCTTCATCTTCCTGTCGTGCTGGGGCAAGGCCAACGTGGATATCCTGTCCCAGGGCCATTACAGTCGACTGATGCGCGAGAACCCGAAGCCTGGCCCGGCGCTGGTGAGCTGGATCTGGCTCTGGATCATGACCGGCTCGCTCATCGCGACTGTGATGAA
Mutant B recombinant type 1 CTGCTGCCCGCGAAGGAGGCGTCGGCTGATGTGGCGTGTGCCTTCATCTTCCTGTCGTGCTGGGGCAAGGCCAACGTGGATATCCTGTCCCAGGGCCATTACAGTCGACTGATGCGCGAGAACCCGAAGCCTGGCCCGGCGCTGGTGAGCTGGATCTGGCTCTGGATCATGACCGGCTCGCTCATCGCGACTGTGATGAA
Mutant B recombinant type 2 CTGCTGCCCGCGAAGGAGGCGTCGGCTGATGTGGCGTGTGCCTTCATCTTCCTGTCGTGCTGGGGCAAGGCCAACGTGGATATCCTGTCCCAGGGCCATTACAGTCGACTGATGCGCGAGAACCCGAAGCCTGGCCCGGCGCTGGTGAGCTGGATCTGGCTCTGGATCATGACCGGCTCGCTCATCGCGACTGTGATGAA
Mutant G mutation           CTGCTGCCCGCGAAGGAGGCGTCGGCTGATGTGGCGTGTGCCTTCATCTTCCTGTCGTGCTGGGGCAAGGCCAACGTGGATATCCTGTCCCAGGGCCATTACAGTCGACTGATGCGCGAGAACCCGAAGCCTGGCCCGGCGCTGGTGAGCTGGATCTGGCTCTGGATCATGACCGGCTCGCTCATCGCGACTGTGATGAA
Mutant G recombinant        CTGCTGCCCGCGAAGGAGGCGTCGGCTGATGTGGCGTGTGCCTTCATCTTCCTGTCGTGCTGGGGCAAGGCCAACGTGGATATCCTGTCCCAGGGCCATTACAGTCGACTGATGCGCGAGAACCCGAAGCCTGGCCCGGCGCTGGTGAGCTGGATCTGGCTCTGGATCATGACCGGCTCGCTCATCGCGACTGTGATGAA
Mutant E mutation           CTGCTGCCCGCGAAGGAGGCGTCGGCTGATGTGGCGTGTGCCTTCATCTTCCTGTCGTGCTGGGGCAAGGCCAACGTGGATATCCTGTCCCAGGGCCATTACAGTCGACTGATGCGCGAGAACCCGAAGCCTGGCCCGGCGCTGGTGAGCTGGATCTGGCTCTGGATCATGACCGGCTCGCTCATCGCGACTGTGATGAA
Mutant E gene conversion    CTGCTGCCCGCGAAGGAGGCGTCGGCTGATGTGGCGTGTGCCTTCATCTTCCTGTCGTGCTGGGGCAAGGCCAACGTGGATATCCTGTCCCAGGGCCATTACAGTCGACTGATGCGCGAGAACCCGAAGCCTGGCCCGGCGCTGGTGAGCTGGATCTGGCTCTGGATCATGACCGGCTCGCTCATCGCGACTGTGATGAA

                            771     780       790       800       810       820       830       840       850       860       870       880       890       900       910       920       930       940       950       960       970
WT                          CGGCCCGCTCGCGGATGCCGGGAAGCCGCAGATCAGCATCTTCGTGTCTGCCGCGCTGCAGGCCATCACCTGCGTCTTCTACCTGTTCAACTGGTACGGGGAGAAGAAGAACCGCGTGCTGCGCTCCGAGGACGCGCTGTTTATTCTGGAGGAGACCCGCAAGGAGCGTGAGCGCCTGGGTCTCGAGGGGGTGTACGACG
Mutant B recombinant type 1 CGGCCCGCTCGCGGATGCCGGGAAGCCGCAGATCAGCATCTTCGTGTCTGCCGCGCTGCAGGCCATCACCTGCGTCTTCTACCTGTTCAACTGGTACGGGGAGAAGAAGAACCGCGTGCTGCGCTCCGAGGACGCGCTGTTTATTCTGGAGGAGACCCGCAAGGAGCGTGAGCGCCTGGGTCTCGAGGGGGTGTACGACG
Mutant B recombinant type 2 CGGCCCGCTCGCGGATGCCGGGAAGCCGCAGATCAGCATCTTCGTGTCTGCCGCGCTGCAGGCCATCACCTGCGTCTTCTACCTGTTCAACTGGTACGGGGAGAAGAAGAACCGCGTGCTGCGCTCCGAGGACGCGCTGTTTATTCTGGAGGAGACCCGCAAGGAGCGTGAGCGCCTGGGTCTCGAGGGGGTGTACGACG
Mutant G mutation           CGGCCCGCTCGCGGATGCCGGGAAGCCGCAGATCAGCATCTTCGTGTCTGCCGCGCTGCAGGCCATCACCTGCGTCTTCTACCTGTTCAACTGGTACGGGGAGAAGAAGAACCGCGTGCTGCGCTCCGAGGACGCGCTGTTTATTCTGGAGGAGACCCGCAAGGAGCGTGAGCGCCTGGGTCTCGAGGGGGTGTACGACG
Mutant G recombinant        CGGCCCGCTCGCGGATGCCGGGAAGCCGCAGATCAGCATCTTCGTGTCTGCCGCGCTGCAGGCCATCACCTGCGTCTTCTACCTGTTCAACTGGTACGGGGAGAAGAAGAACCGCGTGCTGCGCTCCGAGGACGCGCTGTTTATTCTGGAGGAGACCCGCAAGGAGCGTGAGCGCCTGGGTCTCGAGGGGGTGTACGACG
Mutant E mutation           CGGCCCGCTCGCGGATGCCGGGAAGCCGCAGATCAGCATCTTCGTGTCTGCCGCGCTGCAGGCCATCACCTGCGTCTTCTACCTGTTCAACTGGTACGGGGAGAAGAAGAACCGCGTGCTGCGCTCCGAGGACGCGCTGTTTATTCTGGAGGAGACCCGCAAGGAGCGTGAGCGCCTGGGTCTCGAGGGGGTGTACGACG
Mutant E gene conversion    CGGCCCGCTCGCGGATGCCGGGAAGCCGCAGATCAGCATCTTCGTGTCTGCCGCGCTGCAGGCCATCACCTGCGTCTTCTACCTGTTCAACTGGTACGGGGAGAAGAAGAACCGCGTGCTGCGCTCCGAGGACGCGCTGTTTATTCTGGAGGAGACCCGCAAGGAGCGTGAGCGCCTGGGTCTCGAGGGGGTGTACGACG

                            971     980       990       1000      1010      1020      1030      1040      1050      1060      1070      1080      1090      1100      1110      1120      1130      1140      1150      1160      1170
WT                          GCACGGCGGGTGCGCAGCATGGTGGTGCGGCGAAGGGGAAGAAGAGCCCGCAGCACTCGCACTCGGATGAGGACGTGGAGGGTGCCGTACGGGACGCCCTCAACGATGGTCAGCGCGACAACGGTGAACTTGTGCAGGACGTCTACGACGACGCGTATGACGACGGCGAGGGGGTGGCCGAGGGCGATGTGTACTACGGC
Mutant B recombinant type 1 GCACGGCGGGTGCGCAGCATGGTGGTGCGGCGAAGGGGAAGAAGAGCCCGCAGCACTCGCACTCGGATGAGGACGTGGAGGGTGCCGTACGGGACGCCCTCAACGATGGTCAGCGCGACAACGGTGAACTTGTGCAGGACGTCTACGACGACGCGTATGACGACGGCGAGGGGGTGGCCGAGGGCGATGTGTACTACGGC
Mutant B recombinant type 2 GCACGGCGGGTGCGCAGCATGGTGGTGCGGCGAAGGGGAAGAAGAGCCCGCAGCACTCGCACTCGGATGAGGACGTGGAGGGTGCCGTACGGGACGCCCTCAACGATGGTCAGCGCGACAACGGTGAACTTGTGCAGGACGTCTACGACGACGCGTATGACGACGGCGAGGGGGTGGCCGAGGGCGATGTGTACTACGGC
Mutant G mutation           GCACGGCGGGTGCGCAGCATGGTGGTGCGGCGAAGGGGAAGAAGAGCCCGCAGCACTCGCACTCGGATGAGGACGTGGAGGGTGCCGTACGGGACGCCCTCAACGATGGTCAGCGCGACAACGGTGAACTTGTGCAGGACGTCTACGACGACGCGTATGACGACGGCGAGGGGGTGGCCGAGGGCGATGTGTACTACGGC
Mutant G recombinant        GCACGGCGGGTGCGCAGCATGGTGGTGCGGCGAAGGGGAAGAAGAGCCCGCAGCACTCGCACTCGGATGAGGACGTGGAGGGTGCCGTACGGGACGCCCTCAACGATGGTCAGCGCGACAACGGTGAACTTGTGCAGGACGTCTACGACGACGCGTATGACGACGGCGAGGGGGTGGCCGAGGGCGATGTGTACTACGGC
Mutant E mutation           GCACGGCGGGTGCGCAGCATGGTGGTGCGGCGAAGGGGAAGAAGAGCCCGCAGCACTCGCACTCGGATGAGGACGTGGAGGGTGCCGTACGGGACGCCCTCAACGATGGTCAGCGCGACAACGGTGAACTTGTGCAGGACGTCTACGACGACGCGTATGACGACGGCGAGGGGGTGGCCGAGGGCGATGTGTACTACGGC
Mutant E gene conversion    GCACGGCGGGTGCGCAGCATGGTGGTGCGGCGAAGGGGAAGAAGAGCCCGCAGCACTCGCACTCGGATGAGGACGTGGAGGGTGCCGTACGGGACGCCCTCAACGATGGTCAGCGCGACAACGGTGAACTTGTGCAGGACGTCTACGACGACGCGTATGACGACGGCGAGGGGGTGGCCGAGGGCGATGTGTACTACGGC
```

```
                          1171     1180      1190      1200      1210      1220      1230      1240      1250      1260      1270      1280      1290      1300      1310      1320      1330      1340      1350      1360      1370
                          |--------+---------+---------+---------+---------+---------+---------+---------+---------+---------+---------+---------+---------+---------+---------+---------+---------+---------+---------+---------|
WT                        AAGCCGCCGGTGCCGTGCCTGTTCGGGCTGTTCGAGGCAAACACGGAGGTGATTTCGAAGAACTGGAAGATCTTCGTGTACAGCGTCGTCATGACCTGTGCTGTGATCACGATGCTGTGTGCCAACATGCTGGCCGACACGCTGGGCCTCCTGGTTGCGTGCGTCGTTGTGTCGACCATCTGCTGTACCACGTCCTTCTG
Mutant B recombinant type 1  AAGCCGCCGGTGCCGTGCCTGTTCGGGCTGTTCGAGGCAAACACGGAGGTGATTTCGAAGAACTGGAAGATCTTCGTGTACAGCGTCGTCATGACCTGTGCTGTGATCACGATGCTGTGTGCCAACATGCTGGCCGACACGCTGGGCCTCCTGGTTGCGTGCGTCGTTGTGTCGACCATCTGCTGTACCACGTCCTTCTG
Mutant B recombinant type 2  AAGCCGCCGGTGCCGTGCCTGTTCGGGCTGTTCGAGGCAAACACGGAGGTGATTTCGAAGAACTGGAAGATCTTCGTGTACAGCGTCGTCATGACCTGTGCTGTGATCACGATGCTGTGTGCCAACATGCTGGCCGACACGCTGGGCCTCCTGGTTGCGTGCGTCGTTGTGTCGACCATCTGCTGTACCACGTCCTTCTG
Mutant G mutation         AAGCCGCCGGTGCCGTGCCTGTTCGGGCTGTTCGAGGCAAACACGGAGGTGATTTCGAAGAACTGGAAGATCTTCGTGTACAGCGTCGTCATGACCTGTGCTGTGATCACGATGCTGTGTGCCAACATGCTGGCCGACACGCTGGGCCTCCTGGTTGCGTGCGTCGTTGTGTCGACCATCTGCTGTACCACGTCCTTCTG
Mutant G recombinant      AAGCCGCCGGTGCCGTGCCTGTTCGGGCTGTTCGAGGCAAACACGGAGGTGATTTCGAAGAACTGGAAGATCTTCGTGTACAGCGTCGTCATGACCTGTGCTGTGATCACGATGCTGTGTGCCAACATGCTGGCCGACACGCTGGGCCTCCTGGTTGCGTGCGTCGTTGTGTCGACCATCTGCTGTACCACGTCCTTCTG
Mutant E mutation         AAGCCGCCGGTGCCGTGCCTGTTCGGGCTGTTCGAGGCAAACACGGAGGTGATTTCGAAGAACTGGAAGATCTTCGTGTACAGCGTCGTCATGACCTGTGCTGTGATCACGATGCTGTGTGCCAACATGCTGGCCGACACGCTGGGCCTCCTGGTTGCGTGCGTCGTTGTGTCGACCATCTGCTGTACCACGTCCTTCTG
Mutant E gene conversion  AAGCCGCCGGTGCCGTGCCTGTTCGGGCTGTTCGAGGCAAACACGGAGGTGATTTCGAAGAACTGGAAGATCTTCGTGTACAGCGTCGTCATGACCTGTGCTGTGATCACGATGCTGTGTGCCAACATGCTGGCCGACACGCTGGGCCTCCTGGTTGCGTGCGTCGTTGTGTCGACCATCTGCTGTACCACGTCCTTCTG

                          1371     1380      1390      1400      1410      1420      1430      1440      1450      1460      1470      1480      1490      1500      1510      1520      1530      1540      1550      1560      1570
                          |--------+---------+---------+---------+---------+---------+---------+---------+---------+---------+---------+---------+---------+---------+---------+---------+---------+---------+---------+---------|
WT                        GGCCCTGCCGCTGGTGATTGCGAAGGCCAACGTGTTTGCATACCTGGATAAGGCTGTTTCCATCCGTGTGGGCGGTCCCCTAAATGCGTTCTACTTGAACACCTACCAGTGCCCTGGCAACCTGCCGAACTTCACCTACACCTTCTACAACACGGTGGCGGGCGTGATTAGCAGTGCTGTTGGTGTGATTACCGTGACGC
Mutant B recombinant type 1  GGCCCTGCCGCTGGTGATTGCGAAGGCCAACGTGTTTGCATACCTGGATAAGGCTGTTTCCATCCGTGTGGGCGGTCCCCTAAATGCGTTCTACTTGAACACCTACCAGTGCCCTGGCAACCTGCCGAACTTCACCTACACCTTCTACAACACGGTGGCGGGCGTGATTAGCAGTGCTGTTGGTGTGATTACCGTGACGC
Mutant B recombinant type 2  GGCCCTGCCGCTGGTGATTGCGAAGGCCAACGTGTTTGCATACCTGGATAAGGCTGTTTCCATCCGTGTGGGCGGTCCCCTAAATGCGTTCTACTTGAACACCTACCAGTGCCCTGGCAACCTGCCGAACTTCACCTACACCTTCTACAACACGGTGGCGGGCGTGATTAGCAGTGCTGTTGGTGTGATTACCGTGACGC
Mutant G mutation         GGCCCTGCCGCTGGTGATTGCGAAGGCCAACGTGTTTGCATACCTGGATAAGGCTGTTTCCATCCGTGTGGGCGGTCCCCTAAATGCGTTCTACTTGAACACCTACCAGTGCCCTGGCAACCTGCCGAACTTCACCTACACCTTCTACAACACGGTGGCGGGCGTGATTAGCAGTGCTGTTGGTGTGATTACCGTGACGC
Mutant G recombinant      GGCCCTGCCGCTGGTGATTGCGAAGGCCAACGTGTTTGCATACCTGGATAAGGCTGTTTCCATCCGTGTGGGCGGTCCCCTAAATGCGTTCTACTTGAACACCTACCAGTGCCCTGGCAACCTGCCGAACTTCACCTACACCTTCTACAACACGGTGGCGGGCGTGATTAGCAGTGCTGTTGGTGTGATTACCGTGACGC
Mutant E mutation         GGCCCTGCCGCTGGTGATTGCGAAGGCCAACGTGTTTGCATACCTGGATAAGGCTGTTTCCATCCGTGTGGGCGGTCCCCTAAATGCGTTCTACTTGAACACCTACCAGTGCCCTGGCAACCTGCCGAACTTCACCTACACCTTCTACAACACGGTGGCGGGCGTGATTAGCAGTGCTGTTGGTGTGATTACCGTGACGC
Mutant E gene conversion  GGCCCTGCCGCTGGTGATTGCGAAGGCCAACGTGTTTGCATACCTGGATAAGGCTGTTTCCATCCGTGTGGGCGGTCCCCTAAATGCGTTCTACTTGAACACCTACCAGTGCCCTGGCAACCTGCCGAACTTCACCTACACCTTCTACAACACGGTGGCGGGCGTGATTAGCAGTGCTGTTGGTGTGATTACCGTGACGC

                          1571     1580      1590      1600      1610      1620      1630      1640      1650      1660      1670      1680      1690      1700      1710      1720      1730      1740      1750      1760      1770
                          |--------+---------+---------+---------+---------+---------+---------+---------+---------+---------+---------+---------+---------+---------+---------+---------+---------+---------+---------+---------|
WT                        TGTTCAACTTCCTGTTCGCGAAGCATGGCTACTGCCTCACCTTCATTGTGACGACAATCATGCAGGTTATGGGTGGTGTGTTCGACATCATCATTGTGAAGCGGTGGAACCTGTACATTGGCATCCCTGACCACGCCATGTACATCTGGGGTGATGCTGTTGTGGGTGAGCTCGTGTACATGCTTGGCTTTATGCCGCAG
Mutant B recombinant type 1  TGTTCAACTTCCTGTTCGCGAAGCATGGCTACTGCCTCACCTTCATTGTGACGACAATCATGCAGGTTATGGGTGGTGTGTTCGACATCATCATTGTGAAGCGGTGGAACCTGTACATTGGCATCCCTGACCACGCCATGTACATCTGGGGTGATGCTGTTGTGGGTGAGCTCGTGTACATGCTTGGCTTTATGCCGCAG
Mutant B recombinant type 2  TGTTCAACTTCCTGTTCGCGAAGCATGGCTACTGCCTCACCTTCATTGTGACGACAATCATGCAGGTTATGGGTGGTGTGTTCGACATCATCATTGTGAAGCGGTGGAACCTGTACATTGGCATCCCTGACCACGCCATGTACATCTGGGGTGATGCTGTTGTGGGTGAGCTCGTGTACATGCTTGGCTTTATGCCGCAG
Mutant G mutation         TGTTCAACTTCCTGTTCGCGAAGCATGGCTACTGCCTCACCTTCATTGTGACGACAATCATGCAGGTTATGGGTGGTGTGTTCGACATCATCATTGTGAAGCGGTGGAACCTGTACATTGGCATCCCTGACCACGCCATGTACATCTGGGGTGATGCTGTTGTGGGTGAGCTCGTGTACATGCTTGGCTTTATGCCGCAG
Mutant G recombinant      TGTTCAACTTCCTGTTCGCGAAGCATGGCTACTGCCTCACCTTCATTGTGACGACAATCATGCAGGTTATGGGTGGTGTGTTCGACATCATCATTGTGAAGCGGTGGAACCTGTACATTGGCATCCCTGACCACGCCATGTACATCTGGGGTGATGCTGTTGTGGGTGAGCTCGTGTACATGCTTGGCTTTATGCCGCAG
Mutant E mutation         TGTTCAACTTCCTGTTCGCGAAGCATGGCTACTGCCTCACCTTCATTGTGACGACAATCATGCAGGTTATGGGTGGTGTGTTCGACATCATCATTGTGAAGCGGTGGAACCTGTACATTGGCATCCCTGACCACGCCATGTACATCTGGGGTGATGCTGTTGTGGGTGAGCTCGTGTACATGCTTGGCTTTATGCCGCAG
Mutant E gene conversion  TGTTCAACTTCCTGTTCGCGAAGCATGGCTACTGCCTCACCTTCATTGTGACGACAATCATGCAGGTTATGGGTGGTGTGTTCGACATCATCATTGTGAAGCGGTGGAACCTGTACATTGGCATCCCTGACCACGCCATGTACATCTGGGGTGATGCTGTTGTGGGTGAGCTCGTGTACATGCTTGGCTTTATGCCGCAG

                          1771     1780      1790      1800      1810      1820      1830      1840      1850      1860      1870      1880      1890      1900      1910      1920      1930      1940      1950      1960      1970
                          |--------+---------+---------+---------+---------+---------+---------+---------+---------+---------+---------+---------+---------+---------+---------+---------+---------+---------+---------+---------|
WT                        ATCGTGCTGCTGTCTCGCCTGTGCCCTCGTGGCTCGGAGAGTGTCGTGTATGCGCTGATGGCGGGCTTCGCGAGTCTTGGCCAGACCACCGCGTCGTCCCTCGGTGCGATCATCATGGAGTACGGCCTGCCTGTGTTCAAGACCCAGGATGACGGGTCTCGCTGCAACTACGACAACCTGCCGCTGCTGCTGTTCCTGTG
Mutant B recombinant type 1  ATCGTGCTGCTGTCTCGCCTGTGCCCTCGTGGCTCGGAGAGTGTCGTGTATGCGCTGATGGCGGGCTTCGCGAGTCTTGGCCAGACCACCGCGTCGTCCCTCGGTGCGATCATCATGGAGTACGGCCTGCCTGTGTTCAAGACCCAGGATGACGGGTCTCGCTGCAACTACGACAACCTGCCGCTGCTGCTGTTCCTGTG
Mutant B recombinant type 2  ATCGTGCTGCTGTCTCGCCTGTGCCCTCGTGGCTCGGAGAGTGTCGTGTATGCGCTGATGGCGGGCTTCGCGAGTCTTGGCCAGACCACCGCGTCGTCCCTCGGTGCGATCATCATGGAGTACGGCCTGCCTGTGTTCAAGACCCAGGATGACGGGTCTCGCTGCAACTACGACAACCTGCCGCTGCTGCTGTTCCTGTG
Mutant G mutation         ATCGTGCTGCTGTCTCGCCTGTGCCCTCGTGGCTCGGAGAGTGTCGTGTATGCGCTGATGGCGGGCTTCGCGAGTCTTGGCCAGACCACCGCGTCGTCCCTCGGTGCGATCATCATGGAGTACGGCCTGCCTGTGTTCAAGACCCAGGATGACGGGTCTCGCTGCAACTACGACAACCTGCCGCTGCTGCTGTTCCTGTG
Mutant G recombinant      ATCGTGCTGCTGTCTCGCCTGTGCCCTCGTGGCTCGGAGAGTGTCGTGTATGCGCTGATGGCGGGCTTCGCGAGTCTTGGCCAGACCACCGCGTCGTCCCTCGGTGCGATCATCATGGAGTACGGCCTGCCTGTGTTCAAGACCCAGGATGACGGGTCTCGCTGCAACTACGACAACCTGCCGCTGCTGCTGTTCCTGTG
Mutant E mutation         ATCGTGCTGCTGTCTCGCCTGTGCCCTCGTGGCTCGGAGAGTGTCGTGTATGCGCTGATGGCGGGCTTCGCGAGTCTTGGCCAGACCACCGCGTCGTCCCTCGGTGCGATCATCATGGAGTACGGCCTGCCTGTGTTCAAGACCCAGGATGACGGGTCTCGCTGCAACTACGACAACCTGCCGCTGCTGCTGTTCCTGTG
Mutant E gene conversion  ATCGTGCTGCTGTCTCGCCTGTGCCCTCGTGGCTCGGAGAGTGTCGTGTATGCGCTGATGGCGGGCTTCGCGAGTCTTGGCCAGACCACCGCGTCGTCCCTCGGTGCGATCATCATGGAGTACGGCCTGCCTGTGTTCAAGACCCAGGATGACGGGTCTCGCTGCAACTACGACAACCTGCCGCTGCTGCTGTTCCTGTG

                          1971     1980      1990      2000      2010      2020      2030      2040      2050      2060      2070      2080      2090      2100      2110      2120      2130      2140      2150      2160      2170
                          |--------+---------+---------+---------+---------+---------+---------+---------+---------+---------+---------+---------+---------+---------+---------+---------+---------+---------+---------+---------|
WT                        CAGCATGTGCACGCCGCTGCTGGTGATTCCGCTGAGCATGATACTGCTTCCGAAGGCGCGCATCTGCGACGATATCGACGTTGACGGCAAGGTGGTGCGCCAGGCCGTGGATAAGCAGGTCGCAGCTGCTCCGCTGTCGAGCTCCGACTCGGACGCGGTGATGGCTGCCGAGCCGCTTCATGGGAACAAGGCGGATGAGC
Mutant B recombinant type 1  CAGCATGTGCACGCCGCTGCTGGTGATTCCGCTGAGCATGATACTGCTTCCGAAGGCGCGCATCTGCGACGATATCGACGTTGACGGCAAGGTGGTGCGCCAGGCCGTGGATAAGCAGGTCGCAGCTGCTCCGCTGTCGAGCTCCGACTCGGACGCGGTGATGGCTGCCGAGCCGCTTCATGGGAACAAGGCGGATGAGC
Mutant B recombinant type 2  CAGCATGTGCACGCCGCTGCTGGTGATTCCGCTGAGCATGATACTGCTTCCGAAGGCGCGCATCTGCGACGATATCGACGTTGACGGCAAGGTGGTGCGCCAGGCCGTGGATAAGCAGGTCGCAGCTGCTCCGCTGTCGAGCTCCGACTCGGACGCGGTGATGGCTGCCGAGCCGCTTCATGGGAACAAGGCGGATGAGC
Mutant G mutation         CAGCATGTGCACGCCGCTGCTGGTGATTCCGCTGAGCATGATACTGCTTCCGAAGGCGCGCATCTGCGACGATATCGACGTTGACGGCAAGGTGGTGCGCCAGGCCGTGGATAAGCAGGTCGCAGCTGCTCCGCTGTCGAGCTCCGACTCGGACGCGGTGATGGCTGCCGAGCCGCTTCATGGGAACAAGGCGGATGAGC
Mutant G recombinant      CAGCATGTGCACGCCGCTGCTGGTGATTCCGCTGAGCATGATACTGCTTCCGAAGGCGCGCATCTGCGACGATATCGACGTTGACGGCAAGGTGGTGCGCCAGGCCGTGGATAAGCAGGTCGCAGCTGCTCCGCTGTCGAGCTCCGACTCGGACGCGGTGATGGCTGCCGAGCCGCTTCATGGGAACAAGGCGGATGAGC
Mutant E mutation         CAGCATGTGCACGCCGCTGCTGGTGATTCCGCTGAGCATGATACTGCTTCCGAAGGCGCGCATCTGCGACGATATCGACGTTGACGGCAAGGTGGTGCGCCAGGCCGTGGATAAGCAGGTCGCAGCTGCTCCGCTGTCGAGCTCCGACTCGGACGCGGTGATGGCTGCCGAGCCGCTTCATGGGAACAAGGCGGATGAGC
Mutant E gene conversion  CAGCATGTGCACGCCGCTGCTGGTGATTCCGCTGAGCATGATACTGCTTCCGAAGGCGCGCATCTGCGACGATATCGACGTTGACGGCAAGGTGGTGCGCCAGGCCGTGGATAAGCAGGTCGCAGCTGCTCCGCTGTCGAGCTCCGACTCGGACGCGGTGATGGCTGCCGAGCCGCTTCATGGGAACAAGGCGGATGAGC

                          2171     2180      2190     2199
                          |--------+---------+--------|
WT                        GCGAGGCAGCGCGCGGGGAGGCGGTGTAG
Mutant B recombinant type 1  GCGAGGCAGCGCGCGGGGAGGCGGTGTAG
Mutant B recombinant type 2  GCGAGGCAGCGCGCGGGGAGGCGGTGTAG
Mutant G mutation         GCGAGGCAGCGCGCGGGGAGGCGGTGTAG
Mutant G recombinant      GCGAGGCAGCGCGCGGGGAGGCGGTGTAG
Mutant E mutation         GCGAGGCAGCGCGCGGGGAGGCGGTGTAG
Mutant E gene conversion  GCGAGGCAGCGCGCGGGGAGGCGGTGTAG
```
