# Supplementary material for: Delving in folate metabolism in the parasite Leishmania major through a chemogenomic screen and methotrexate selection
Source: PLoS Negl Trop Dis. 2023 Jun 29;17(6):e0011458. doi: 10.1371/journal.pntd.0011458 (PMC10337921; doi:10.1371/journal.pntd.0011458)
Supplement: S1 Table — (DOCX) [file pntd.0011458.s012.docx]

**S1 Table. List of PCR and sequencing primers used.**

| **Gene of interest** | **Sequences** | **Uses ^a^** |
| --- | --- | --- |
| LmjF.06.0860 (DHFR-TS) | CGTCTAGATGTCCAGGGCAGCTGCGAGGTTTAAG | Forward primer for PCR amplification (XbaI restriction site)  Sanger sequencing |
| LmjF.06.0860 | CGAAGCTCTCTATACGGCCATCTCCATCTTGATCGCC | Reverse primer for PCR amplification  Sanger sequencing |
| LmjF.06.0860 | CGCGACGGCGCCTGCGTGTA | Sanger sequencing |
| LmjF.06.0860 | CGGCGCCAGAAGCTCCTCGA | gRNA containing T107I mutation (LmjF060860_342_revcom) |
| LmjF.06.0860 | CGTCGTGATGGGCCGCAAGACTTG | Forward primer for repair cassette containing T107I version |
| LmjF.06.0860 | CCAGACCGCCGTTCACCACCAC | Reverse primer for repair cassette containing T107I version |
| LmjF.06.0860 | CTTCTGGCGCGGCGTGTGCG | gRNA containing E291K mutation (LmjF060860_852) |
| LmjF.06.0860 | CACCATCAGCCTCTTCGGCGCCCAGATG | Forward primer for repair cassette containing E291K version |
| LmjF.06.0860 | GTCGAGAAACTCGCGCGAACCGTTGC | Reverse primer for repair cassette containing E291K version |
| LmjF.06.0860 | CACCATCAGCCTCTTCGGCGCCCAGATG | Sanger sequencing |
| LmjF.06.0860 | CCGCTGCTGACGACGAAGCGTGTCT | Sanger sequencing |
| LmjF.06.0860 | GCCGTAGACAGGGCCGAGGTCCATC | Sanger sequencing |
| LmjF.06.0860 | CGAGGAGCTTCTGGCGCCGCTG | Sanger sequencing |
| LmjF.06.0860 | CGGTTGTCGCGTAGGGAGAAGCGCATC | Sanger sequencing |
| LmjF.06.0860 | CAAGGAGGAGCGGCAGTACCTCGAG | Sanger sequencing  qPCR forward primer |
| LmjF.06.0860 | CTATACGGCCATCTCCATCTTGATCGCCG | Sanger sequencing |
| LmjF.06.0860 | CTATACGGCCATCTCCATCTTGATCGCCGG | qPCR reverse primer |
| LmjF.10.0380 (folate biopterin transporter) | CGTCTAGATGTCCCACAAGGAGGCCGCTCC | Forward primer for PCR amplification (XbaI restriction site)  Sanger sequencing |
| LmjF.10.0380 | CGAAGCTCTATTTCTGCGTCTGCCCGCGC | Reverse primer for PCR amplification  Sanger sequencing |
| LmjF.10.0380 | GCCGGGAAGCCGCAGATCAGCA | Sanger sequencing |
| LmjF.10.0385 (FT1) | CGTCTAGATGTCCTACAAGGAGGTCGCTCCCAAGC | Forward primer for PCR amplification (XbaI restriction site)  Sanger sequencing |
| LmjF.10.0385 | CGAAGCTCTCTATTTCTCTGAACCGCCTGCGGG | Reverse primer for PCR amplification  Sanger sequencing |
| LmjF.10.0385 | CCTGTCTTCGCTTTCGTCCA | gRNA containing G116R mutation (LmjF100385_324) |
| LmjF.10.0385 | CGCGGATCAGATTCTTACCGGTCAG | Forward primer for repair cassette containing G116R version |
| LmjF.10.0385 | CCGACGCAGGAGATGAACATGTACC | Reverse primer for repair cassette containing G116R version |
| LmjF.10.0385 | TGTGATGAACGGCCCGCTCGCG | Sanger sequencing |
| LmjF.10.0385 | CCATCAGCGCATACACGACACTCTC | Sanger sequencing  Reverse primer for repair cassette containing P555S version |
| LmjF.10.0385 | CAGATCGTGCTGCTGTCTCG | gRNA containing P555S mutation (LmjF100385_1685_revcom) |
| LmjF.10.0385 | GTGAAGCGGTGGAACCTGTACATTGG | Forward primer for repair cassette containing P555S version |
| LmjF.10.0385 | TTCACCGCGATGCTCTGCGA | gRNA containing G129D mutation (LmjF100385_364) |
| LmjF.10.0385 | GATTGATCGCTACGGCATCGACGTG | Forward primer for repair cassette containing G129D version |
| LmjF.10.0385 | GGCAGCAGGCCGTACAGGAG | Reverse primer for repair cassette containing G129D version |
| LmjF.10.0385 | CCTGCCGCTGGTGATTGCGA | gRNA containing A430V mutation (LmjF100385_1272) |
| LmjF.10.0385 | GCCAACATGCTGGCCGACACG | Forward primer for repair cassette containing A430V version |
| LmjF.10.0385 | GCCAGGGCACTGGTAGGTGTTCAAG | Reverse primer for repair cassette containing A430V version |
| LmjF.10.0385 | CAAGTCTTTTCAACACAAACCGCCTCCCTCTCCCTCTCGCAGGTCTCCATCGCCATCAGCATGATTGAACAAGATGGATTGC | Forward oligomer to produce cassette containing NEO targeting SKO |
| LmjF.10.0385 | ACTTCCCCTTAAACAACCGTCAAGCGCCACAACACTTTCTACAGAACCACGCAGCGCCAGTCAGAAGAACTCGTCAAGAAG | Reverse oligomer to produce cassette containing NEO targeting SKO |
| LmjF.10.0385 | GTGTTGTGGCGCTTGACGGTTGTTTAAGGG | Southern Blot 3’-UTR forward probe |
| LmjF.10.0385 | GAGTGGGTCAGGCGGGTGCACAAC | Southern Blot 3’-UTR reverse probe |
| LmjF.10.0390 (folate biopterin transporter) | CGTCTAGATGCTGGAGGCTCCGACGCATC | Forward primer for PCR amplification (XbaI restriction site)  Sanger sequencing |
| LmjF.10.0390 | CGAAGCTCTTCTGCTTCACGCGTTGCCCTACAC | Reverse primer for PCR amplification  Sanger sequencing |
| LmjF.10.0390 | GAGCCCGTAGCCCTCGACAGCGTC | Sanger sequencing |
| LmjF.10.0390 | CGGCTACACGAAGCGCTGGTACATGTTC | Sanger sequencing |
| LmjF.10.0390 | GCGAAGGGGAAGAAGAGCCCGCAG | Sanger sequencing |
| LmjF.10.0390 | GGCAACCTGCCGAACTTCACCTACACCTTC | Sanger sequencing |
| LmjF.10.0390 | GGCTCGGAGAGTGTCGTGTATGCGCTG | Sanger sequencing |
| LmjF.10.0400 (FT5) | CGTCTAGCATCAAGCACGCGTGCTGCAGCGATG | Forward primer for PCR amplification  Sanger sequencing |
| LmjF.10.0400 | CGAAGCTTCACGCGCCCTTCCTCTCTGCTTGC | Reverse primer for PCR amplification (HindIII restriction site)  Sanger sequencing |
| LmjF.10.0400 | CGCCGCCTTTGTGTTCCTGTGCTCC | Sanger sequencing |
| LmjF.10.0400 | GCTGGTGATTCCGCTGACTCTGCTG | Forward primer for repair cassette containing A687T version |
| LmjF.10.0400 | CCCTTCCTCTCTGCTTGCAGGTCTT | Reverse primer for repair cassette containing A687T version |
| LmjF.10.0400 | GCGCTGCTCTACGATGAGATGCGGA | Forward primer for repair cassette containing A316V version |
| LmjF.10.0400 | GCTGTAACGCCAGCACGCCCTCATC | Reverse primer for repair cassette containing A316V version |
| LmjF.10.0400 | CTAACTAACCACCGCAGGCATCACCGATCACCACCACCATCAAGCACGCGTGCTGCAGCGATGACCGAGTACAAGCCCACGGTGCG | Forward oligomer to produce cassette containing PURO targeting SKO |
| LmjF.10.0400 | CCTCCGGCCCGGCTCCTTCGCACACCCGCTCGCCGTTCGGAAACTGGCGTGCCCCAAGCCTCAGGCACCGGGCTTGCGG | Reverse oligomer to produce cassette containing PURO targeting SKO |
| LmjF.10.0400 | CCAGTGTAGGGCACATCGTGCGCCTC | Southern Blot 3’-UTR forward probe |
| LmjF.10.0400 | CTCACGATGCGAGATATGTGCGCCAACGG | Southern Blot 3’-UTR reverse probe |
| LmjF.17.0630 (hypothetical protein) | CGTCTAGATGGCCAGCGAAGACATCATTATCTTCCTCG | Forward primer for PCR amplification (XbaI restriction site)  Sanger sequencing |
| LmjF.17.0630 | CGAAGCTTTAAAGGAAACGCAGCACCTCAACCC | Reverse primer for PCR amplification (HindIII restriction site)  Sanger sequencing |
| LmjF.17.1130 (hypothetical protein) | CGTCTAGATGATGCGTCCCACGCGACGC | Forward primer for PCR amplification (XbaI restriction site)  Sanger sequencing |
| LmjF.17.1130 | CGAAGCTTCACAGCTCGAACACGTCCAACGACTGC | Reverse primer for PCR amplification (HindIII restriction site)  Sanger sequencing |
| LmjF.17.1130 | GTGTCGTCGCCAACGAGGCAC | Sanger sequencing |
| LmjF.17.1130 | TCGCGGCTTTGTGCGACTGCCTCC | Sanger sequencing |
| LmjF.17.1130 | GACAGCTGTGACTCAGCGAGCACAACGTC | Sanger sequencing |
| LmjF.17.1130 | GCCTCGGAGACGACGATGCCGATGC | Sanger sequencing |
| LmjF.17.1130 | GCTTCTCTCGCCGCACGCCAG | Sanger sequencing |
| LmjF.17.1130 | CAGACTGAGGAGGACGGCGGTGG | Sanger sequencing |
| LmjF.17.1130 | GAGGAGCCGCGTTGTTTCGCTGCC | Sanger sequencing |
| LmjF.17.1130 | GCATCGCCAGCGGAGCACGC | Sanger sequencing |
| LmjF.17.1360 (ALO) | CGTCTAGATGTCTGCTCATTCTGCGGCCCGTC | Forward primer for PCR amplification (XbaI restriction site)  Sanger sequencing |
| LmjF.17.1360 | CGAAGCTTTACGGCGTGCACGCGGTG | Reverse primer for PCR amplification (HindIII restriction site)  Sanger sequencing |
| LmjF.17.1360 | GGTGCAGCCGCGGATTCAGTGGA | Sanger sequencing |
| LmjF.17.1360 | GCAGCAGGGGACAGCTGCACAGG | gRNA containing P259S mutation (LmjF171360_797_revcom) |
| LmjF.17.1360 | CATACGGACGGCTGCTACGAGTCCTAC | Forward primer for repair cassette containing P259S version |
| LmjF.17.1360 | GCACAAAGTCCGTCGCAATGTACTTGAGG | Reverse primer for repair cassette containing P259S version |
| LmjF.17.1360 | CACTCGTTGGCCCACTGCTTGAAGAG | Sanger sequencing |
| LmjF.17.1360 | CGCCCTGCCGTATCCGCCCATCTCACACCCGTTCTGTCACTCGTGCATTGTACCTTGACCAATGATTGAACAAGATGGATTGC | Forward oligomer to produce cassette containing PURO targeting SKO |
| LmjF.17.1360 | CAGGCAGCCTTTGCTGCACACAAACGTTCGCACGCACAAAATCTTAGGTACCAGGTCTGTTCAGAAGAACTCGTCAAGAAG | Reverse oligomer to produce cassette containing PURO targeting SKO |
| LmjF.17.1360 | GTGCGTGCGAACGTTTGTGTGCAG | Southern Blot 3’-UTR forward probe |
| LmjF.17.1360 | GCAAGCATGAGAGAGAGGGCATGC | Southern Blot 3’-UTR reverse probe |
| LmjF.19.0920 (folate biopterin transporter) | ATGGCGTCGAAGTCACACCTTGAGGCGG | Forward primer for PCR amplification  Sanger sequencing |
| LmjF.19.0920 | TCACTGCTTGCCGGCGGCACTG | Reverse primer for PCR amplification  Sanger sequencing |
| LmjF.19.0920 | CGCGCTGGTGAGCTGGATCTG | Sanger sequencing |
| LmjF.19.0920 | CTACGAAAACACACGCACACACCACAGACCAAGGCTTACCCTATCCAGTAAGAACGAACAATGATTGAACAAGATGGATTGC | Forward oligomer to produce cassette containing PURO targeting DKO |
| LmjF.19.0920 | CTTCTAATGTCCGCTTTCTGGTTCCAGAATTTCTCTTCGGAAGGATTGCCTTTTATGTGCTCAGAAGAACTCGTCAAGAAG | Reverse oligomer to produce cassette containing PURO targeting DKO |
| LmjF.19.0920 | GGCAATCCTTCCGAAGAGAAATTCTGG | Southern Blot 3’-UTR forward probe |
| LmjF.19.0920 | GAAGATGGGTGTCGACAGGTGTC | Southern Blot 3’-UTR reverse probe |
| LmjF.21.1860 (beta tubulin) | GGATCCCGAACAACATCAAG | qPCR forward primer |
| LmjF.21.1860 | AGGTGTTGTTGCCGATGAAG | qPCR reverse primer |
| LmjF.20.0120 (NADH-ubiquinone oxidoreductase complex I subunit) | CGTCTAGATGCGTCGTGTCGCCCGC | Forward primer for PCR amplification (XbaI restriction site)  Sanger sequencing |
| LmjF.20.0120 (Hypothetical protein) | CGAAGCTTTACCTCACCTTCTTCGCCTCGCC | Reverse primer for PCR amplification (HindIII restriction site)  Sanger sequencing |
| LmjF.22.1210 | CGTCTAGATGCATTACGGCATCCCCTCGGC | Forward primer for PCR amplification (XbaI restriction site)  Sanger sequencing |
| LmjF.22.1210 | CGATGCACTATGACTCCGTTTCGTCACCCTCAAGCG | Reverse primer for PCR amplification  Sanger sequencing |
| LmjF.22.1210 | GCAGCGCCGGTTGCTGGCCTC | Sanger sequencing |
| LmjF.23.0270 (PTR1) | CGTCTAGATGACTGCTCCGACCGTGCCG | Forward primer for PCR amplification (XbaI restriction site)  Sanger sequencing |
| LmjF.23.0270 | CGAAGCTTCAGGCCCGGGTAAGGCTGTAG | Reverse primer for PCR amplification (HindIII restriction site)  Sanger sequencing |
| LmjF.23.0270 | AGACAGCGTACCCCTCCGCG | gRNA containing A28T mutation (LmjF230270_100_revcom) |
| LmjF.23.0270 | ATGACTGCTCCGACCGTGCCGGTG | Forward primer for repair cassette containing A28T version |
| LmjF.23.0270 | GTGATGGCGCTGTTCGGTCGCCTTG | Reverse primer for repair cassette containing A28T version |
| LmjF.23.0270 | AACGTCGCTCACCTCTGCGG | gRNA containing S253F mutation (LmjF230270_780_revcom) |
| LmjF.23.0270 | GCTGCAGATTCGAGTGAACGGCGTTGGTC | Forward primer for repair cassette containing S253F version |
| LmjF.23.0270 | CAGGCCCGGGTAAGGCTGTAGCCAC | Reverse primer for repair cassette containing S253F version  qPCR reverse primer |
| LmjF.23.0270 | CGCCGCAGAGGTGAGCGACGTTGTTATC | qPCR forward primer |
| LmjF.23.0390 (hypothetical protein) | CGTCTAGGATGTCCCGCCAACCCAATGAAGACAC | Forward primer for PCR amplification  Sanger sequencing |
| LmjF.23.0390 | CGATGCACGTCGTTTCTCCTTTATCTCTCACGCCG | Reverse primer for PCR amplification  Sanger sequencing |
| LmjF.23.0390 | GCGGCTGTGGCGGCGGCTTT | Sanger sequencing |
| LmjF.23.0390 | CGCGGCGGCAAACGAGTCGACAG | Sanger sequencing |
| LmjF.27.1940 (protein kinase-like protein) | CGTCTAGATGCAGGTCAACTCGCTCTTGGAGCG | Forward primer for PCR amplification (XbaI restriction site)  Sanger sequencing |
| LmjF.27.1940 | CGAAGCTTCACTCATCTCGGTCTTGGTTTGCGAGG | Reverse primer for PCR amplification (HindIII restriction site)  Sanger sequencing |
| LmjF.35.4045 (hypothetical protein) | ATGACGACGCCGGCTCCATCG | Forward primer for PCR amplification  Sanger sequencing |
| LmjF.35.4045 | TCAAGAAGACGCAAAGGGAAAAGACGGTGC | Reverse primer for PCR amplification  Sanger sequencing |
| LmjF.35.4045 | GGAACACGCAACTCGTCCCCGTC | Forward primer for 5’-UTR amplification of SKO cassette |
| LmjF.35.4045 | GGGAAGGAGAAGAGAGAGCTCGGTACGTTGGTTTCCTAGCATTCGAGTAGCTC | Reverse primer for 5’-UTR amplification of SKO cassette |
| LmjF.35.4045 | TACCGAGCTCTCTCTTCTCCTTCCC | Forward primer for gene amplification of SKO cassette |
| LmjF.35.4045 | TCGCATCACTCTCCCCTCGCCCTTATCAGGCACCGGGCTTGCG | Forward primer for gene amplification of SKO cassette with puro |
| LmjF.35.4045 | TCGCATCACTCTCCCCTCGCCCTTATCAGAAGAACTCGTCAAGAAGGCGATAGAAG | Forward primer for gene amplification of SKO cassette with neo |
| LmjF.35.4045 | TAAGGGCGAGGGGAGAGTGATG | Forward primer for 3’-UTR amplification of SKO cassette |
| LmjF.35.4045 | TGCCAATGCAGCAAGGCAAGT | Reverse primer for 3’-UTR amplification of SKO cassette |
| LmjF.36.0510 (mitochondrial carrier protein) | CGTCTAGATGCTGTCGACAACCTCATCGTCCTTTTCGACG | Forward primer for PCR amplification (XbaI restriction site)  Sanger sequencing |
| LmjF.36.0510 | CGAAGCTCTAGGGGGTGTCACCGCG | Reverse primer for PCR amplification  Sanger sequencing |

**^a^** For PCR amplification of each gene, the pair of primers named “Forward primer for PCR amplification” and “Reverse primer for PCR amplification” were used. Integrity of sequences were verified using primers named “Sanger sequencing”. qPCR experiments were done with pair of primers named “qPCR forward primer” and “qPCR reverse primer”.
