# Supplementary material for: Delving in folate metabolism in the parasite Leishmania major through a chemogenomic screen and methotrexate selection
Source: PLoS Negl Trop Dis. 2023 Jun 29;17(6):e0011458. doi: 10.1371/journal.pntd.0011458 (PMC10337921; doi:10.1371/journal.pntd.0011458)
Supplement: S2 Table — (DOCX) [file pntd.0011458.s013.docx]

**S2 Table. Sequence Reads Archive BioSamples accessions for samples sequenced in this study.**

| **SRA accession** | **sample_name** |
| --- | --- |
| SAMN33578543 | LmajorWT |
| SAMN33578544 | LmajorMTX_A |
| SAMN33578545 | LmajorMTX_B |
| SAMN33578546 | LmajorMTX_C |
| SAMN33578547 | LmajorMTX_D |
| SAMN33578548 | LmajorMTX_E |
| SAMN33578549 | LmajorMTX_F |
| SAMN33578550 | LmajorMTX_G |
| SAMN33578551 | LmajorMTX_H |
| SAMN33578552 | LmajorMTX_I |
| SAMN33578553 | LmajorMTX_J |
| SAMN33578554 | LmajorMTX_L |
| SAMN33578555 | LmajorMTX_M |
| SAMN33578556 | LmajorMTX_N |
| SAMN33578557 | LmajorMTX_P |
| SAMN33578558 | LmajorMTX_Q |
| SAMN33578559 | LmajorMTX_R |
| SAMN33578560 | LmajorMTX_S |
| SAMN33578561 | LmajorMTX_T |
| SAMN33578562 | LmajorMTX_U |
| SAMN33578563 | LmajorMTX_V |
