# Supplementary material for: Delving in folate metabolism in the parasite Leishmania major through a chemogenomic screen and methotrexate selection
Source: PLoS Negl Trop Dis. 2023 Jun 29;17(6):e0011458. doi: 10.1371/journal.pntd.0011458 (PMC10337921; doi:10.1371/journal.pntd.0011458)
Supplement: S4 Table — (DOCX) [file pntd.0011458.s015.docx]

**S4 Table.** Sanger sequencing of CRISPR-edited cells resistant to MTX.

| **Gene ID** | **Function** | **Mutations** | **Sanger sequencing ^a^** | **Chromatogram ^b^** |
| --- | --- | --- | --- | --- |
| LmjF.06.0860 | DHFR-TS | DNA:  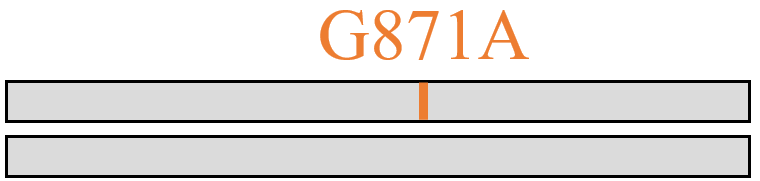  Protein:  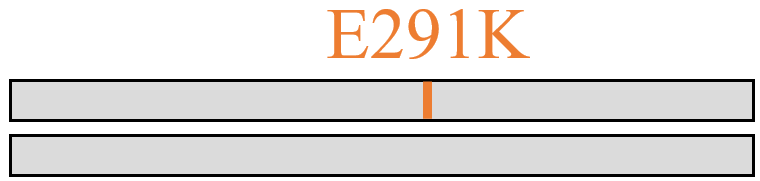 | ATGTCCAGGGCAGCTGCGAGGTTTAAGATTCCGATGCCGGAGACGAAGGCAGACTTTGCTTTCCCCTCCCTGCGCGCCTTCTCCATCGTCGTGGCCCTCGATATGCAGCACGGCATCGGCGACGGCGAGTCGATCCCGTGGCGGGTGCCGGAGGACATGACGTTCTTCAAGAACCAGACGACGCTGCTGCGCAACAAGAAGCCGCCGACGGAGAAGAAGCGCAACGCCGTCGTGATGGGCCGCAAGACTTGGGAGAGCGTCCCGGTAAAGTTCCGACCACTCAAGGGACGGCTGAACATCGTGTTATCCTCGAAGGCCACCGTCGAGGAGCTTCTGGCGCCGCTGCCGGAGGGACAGCGCGCGGCGGCGGCGCAGGATGTGGTGGTGGTGAACGGCGGTCTGGCCGAGGCGCTCCGCCTCCTCGCACGCCCGCTGTACTGCAGCTCCATCGAGACAGCGTATTGCGTCGGTGGTGCGCAGGTTTACGCGGACGCCATGCTGTCGCCGTGCATCGAGAAACTGCAGGAAGTGTACCTGACCCGCATCTACGCGACGGCGCCTGCGTGTACGCGCTTCTTTCCGTTTCCGCCCGAGAACGCGGCCACGGCGTGGGACCTGGCGTCGTCTCAGGGACGCCGCAAGAGCGAGGCGGAGGGCCTCGAGTTCGAGATCTGCAAGTACGTGCCGCGCAACCACGAGGAGCGGCAGTACCTTGAGCTGATTGACCGCATCATGAAGACGGGGATCGTGAAGGAGGACCGCACCGGCGTGGGCACCATCAGCCTCTTCGGCGCCCAGATGCGCTTCTCCCTACGCGACAACCGCCTGCCGCTGCTGACGACGAAGCGTGTCTTCTGGCGCGGCGTGTGC**R**AGGAGCTGCTGTGGTTCCTGCGCGGGGAGACGAGTGCGCAGCTGCTGGCAGACAAGGACATTCACATCTGGGACGGCAACGGTTCGCGCGAGTTTCTCGACAGCCGCGGCTTGACAGAGAATAAGGAGATGGACCTCGGCCCTGTCTACGGCTTCCAGTGGCGCCACTTCGGGGCAGATTACAAGGGGTTTGAAGCGAACTACGACGGCGAAGGGGTGGACCAGATCAAGCTCATCGTGGAGACCATCAAGACGAACCCGAACGACCGCCGCCTCCTAGTCACTGCCTGGAACCCGTGCGCGCTGCAAAAGATGGCGCTGCCGCCGTGCCACTTGCTTGCTCAGTTCTACGTGAACACAGACACGAGCGAGCTATCCTGCATGTTGTACCAGCGCTCGTGTGACATGGGTCTTGGCGTCCCCTTCAACATTGCCTCCTACGCGCTGCTCACCATCCTCATTGCCAAGGCGACGGGTCTGCGGCCTGGTGAGCTTGTGCACACCCTCGGCGACGCCCACGTCTACCGCAACCACGTTGATGCCCTCAAGGCGCAGCTCGAGCGAGTCCCGCACGCGTTCCCGACCCTCATCTTCAAGGAGGAGCGGCAGTACCTCGAGGACTACGAGTTGACGGACATGGAGGTGATCGACTACGTTCCACACCCGGCGATCAAGATGGAGATGGCCGTATAG | 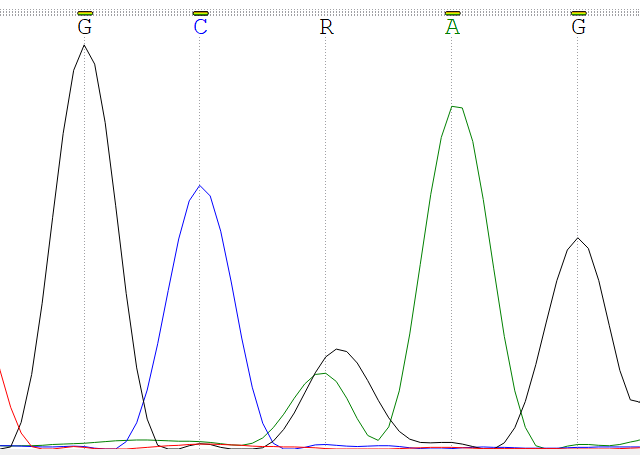 |
|  |  | DNA:  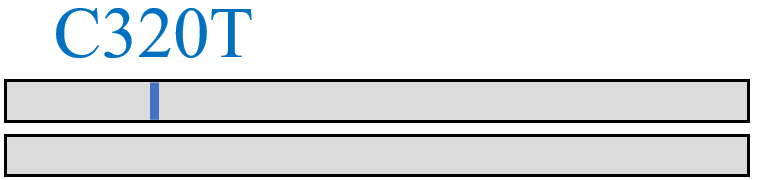  Protein:  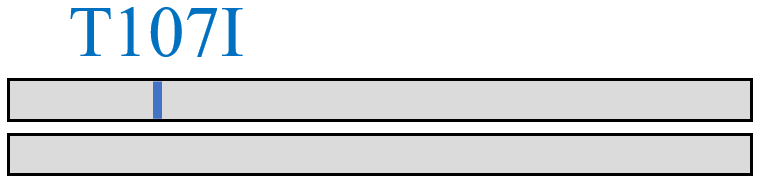 | ATGTCCAGGGCAGCTGCGAGGTTTAAGATTCCGATGCCGGAGACGAAGGCAGACTTTGCTTTCCCCTCCCTGCGCGCCTTCTCCATCGTCGTGGCCCTCGATATGCAGCACGGCATCGGCGACGGCGAGTCGATCCCGTGGCGGGTGCCGGAGGACATGACGTTCTTCAAGAACCAGACGACGCTGCTGCGCAACAAGAAGCCGCCGACGGAGAAGAAGCGCAACGCCGTCGTGATGGGCCGCAAGACTTGGGAGAGCGTCCCGGTAAAGTTCCGACCACTCAAGGGACGGCTGAACATCGTGTTATCCTCGAAGGCCA**Y**CGTCGAGGAGCTTCTGGCGCCGCTGCCGGAGGGACAGCGCGCGGCGGCGGCGCAGGATGTGGTGGTGGTGAACGGCGGTCTGGCCGAGGCGCTCCGCCTCCTCGCACGCCCGCTGTACTGCAGCTCCATCGAGACAGCGTATTGCGTCGGTGGTGCGCAGGTTTACGCGGACGCCATGCTGTCGCCGTGCATCGAGAAACTGCAGGAAGTGTACCTGACCCGCATCTACGCGACGGCGCCTGCGTGTACGCGCTTCTTTCCGTTTCCGCCCGAGAACGCGGCCACGGCGTGGGACCTGGCGTCGTCTCAGGGACGCCGCAAGAGCGAGGCGGAGGGCCTCGAGTTCGAGATCTGCAAGTACGTGCCGCGCAACCACGAGGAGCGGCAGTACCTTGAGCTGATTGACCGCATCATGAAGACGGGGATCGTGAAGGAGGACCGCACCGGCGTGGGCACCATCAGCCTCTTCGGCGCCCAGATGCGCTTCTCCCTACGCGACAACCGCCTGCCGCTGCTGACGACGAAGCGTGTCTTCTGGCGCGGCGTGTGCGAGGAGCTGCTGTGGTTCCTGCGCGGGGAGACGAGTGCGCAGCTGCTGGCAGACAAGGACATTCACATCTGGGACGGCAACGGTTCGCGCGAGTTTCTCGACAGCCGCGGCTTGACAGAGAATAAGGAGATGGACCTCGGCCCTGTCTACGGCTTCCAGTGGCGCCACTTCGGGGCAGATTACAAGGGGTTTGAAGCGAACTACGACGGCGAAGGGGTGGACCAGATCAAGCTCATCGTGGAGACCATCAAGACGAACCCGAACGACCGCCGCCTCCTAGTCACTGCCTGGAACCCGTGCGCGCTGCAAAAGATGGCGCTGCCGCCGTGCCACTTGCTTGCTCAGTTCTACGTGAACACAGACACGAGCGAGCTATCCTGCATGTTGTACCAGCGCTCGTGTGACATGGGTCTTGGCGTCCCCTTCAACATTGCCTCCTACGCGCTGCTCACCATCCTCATTGCCAAGGCGACGGGTCTGCGGCCTGGTGAGCTTGTGCACACCCTCGGCGACGCCCACGTCTACCGCAACCACGTTGATGCCCTCAAGGCGCAGCTCGAGCGAGTCCCGCACGCGTTCCCGACCCTCATCTTCAAGGAGGAGCGGCAGTACCTCGAGGACTACGAGTTGACGGACATGGAGGTGATCGACTACGTTCCACACCCGGCGATCAAGATGGAGATGGCCGTATAG | 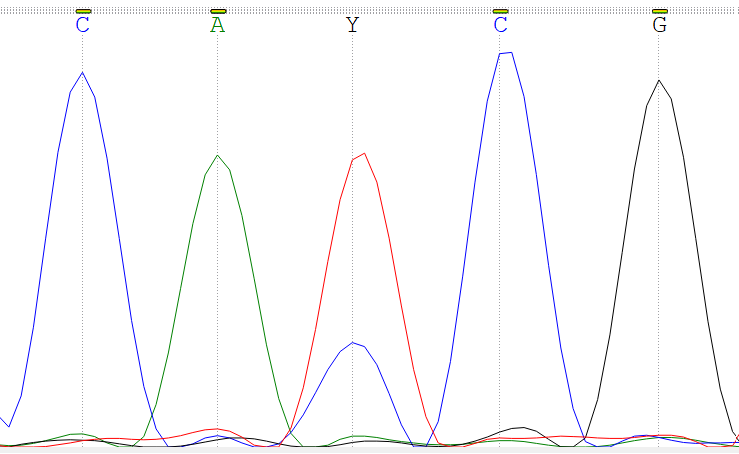 |
| LmjF.10.0385 | FT1 | DNA:  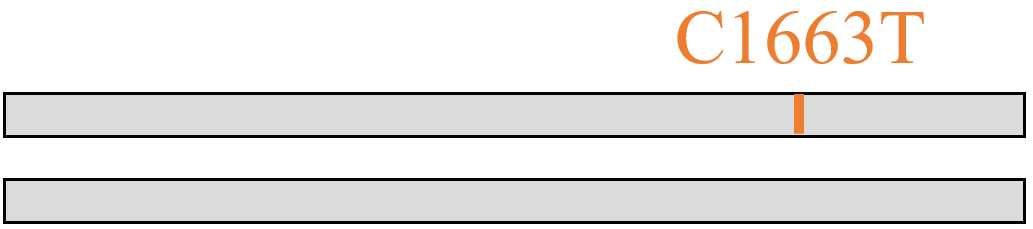  Protein:  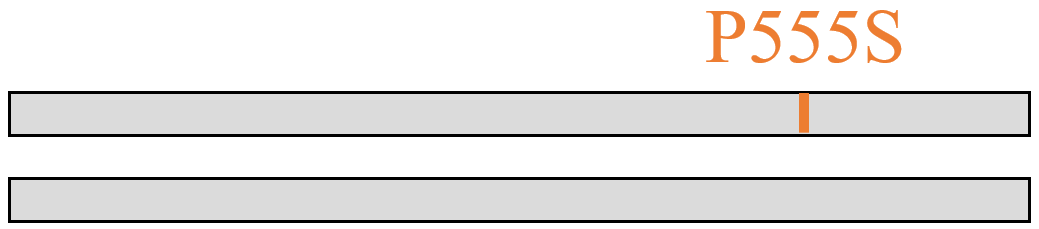 | ATGTCCTACAAGGAGGTCGCTCCCAAGCGCGAGAAGGATGCCGCCTCAGACGCCGCGGGTATAGCGGCGGTGCCTGAGGCGGCTGCCGCGGGAAATGATGACAAGTACATCCACCCCGAGGCAGCATCCCTGTTCGCCAGGTGCCCGTGTGCTCGCCGCGTCCCCGTGTTTGGTGACGCTGTCGAGGGCTACGGGCTCAAGTTCATCATCGCCCTAGGCGCTAGCAACCTGCTCTGCAAGGGTATCGCGGATCAGATTCTTACCGGTCAGACGTACGCCATGATGATTGATCGCTACGGCATCGACGTGGCCCGCTACCAGCGCCTGTCTTCGCTTTCGTCCATGGGGTGGTCCATCAAGGCCTTCACCGCGATGCTCTGCGACGGCTTCGCCTTCCTCGGCTACACGAAGCGCTGGTACATGTTCATCTCCTGCGTCGGCGGTGGTGTTTTCGCCCTCCTGTACGGCCTGCTGCCCGCGAAGGAGGCGTCGGCTGATGTGGCGTGTGCCTTCATCTTCCTGTCGTGCTGGGGCAAGGCCAACGTGGATATCCTGTCCCAGGGCCATTACAGTCGACTGATGCGCGAGAACCCGAAGCCTGGCCCGGCGCTGGTGAGCTGGATCTGGCTCTGGATCATGACCGGCTCGCTCATCGCGACTGTGATGAACGGCCCGCTCGCGGATGCCGGGAAGCCGCAGATCAGCATCTTCGTGTCTGCCGCGCTGCAGGCCATCACCTGCGTCTTCTACCTGTTCAACTGGTACGGGGAGAAGAAGAACCGCGTGCTGCGCTCCGAGGACGCGCTGTTTATTCTGGAGGAGACCCGCAAGGAGCGTGAGCGCCTGGGTCTCGAGGGGGTGTACGACGGCACGGCGGGTGCGCAGCATGGTGGTGCGGCGAAGGGGAAGAAGAGCCCGCAGCACTCGCACTCGGATGAGGACGTGGAGGGTGCCGTACGGGACGCCCTCAACGATGGTCAGCGCGACAACGGTGAACTTGTGCAGGACGTCTACGACGACGCGTATGACGACGGCGAGGGGGTGGCCGAGGGCGATGTGTACTACGGCAAGCCGCCGGTGCCGTGCCTGTTCGGGCTGTTCGAGGCAAACACGGAGGTGATTTCGAAGAACTGGAAGATCTTCGTGTACAGCGTCGTCATGACCTGTGCTGTGATCACGATGCTGTGTGCCAACATGCTGGCCGACACGCTGGGCCTCCTGGTTGCGTGCGTCGTTGTGTCGACCATCTGCTGTACCACGTCCTTCTGGGCCCTGCCGCTGGTGATTGCGAAGGCCAACGTGTTTGCATACCTGGATAAGGCTGTTTCCATCCGTGTGGGCGGTCCCCTAAATGCGTTCTACTTGAACACCTACCAGTGCCCTGGCAACCTGCCGAACTTCACCTACACCTTCTACAACACGGTGGCGGGCGTCATCAACACCATCGTTGGTATGATTACCGTGACGCTGTTCAACTTCCTGTTCGCGAAGCATGGCTACCGCCTCACCTTCATTGTGACGACAATCATGCAGGTTATGGGTGGTGTGTTCGACATCATCATTGTGAAGCGGTGGAACCTGTACATTGGCATCCCTGACCACGCCATGTACATCTGGGGTGATGCTGTTGTGGGTGAGCTCGTGTACATGCTTGGCTTTATG**Y**CGCAGATCGTGCTGCTGTCTCGCCTGTGCCCTCGTGGCTCGGAGAGTGTCGTGTATGCGCTGATGGCGGGCTTCGCCCGCCTCGGCCGAACGACCGCGTCGTCCCTCGGTGCCATCCTTCTGGAGTACGGCCTGCCTGTGTTCAAGACCCAGGATGACGGGTCTCGCTGCAACTACGACAACCTGCCGCTGCTGTTGTTCGTGACCAGCATGTGCACGCCGCTGCTGGCGATTCCGCTGAGCATGATACTGCTTCCGAAGGCGCGCATCTGCGACGATATCGACGTTGACGGCAAGGTGGTGCGCCAGGCCGTGGATAAGCAGGTCGCAGCTGCTCCGCTGTCGAGCTCCGACTCGGACGCGGTGATGGCTGCCGAGCCGCTTCATGGGAACAAGGCGGATGAGCGCGAGGCAGCGCGCGGGGAGACGGTGCAGGGTAAACCCGCAGGCGGTTCAGAGAAATAG | 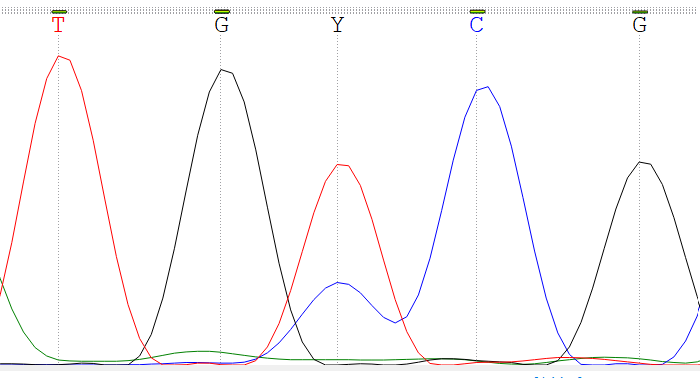 |
|  |  | DNA:  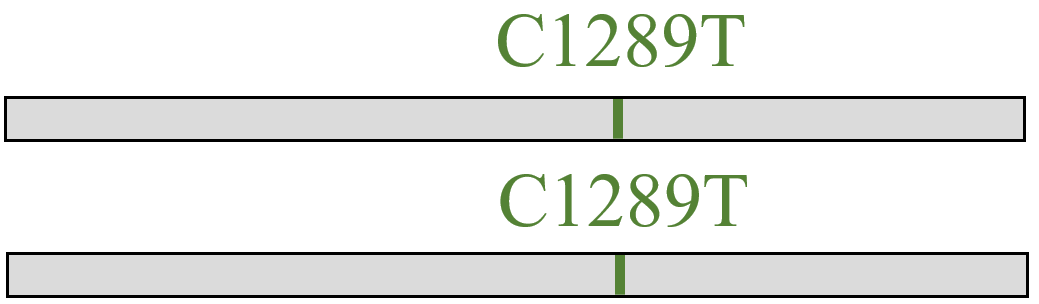  Protein:  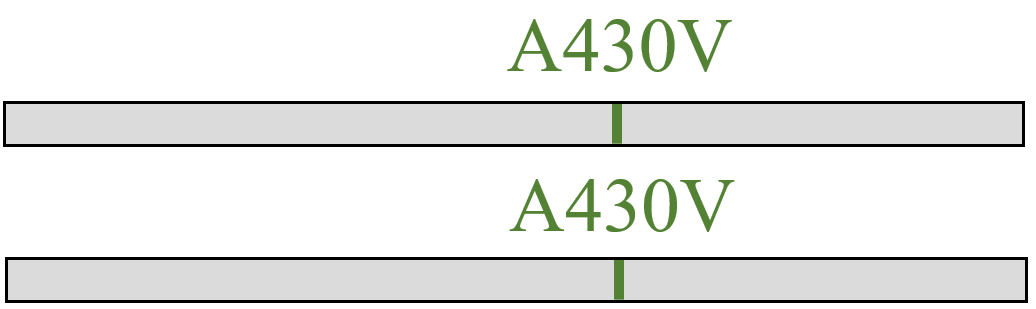 | ATGTCCTACAAGGAGGTCGCTCCCAAGCGCGAGAAGGATGCCGCCTCAGACGCCGCGGGTATAGCGGCGGTGCCTGAGGCGGCTGCCGCGGGAAATGATGACAAGTACATCCACCCCGAGGCAGCATCCCTGTTCGCCAGGTGCCCGTGTGCTCGCCGCGTCCCCGTGTTTGGTGACGCTGTCGAGGGCTACGGGCTCAAGTTCATCATCGCCCTAGGCGCTAGCAACCTGCTCTGCAAGGGTATCGCGGATCAGATTCTTACCGGTCAGACGTACGCCATGATGATTGATCGCTACGGCATCGACGTGGCCCGCTACCAGCGCCTGTCTTCGCTTTCGTCCATGGGGTGGTCCATCAAGGCCTTCACCGCGATGCTCTGCGACGGCTTCGCCTTCCTCGGCTACACGAAGCGCTGGTACATGTTCATCTCCTGCGTCGGCGGTGGTGTTTTCGCCCTCCTGTACGGCCTGCTGCCCGCGAAGGAGGCGTCGGCTGATGTGGCGTGTGCCTTCATCTTCCTGTCGTGCTGGGGCAAGGCCAACGTGGATATCCTGTCCCAGGGCCATTACAGTCGACTGATGCGCGAGAACCCGAAGCCTGGCCCGGCGCTGGTGAGCTGGATCTGGCTCTGGATCATGACCGGCTCGCTCATCGCGACTGTGATGAACGGCCCGCTCGCGGATGCCGGGAAGCCGCAGATCAGCATCTTCGTGTCTGCCGCGCTGCAGGCCATCACCTGCGTCTTCTACCTGTTCAACTGGTACGGGGAGAAGAAGAACCGCGTGCTGCGCTCCGAGGACGCGCTGTTTATTCTGGAGGAGACCCGCAAGGAGCGTGAGCGCCTGGGTCTCGAGGGGGTGTACGACGGCACGGCGGGTGCGCAGCATGGTGGTGCGGCGAAGGGGAAGAAGAGCCCGCAGCACTCGCACTCGGATGAGGACGTGGAGGGTGCCGTACGGGACGCCCTCAACGATGGTCAGCGCGACAACGGTGAACTTGTGCAGGACGTCTACGACGACGCGTATGACGACGGCGAGGGGGTGGCCGAGGGCGATGTGTACTACGGCAAGCCGCCGGTGCCGTGCCTGTTCGGGCTGTTCGAGGCAAACACGGAGGTGATTTCGAAGAACTGGAAGATCTTCGTGTACAGCGTCGTCATGACCTGTGCTGTGATCACGATGCTGTGTGCCAACATGCTGGCCGACACGCTGGGCCTCCTGGTTGCGTGCGTCGTTGTGTCGACCATCTGCTGTACCACGTCCTTCTGGGCCCTGCCGCTGGTGATTG**T**GAAGGCCAACGTGTTTGCATACCTGGATAAGGCTGTTTCCATCCGTGTGGGCGGTCCCCTAAATGCGTTCTACTTGAACACCTACCAGTGCCCTGGCAACCTGCCGAACTTCACCTACACCTTCTACAACACGGTGGCGGGCGTCATCAACACCATCGTTGGTATGATTACCGTGACGCTGTTCAACTTCCTGTTCGCGAAGCATGGCTACCGCCTCACCTTCATTGTGACGACAATCATGCAGGTTATGGGTGGTGTGTTCGACATCATCATTGTGAAGCGGTGGAACCTGTACATTGGCATCCCTGACCACGCCATGTACATCTGGGGTGATGCTGTTGTGGGTGAGCTCGTGTACATGCTTGGCTTTATGCCGCAGATCGTGCTGCTGTCTCGCCTGTGCCCTCGTGGCTCGGAGAGTGTCGTGTATGCGCTGATGGCGGGCTTCGCCCGCCTCGGCCGAACGACCGCGTCGTCCCTCGGTGCCATCCTTCTGGAGTACGGCCTGCCTGTGTTCAAGACCCAGGATGACGGGTCTCGCTGCAACTACGACAACCTGCCGCTGCTGTTGTTCGTGACCAGCATGTGCACGCCGCTGCTGGCGATTCCGCTGAGCATGATACTGCTTCCGAAGGCGCGCATCTGCGACGATATCGACGTTGACGGCAAGGTGGTGCGCCAGGCCGTGGATAAGCAGGTCGCAGCTGCTCCGCTGTCGAGCTCCGACTCGGACGCGGTGATGGCTGCCGAGCCGCTTCATGGGAACAAGGCGGATGAGCGCGAGGCAGCGCGCGGGGAGACGGTGCAGGGTAAACCCGCAGGCGGTTCAGAGAAATAG | 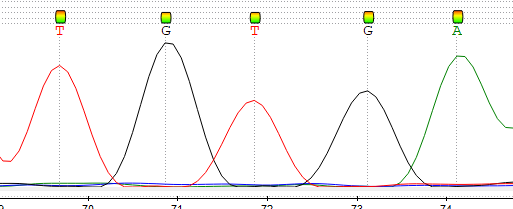 |
|  |  | DNA:  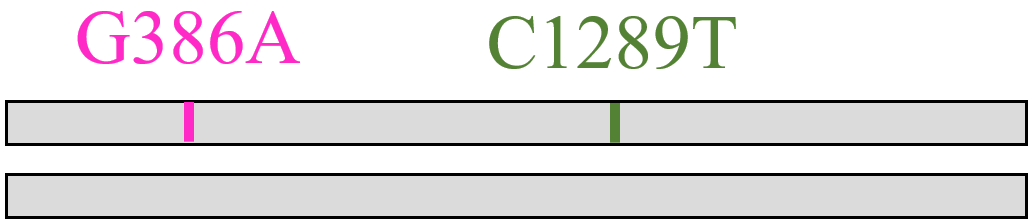  Protein:  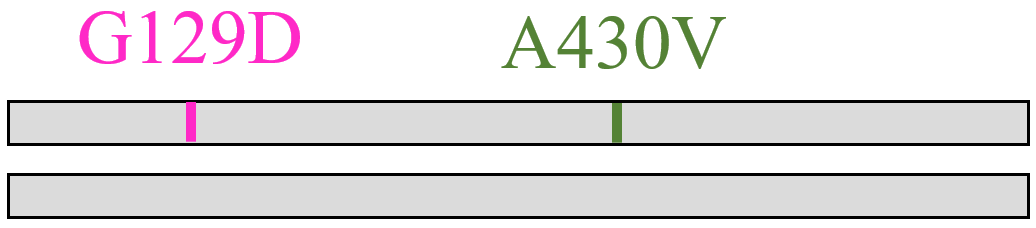 | ATGTCCTACAAGGAGGTCGCTCCCAAGCGCGAGAAGGATGCCGCCTCAGACGCCGCGGGTATAGCGGCGGTGCCTGAGGCGGCTGCCGCGGGAAATGATGACAAGTACATCCACCCCGAGGCAGCATCCCTGTTCGCCAGGTGCCCGTGTGCTCGCCGCGTCCCCGTGTTTGGTGACGCTGTCGAGGGCTACGGGCTCAAGTTCATCATCGCCCTAGGCGCTAGCAACCTGCTCTGCAAGGGTATCGCGGATCAGATTCTTACCGGTCAGACGTACGCCATGATGATTGATCGCTACGGCATCGACGTGGCCCGCTACCAGCGCCTGTCTTCGCTTTCGTCCATGGGGTGGTCCATCAAGGCCTTCACCGCGATGCTCTGCGACG**N**CTTCGCCTTCCTCGGCTACACGAAGCGCTGGTACATGTTCATCTCCTGCGTCGGCGGTGGTGTTTTCGCCCTCCTGTACGGCCTGCTGCCCGCGAAGGAGGCGTCGGCTGATGTGGCGTGTGCCTTCATCTTCCTGTCGTGCTGGGGCAAGGCCAACGTGGATATCCTGTCCCAGGGCCATTACAGTCGACTGATGCGCGAGAACCCGAAGCCTGGCCCGGCGCTGGTGAGCTGGATCTGGCTCTGGATCATGACCGGCTCGCTCATCGCGACTGTGATGAACGGCCCGCTCGCGGATGCCGGGAAGCCGCAGATCAGCATCTTCGTGTCTGCCGCGCTGCAGGCCATCACCTGCGTCTTCTACCTGTTCAACTGGTACGGGGAGAAGAAGAACCGCGTGCTGCGCTCCGAGGACGCGCTGTTTATTCTGGAGGAGACCCGCAAGGAGCGTGAGCGCCTGGGTCTCGAGGGGGTGTACGACGGCACGGCGGGTGCGCAGCATGGTGGTGCGGCGAAGGGGAAGAAGAGCCCGCAGCACTCGCACTCGGATGAGGACGTGGAGGGTGCCGTACGGGACGCCCTCAACGATGGTCAGCGCGACAACGGTGAACTTGTGCAGGACGTCTACGACGACGCGTATGACGACGGCGAGGGGGTGGCCGAGGGCGATGTGTACTACGGCAAGCCGCCGGTGCCGTGCCTGTTCGGGCTGTTCGAGGCAAACACGGAGGTGATTTCGAAGAACTGGAAGATCTTCGTGTACAGCGTCGTCATGACCTGTGCTGTGATCACGATGCTGTGTGCCAACATGCTGGCCGACACGCTGGGCCTCCTGGTTGCGTGCGTCGTTGTGTCGACCATCTGCTGTACCACGTCCTTCTGGGCCCTGCCGCTGGTGATTG**R**GAAGGCCAACGTGTTTGCATACCTGGATAAGGCTGTTTCCATCCGTGTGGGCGGTCCCCTAAATGCGTTCTACTTGAACACCTACCAGTGCCCTGGCAACCTGCCGAACTTCACCTACACCTTCTACAACACGGTGGCGGGCGTCATCAACACCATCGTTGGTATGATTACCGTGACGCTGTTCAACTTCCTGTTCGCGAAGCATGGCTACCGCCTCACCTTCATTGTGACGACAATCATGCAGGTTATGGGTGGTGTGTTCGACATCATCATTGTGAAGCGGTGGAACCTGTACATTGGCATCCCTGACCACGCCATGTACATCTGGGGTGATGCTGTTGTGGGTGAGCTCGTGTACATGCTTGGCTTTATGCCGCAGATCGTGCTGCTGTCTCGCCTGTGCCCTCGTGGCTCGGAGAGTGTCGTGTATGCGCTGATGGCGGGCTTCGCCCGCCTCGGCCGAACGACCGCGTCGTCCCTCGGTGCCATCCTTCTGGAGTACGGCCTGCCTGTGTTCAAGACCCAGGATGACGGGTCTCGCTGCAACTACGACAACCTGCCGCTGCTGTTGTTCGTGACCAGCATGTGCACGCCGCTGCTGGCGATTCCGCTGAGCATGATACTGCTTCCGAAGGCGCGCATCTGCGACGATATCGACGTTGACGGCAAGGTGGTGCGCCAGGCCGTGGATAAGCAGGTCGCAGCTGCTCCGCTGTCGAGCTCCGACTCGGACGCGGTGATGGCTGCCGAGCCGCTTCATGGGAACAAGGCGGATGAGCGCGAGGCAGCGCGCGGGGAGACGGTGCAGGGTAAACCCGCAGGCGGTTCAGAGAAATAG | G386A:  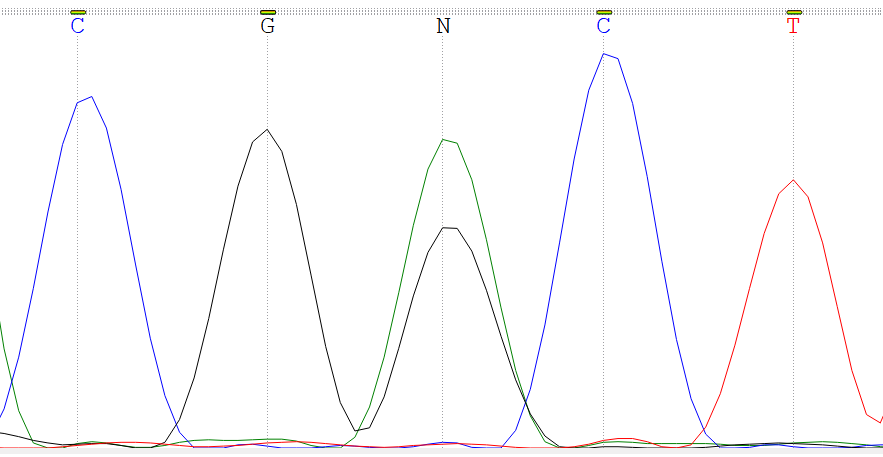  C1289T (RC):  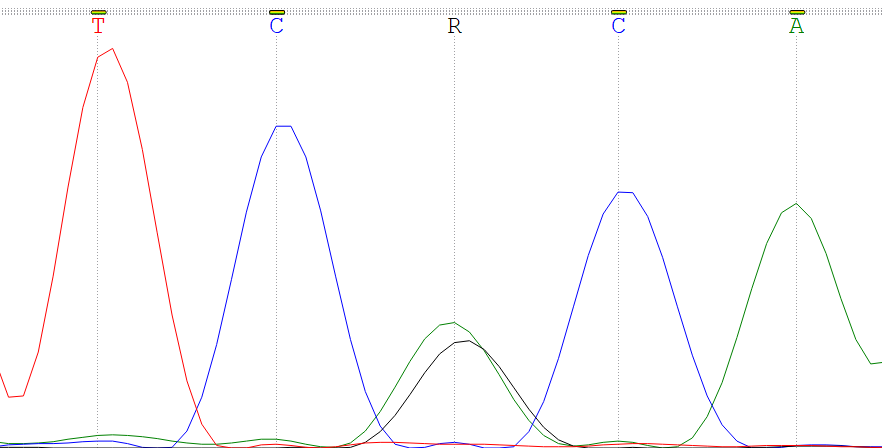 |
| LmjF.23.0270 | PTR1 | DNA:  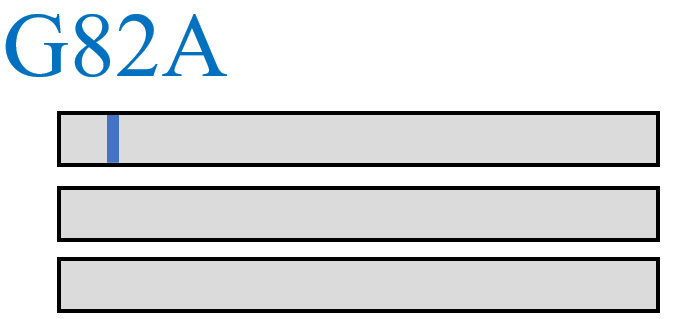  Protein:  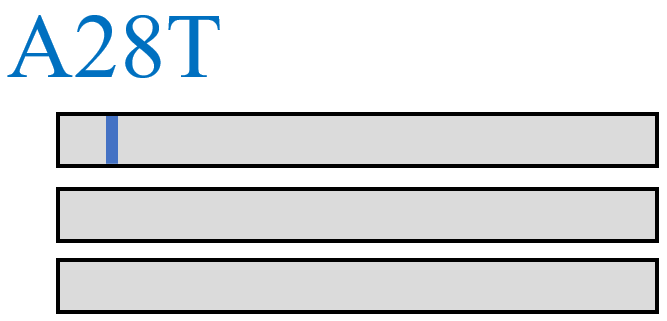 | ATGACTGCTCCGACCGTGCCGGTGGCGTTGGTAACAGGCGCCGCGAAGCGTCTTGGCCGCAGTATCGCTGAGGGACTCCAC**N**CGGAGGGGTACGCTGTCTGCTTGCACTATCATCGCTCTGCTGCAGAAGCGAACGCACTATCCGCGACGCTCAACGCAAGGCGACCGAACAGCGCCATCACGGTGCAGGCGGATCTGAGCAACGTTGCCACAGCCCCGGTCAGCGGCGCTGATGGCTCTGCACCTGTTACCCTCTTCACGCGCTGTGCTGAGTTGGTGGCTGCGTGCTACACCCACTGGGGACGCTGCGACGTGCTAGTGAACAACGCCTCTTCTTTCTACCCCACGCCGCTGCTGAGGAATGACGAGGATGGACACGAGCCCTGTGTCGGAGATAGAGAGGCAATGGAGACGGCCACCGCTGACCTCTTCGGCTCCAACGCGATAGCGCCCTACTTCTTGATTAAGGCGTTCGCGCATCGCGTCGCGGGCACCCCAGCCAAGCATCGCGGCACCAACTACTCCATCATCAACATGGTCGACGCCATGACGAACCAGCCTCTTCTCGGGTACACCATATATACCATGGCCAAAGGGGCGTTGGAGGGGCTGACACGGTCTGCCGCGCTGGAGCTTGCGCCGCTGCAGATTCGAGTGAACGGCGTTGGTCCGGGTTTGTCGGTGCTCGTCGATGACATGCCCCCTGCTGTGTGGGAGGGCCACCGCAGCAAGGTGCCTCTGTACCAGCGCGATTCCTCCGCCGCAGAGGTGAGCGACGTTGTTATCTTTCTGTGCTCCTCCAAGGCCAAGTACATCACCGGCACCTGTGTCAAAGTGGATGGTGGCTACAGCCTTACCCGGGCCTGA | 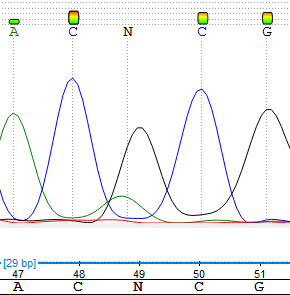 |
|  |  | DNA:  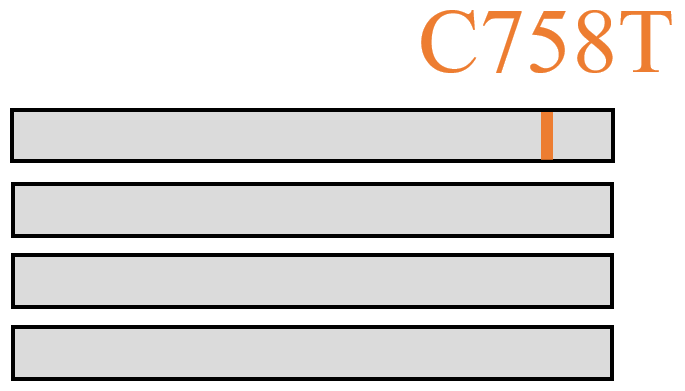  Protein:  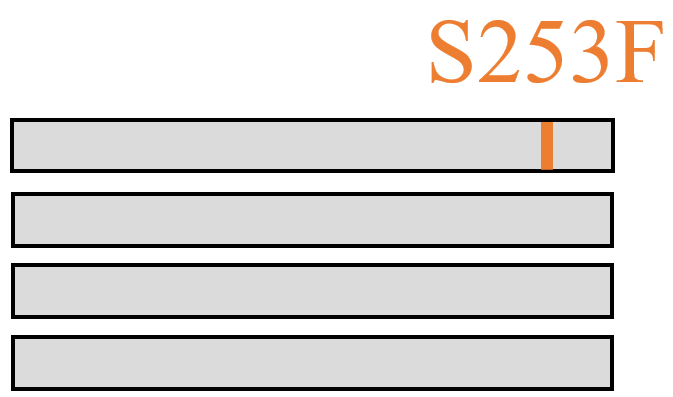 | ATGACTGCTCCGACCGTGCCGGTGGCGTTGGTAACAGGCGCCGCGAAGCGTCTTGGCCGCAGTATCGCTGAGGGACTCCACGCGGAGGGGTACGCTGTCTGCTTGCACTATCATCGCTCTGCTGCAGAAGCGAACGCACTATCCGCGACGCTCAACGCAAGGCGACCGAACAGCGCCATCACGGTGCAGGCGGATCTGAGCAACGTTGCCACAGCCCCGGTCAGCGGCGCTGATGGCTCTGCACCTGTTACCCTCTTCACGCGCTGTGCTGAGTTGGTGGCTGCGTGCTACACCCACTGGGGACGCTGCGACGTGCTAGTGAACAACGCCTCTTCTTTCTACCCCACGCCGCTGCTGAGGAATGACGAGGATGGACACGAGCCCTGTGTCGGAGATAGAGAGGCAATGGAGACGGCCACCGCTGACCTCTTCGGCTCCAACGCGATAGCGCCCTACTTCTTGATTAAGGCGTTCGCGCATCGCGTCGCGGGCACCCCAGCCAAGCATCGCGGCACCAACTACTCCATCATCAACATGGTCGACGCCATGACGAACCAGCCTCTTCTCGGGTACACCATATATACCATGGCCAAAGGGGCGTTGGAGGGGCTGACACGGTCTGCCGCGCTGGAGCTTGCGCCGCTGCAGATTCGAGTGAACGGCGTTGGTCCGGGTTTGTCGGTGCTCGTCGATGACATGCCCCCTGCTGTGTGGGAGGGCCACCGCAGCAAGGTGCCTCTGTACCAGCGCGATTCCT**C**CGCCGCAGAGGTGAGCGACGTTGTTATCTTTCTGTGCTCCTCCAAGGCCAAGTACATCACCGGCACCTGTGTCAAAGTGGATGGTGGCTACAGCCTTACCCGGGCCTGA | 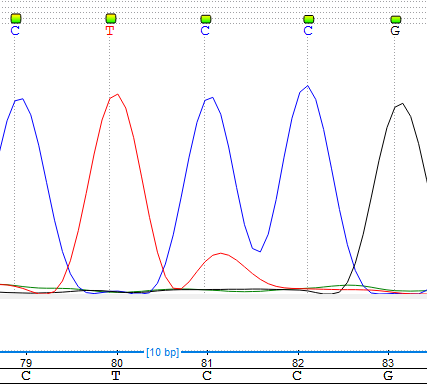 |

**^a^** Genes were sequenced by Sanger to confirm target mutation(s). Mutations are highlighted in red.

**^b^** Chromatograms shows the target mutation. RC, reverse-complement sequence.
